# Advances in Peptidomimetics for Next-Generation Therapeutics: Strategies, Modifications, and Applications

**DOI:** 10.1021/acs.chemrev.4c00989

**Published:** 2025-07-23

**Authors:** Lucia Lombardi, Valentina Del Genio, Fernando Albericio, Daryl R. Williams

**Affiliations:** † Department of Chemical Engineering, 4615 Imperial College London, South Kensington, London SW7 2AZ, U.K.; ‡ School of Biological Sciences, 1596 Queen’s University Belfast, 19 Chlorine Gardens, Belfast, BT9 5DL, U.K.; § Department of Pharmacy, University of Naples Federico II, Via Domenico Montesano 49, 80138 Naples, Italy; ∥ School of Chemistry and Physics, 9307 University of KwaZulu-Natal, Durban 4001, South Africa; ⊥ CIBER-BBN, Networking Centre on Bioengineering, Biomaterials, and Nanomedicine and Department of Organic Chemistry, University of Barcelona, 08028 Barcelona, Spain

## Abstract

Over the past two decades, peptide drug discovery has experienced a remarkable renaissance, with organic chemistry and biotechnology emerging as pivotal tools for developing peptidomimetics that exhibit improved stability, specificity, and bioavailability compared to conventional peptides. This review systematically examines methodologies for modifying peptide backbones to achieve targeted properties, highlighting recent advances facilitated by modern biotechnological innovations for novel molecular transformations. Additionally, the review emphasizes the practical applications of peptides and peptidomimetics, showcasing their successful integration into medicine and pharmacology. This manuscript evaluates achievements and challenges in the field and identifies critical areas for further research. Its overarching aim is to synthesize current knowledge and propose strategic directions for advancing peptide-based therapeutics.

## Graphical abstract

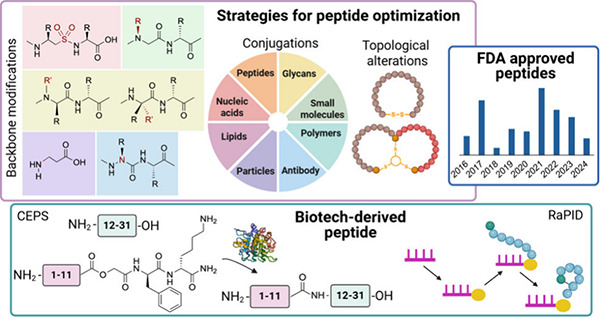

## 1 Introduction

In the field of biomimicry, scientists find inspiration in nature, emulating its native models and solutions to craft molecules and processes specifically tailored to address human challenges. This scientific pursuit involves building upon foundational knowledge, and strategically harnessing inspiration from natural molecular architectures and functional mechanisms.^1^ The widespread adoption of the strategy to replicate the structures and functions of peptides and proteins is a fundamental pillar in the dynamic landscape of drug design, discovery, and development. The significance of the field is underscored by a robust and continuously expanding body of scientific literature which is reflective of ongoing advancements that shape our understanding of these essential biological entities.^2^

Peptides and proteins are indispensable components of cellular entities, assuming crucial roles in vital biological processes, providing structural support to cells and tissues, facilitating signal transmission, regulating physiological functions, and contributing to the functioning of the immune system. The structural complexity of these biomolecules spans a spectrum, ranging from small peptides characterized by single secondary structures or random configurations to intricate assemblies of helices, sheets, and turns observed in more complex proteins.^3^ This diversity in structural complexity highlights the adaptability and versatility of peptides and proteins as they execute essential functions within the dynamic landscape of cellular biology.

The pharmaceutical industry has recognized the therapeutic potential of peptides in addressing unmet medical needs, positioning them as a valuable adjunct or even a preferable alternative to small molecules. Peptides play a transformative role in modern pharmaceutical research, serving as key drivers in the advancement of both biological and chemical sciences.^4^ Early 20th-century research efforts, which sought to unravel the structures and biological functions of peptide hormones like insulin, oxytocin, vasopressin, and gonadotropin-releasing hormones (Table 1, Table S2, Table S6), have led to numerous innovations in pharmacology, chemistry, biology, and technologies fundamental to current drug discovery processes.^4^ The discovery of insulin in 1921 marked a transformative milestone. Within a year, it transitioned from laboratory research to clinical application and subsequently emerged as the first commercially available peptide therapy in 1922. Another pivotal moment occurred in 1982 with the introduction of human insulin, exemplified by Eli Lilly and Company’s development of Humulin, the inaugural synthetic insulin manufactured through recombinant DNA technology. This advancement eventually resulted in discontinuing the original insulin product derived from animals, which had been used for six decades (Figure 1).^5^

**Figure 1:**
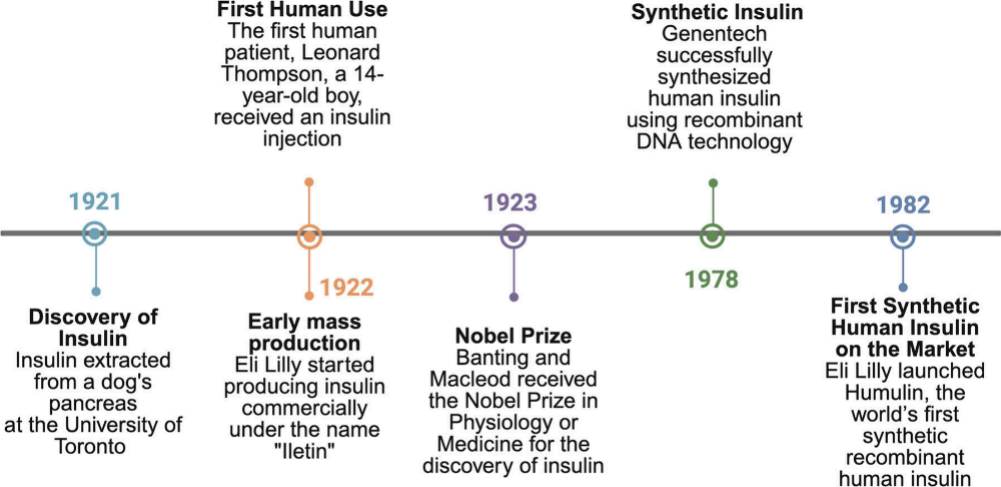
Insulin journey: from its discovery to the commercialization of the first synthetic human insulin. Created in BioRender.

**Table 1:** Peptide Drugs Launched on the Market for Blood Sugar and Weight Management

Generic name	Brand name	Drug class	FDA first approval year	Company	Therapeutic indication	Route^a^
insulin	Iletin	extraction	1923	Lilly	Type 1 and 2 diabetes	SC
insulin	Humulin, Novolin, Afrezza	recombinant	1982	Genentech, Lilly, Novo Nordisk, Chiron, Zymo, Wockhardt, Hoechst, Organon, Biobra, Mannkind	Type 1 and 2 diabetes	SC
lispro	Humalog	insulin analogue	1996	Lilly	Type 1 and 2 diabetes	SC
aspart	Novolog	insulin analogue	2000	Novo Nordisk	Type 1 and 2 diabetes	SC
glargine	Lantus, Basaglar, Toujeo Solostar	insulin analogue	2000	Sanofi, Lilly	Type 1 and 2 diabetes	SC
glulisine	Apidra, Apridra Solostar	insulin analogue	2004	Sanofi	Type 1 and 2 diabetes	SC
determir	Levemir	insulin analogue	2005	Novo Nordisk	Type 1 and 2 diabetes	SC
deglutec	Tresiba	insulin analogue	2015	Novo Nordisk	Type 1 and 2 diabetes	SC
pramlintide	Symlin	amylin analogue	2005	Amylin	Type 1 and 2 diabetes	SC
exenetide	Byetta	GLP-1 agonist	2005	AstraZeneca	type 2 diabetes mellitus	SC
exenetide	Bydureon BCise	GLP-1 agonist	2017	AstraZeneca	type 2 diabetes mellitus	SC
liraglutide	Victoza	GLP-1 agonist	2010	Novo Nordisk	type 2 diabetes mellitus	SC
liraglutide	Saxenda	GLP-1 agonist	2021	Novo Nordisk	obesity	SC
dulaglutide	Trulicity	GLP-1 agonist	2014	Lilly	type 2 diabetes mellitus	SC
albiglutide	Tanzeum	GLP-1 agonist	2014	GSK	type 2 diabetes mellitus	SC
lixisenatide	Adlyxin, Lyxumia	GLP-1 agonist	2016	Sanofi	type 2 diabetes mellitus	SC
semaglutide	Ozempic	GLP-1 agonist	2017	Novo Nordisk	type 2 diabetes mellitus	SC
semaglutide	Wegovy	GLP-1 agonist	2021	Novo Nordisk	obesity	SC
semaglutide	Rybelsus	GLP-1 agonist	2019	Novo Nordisk	type 2 diabetes mellitus	O
tirzepatide	Mounjaro	GLP-1 agonist	2022	Lilly	type 2 diabetes mellitus	SC

a SC: subcutaneous, O: orally.

Although peptides have a long history, by the end of the 20th century, they had largely been reduced to a niche pharmaceutical category because large-scale production was prohibitively expensive; thus, only peptide hormones effective at low doses were viable on the market. However, the past two decades have seen a significant revival in peptide drug discovery efforts. Since 2000, numerous noninsulin peptide drugs have been approved globally, with several achieving substantial market success.^5^ Concurrent advancements in recombinant biologics have also led to a renewed interest in peptides, as both fields share similar biological properties and scientific developments. These achievements have encouraged pharmaceutical companies to reconsider the potential of peptide drug discovery, resulting in a renewed wave of investment in this area.^6^ At present, around 140 peptide-based medications are available globally, with ongoing steady progress in the development of new peptide therapeutics. (Figure 2).^4,7–14^

**Figure 2:**
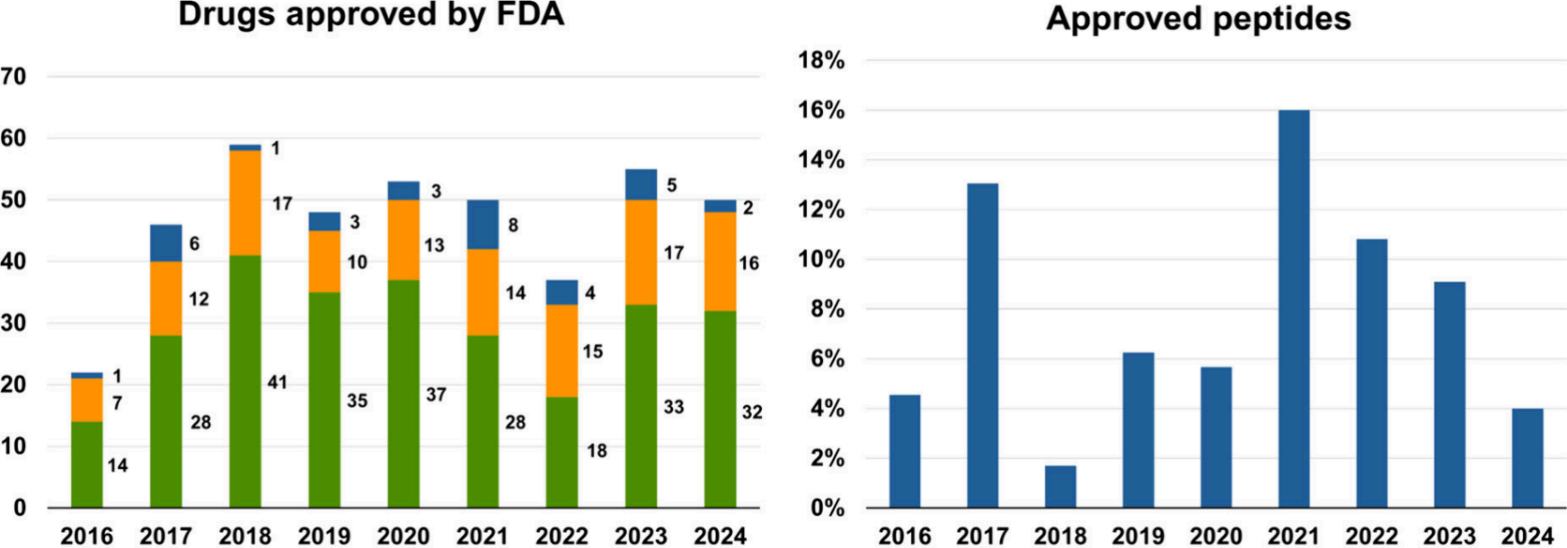
Left: Annual number of drugs approved by the FDA from 2016 to 2024, categorized as peptides (blue), biologics (orange, including monoclonal antibodies, enzymes, and antibody-drug conjugates), and other approved entities (green). Right: Percentage of peptides approved relative to the total number of approved entities each year. Created in BioRender.

FDA-approved drugs can be broadly classified into 2 major groups based on their nature and mode of action: small molecules and biologics. Small-molecule drugs contain up to 100 atoms and are very stable in several conditions. Biologics are therapeutics that come from living organisms and include recombinant proteins, monoclonal antibodies, cell therapies, vaccines and genes. Peptide therapeutics occupy a unique position in the pharmaceutical landscape, bridging the gap between small molecules and biologics (Figure 3).^15^

**Figure 3:**
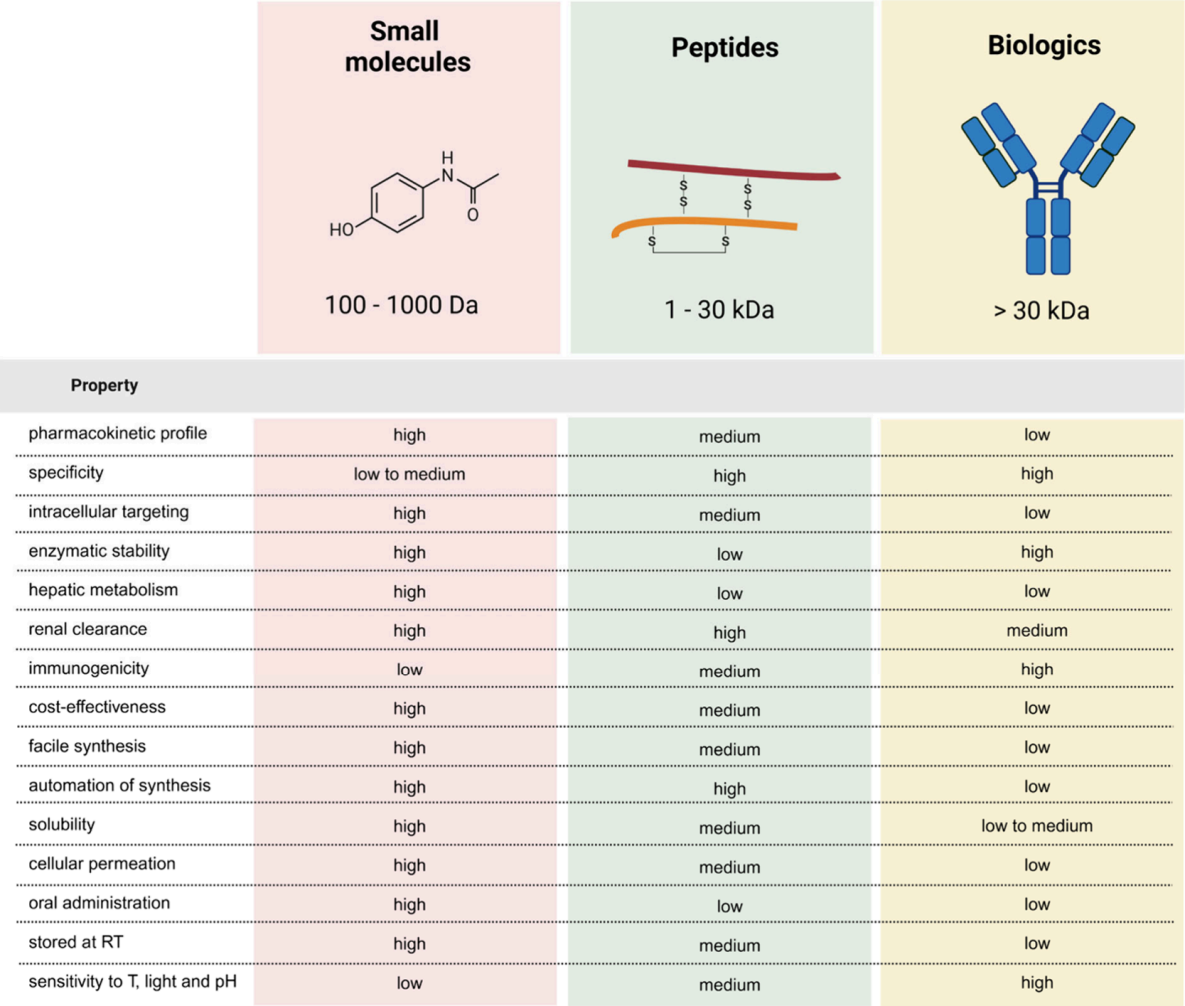
Drugs are categorized into three main classes based on their size and properties: small molecules (100–1000 Da), peptides (1–30 kDa), and biologics (>30 kDa). Small molecules, such as paracetamol, are considered highly druggable, as they can be administered orally and exhibit favorable pharmacokinetic profiles. In contrast, peptides (in the figure exemplified by insulin having two peptide chains connected by disulfide bridges) and biologics, such as antibodies, are less stable, less soluble, and more expensive to produce. However, peptides and biologics offer exceptional specificity, often demonstrating superior pharmacological efficacy. Created in BioRender.

Small molecules have long dominated the global drug market, benefiting from advantages such as cost-effectiveness, oral administration, and facile synthesis. Moreover, their inherent ability to penetrate cellular membranes widens the scope of biological targets that can be addressed. Conversely, peptides face challenges related to proteolytic instability and rapid clearance, which impact pharmacokinetic optimization and prevent tissue accumulation.^16^ Notably peptides offer significant advantages, including higher specificity and minimal hepatic metabolism, which is frequently associated with small-molecule drugs. Additionally, human dosage predictions for peptides using allometric scaling tend to be more straightforward compared to small molecules, making dose-ranging studies in clinical trials easier to conduct.^17^

The future of the peptide field looks promising, as continued scientific progress is set to overcome existing challenges and fully exploit the potent pharmacological potential of peptides in clinical applications and beyond.

Peptidomimetics is a class of compounds designed to imitate peptides by replicating specific physicochemical properties of certain amino acids or isolated secondary structures. Early examples of peptidomimetics focused on mimicking the primary structures of peptide hormones and protease substrates.^18^ In the past decade, peptidomimetics have gained recognition as valuable bioactive agents and promising drug candidates, especially in the area of targeting and modulating protein–protein interactions. This approach addresses challenges associated with traditional peptides, including limited enzymatic stability in the gastrointestinal tract and serum, inadequate absorption, rapid excretion through hepatic and renal pathways, compromised targeting capabilities arising from the intrinsic rotational flexibility of amino acids, and the potential for inducing antigenicity and unpredictable immune responses. Key factors like solubility, stability, bioavailability, and affinity play critical roles in pharmaceutical companies’ development of new drugs. Peptidomimetics, by offering a means to enhance these properties, presents a promising route for developing improved medicines.^19,20^

Antibodies have proven to be valuable treatments and provided enhanced specificity and significant pharmacokinetic benefits compared to peptides (Figure 3). However, challenges related to cost, solubility, stability, immunogenicity and bioavailability persist when developing these new therapeutics. In contrast, peptides offer advantages in this domain, including ease of production, cost-effectiviness, stability and reduced immunogenicity.^21^

This review article covers literature mainly from 2004 to 2024 and explores various modifications aimed at synthesizing peptidomimetics. A primary focus is placed on enhancing stability, binding affinity, and biological activity to develop more efficacious drugs. We systematically review methods for backbone modifications, starting with conservative approaches involving minimal alterations at specific positions along the peptide backbone. Various documented techniques for peptide backbone manipulation are explored, each tailored to achieve specific desired properties. The discussion also encompasses the recent advances leveraging modern biotechnological tools to create novel molecular transformations. The final section of the review will pivot toward practical applications of these molecules, especially peptides and peptidomimetics, to underscore the successful deployment of these molecules in various areas within the field of medicinal chemistry, medicine and pharmacology. Peptide applications in diverse fields, including diabetes, obesity, cancer, and infectious disease research, are highlighted. This review evaluates both the successes and limitations within the field to identify areas that require further investigation. Its ultimate goal is to consolidate the current understanding of the discipline and to propose potential pathways for future progress. Each topic has been investigated to the degree that benefits the broader scientific community. This review aims to be an accessible review of general interest to the chemistry community because it consolidates a vast array of relevant information, provides an in-depth analysis of key concepts, and critically evaluates advancements in the field. By synthesizing and presenting complex information in an accessible manner, it serves as a valuable reference for researchers, educators, and practitioners, fostering a deeper understanding and engagement within the chemistry community.

## 2 Strategies for Peptide Optimization

The optimization of peptide therapeutics necessitates a thorough multiparametric approach that evaluates the effects of each structural modification on key physicochemical properties, including potency, selectivity, stability, solubility, pharmacokinetic characteristics, and toxicity. Distinct differences exist between the optimization processes for peptides versus small molecules. Unlike small molecules, peptides can maintain high potency throughout the optimization process. Furthermore, the polymeric structure of peptides enables precise modifications at each residue, often leading to synergistic enhancements in overall performance when multiple local modifications occur.

Nonetheless, peptides possess intrinsic limitations concerning absorption, distribution, metabolism, excretion, and toxicity (ADMET) profiles. They typically demonstrate low absorption rates and limited plasma distribution and primarily undergo proteolytic metabolism characterized by amide bond cleavage in the backbone. Additionally, peptides are mainly excreted by kidneys, resulting in comparatively reduced toxicological risks.^15^

A general outline of the optimization process includes (Figure 4):

i. Determining the minimum active sequence: The peptide is subjected to iterative truncation of amino acids from both the C- and N-termini to identify the core sequence essential for the desired biological activity.
ii. Conducting positional scanning to identify critical residues: Traditionally executed using an l-alanine scan, positional scanning involves substituting each side chain with the smallest alternative while maintaining a similar conformational profile to evaluate its importance for biological activity. Recent advancements in synthetic and purification techniques have enabled scanning with a defined set of amino acids that exhibit diverse physical properties.^22^
iii. Shielding from degradation at the termini: Modifications to the C- and N-termini are performed to inhibit the degradative action of carboxy- and aminopeptidases, respectively. Commonly employed analogues include C-terminal primary amides and N-terminal acetylation; however, optimization toward unnatural analogues may be necessary if these modifications are not well tolerated.
iv. Identifying sites vulnerable to proteolysis: Initial exploration of the structure–activity relationship (SAR), along with pharmacokinetic experiments, stability assays, and metabolite detection, aids in pinpointing proteolytically susceptible amide bonds within the sequence.^23^
v. Enhancing proteolytic stability through backbone modification: Achieving proteolytic stability while retaining biological activity presents a significant challenge in peptide optimization. Various strategies are available for modifying labile amide bonds, but preserving the desired conformation and binding affinity can be complex.
vi. Formulation development: Appropriate formulations for the peptide drug are developed to ensure stability, ease of administration, and patient compliance. This process may involve selecting suitable excipients, dosage forms, and delivery routes.

**Figure 4:**
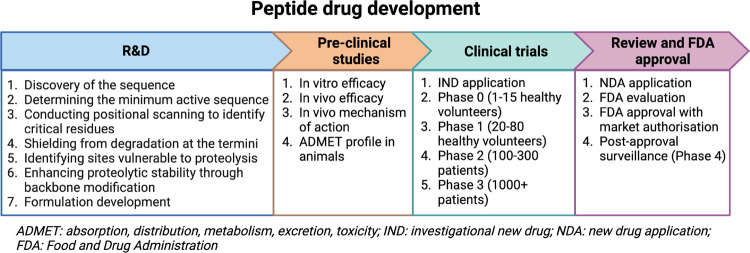
Development of a peptide drug begins with its discovery and optimization, which involves a series of chemical modifications to improve its stability, activity, and formulation. The peptide is then evaluated in vitro and in animal models to identify its ADMET profile before progressing to human studies, which are conducted in four phases known as clinical trials (Phase 0 to Phase 3). To initiate clinical trials, the sponsor must submit an IND (Investigational New Drug) application to the FDA. After Phases 1, 2, and 3, the sponsor must submit a report to the FDA. At the end of Phase 3, the sponsor may submit an NDA (New Drug Application) to request approval for market release, which will be evaluated by the FDA. Following commercialization, the drug is monitored to assess its therapeutic effects and potential side effects (Phase 4). While this description focuses on peptides, it is a general workflow applicable to the development of any drug. It always includes a research and development phase followed by preclinical and clinical studies, all of which are evaluated and approved by the FDA. Created in BioRender.

A mere 20 natural amino acids are crucial in various biological functions and structural variations. Post-translational modifications further enhance the intrinsic diversity in peptide and protein structure and function. The strategic addition of nonproteinogenic amino acids, along with several synthetic moieties and techniques, allows for a significant expansion of this diversity.^24^

The building blocks employed to modify the peptide backbone exhibit diverse structural variations. Some closely resemble native L-α-residues, while others bear little similarity to conventional protein backbones. In addition to structural diversity, there is considerable variation in the number and density of backbone modifications within a given chain. These modifications can be localized or can span a significant portion of the mimetics, with the option to create mimetics that feature an entirely artificial backbone (Figure 5). However, the latter approach is often unnecessary and can sometimes be undesirable, as peptides do not require a complete “transformation” into peptidomimetic forms.^25^ The more common strategy involves localized substitutions, which typically focus on a single amino acid or a short segment of contiguous backbone or side chains.

**Figure 5:**
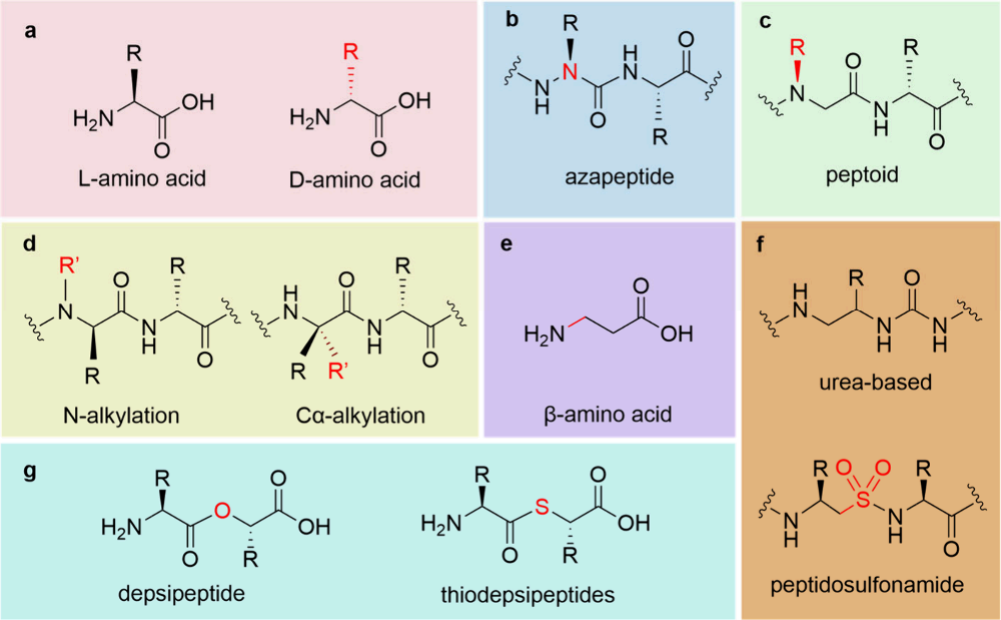
Different types of localized backbone modifications include changes in stereochemistry (except for glycine that lacks a chiral center), substitution of backbone atoms (such as in azapeptides, depsipeptides, and thiodepsipeptides), shifting of side chains (as in peptoids), addition of additional groups (e.g., alkylations), chain elongation (e.g., β-amino acids), and both elongation and substitution (e.g., oligoureas and peptidosulfonamides). Cyclization represents an additional form of backbone modification; however, this topic is addressed separately in Section 3.

Moreover, peptide-peptidomimetic hybrids present a sophisticated approach, allowing for the fine-tuning of binding affinity, resistance to proteolytic degradation, and clearance rates by balancing peptide and peptidomimetic components. Illustrative examples include peptoid-peptide, peptidosulfonamide-peptide, and urea peptidomimetic-peptide hybrids.^26–29^

Below, we present the most common strategies for introducing localized modifications in peptides.

### 2.1 Stereochemical Alterations

Although D-amino acids are infrequently encountered in nature, they assume significant roles in specific structures and biological activities (Figure 5a). For instance, they are crucial constituents for bacterial cell walls and antibiotics.^30,31^ Simultaneously, D-amino acids are indispensable for the functional integrity of various hormones and neurotransmitters in mammals.^32,33^ A peptide in which the chirality of amino acid residues transitions from L to D is termed an inverso analogue, representing the perfect mirror image of the original sequence. A retro-inverso peptide is a modified version of a peptide where both the sequence and chirality of the original peptide are reversed. The purpose of creating retro-inverso peptides is to design more stable peptides that resist degradation by proteases.^34^

Most experimental work involves the generation of peptides in which a single or a few amino acids are substituted with their corresponding D-version. D-amino acid substitutions have been used in several applications, including development of antimicrobial peptides, improvement of the enzymatic stability, enhancement of the antiangiogenic activity and anticancer action, assistance of the metal-based peptide bond hydrolysis and control of the peptide hydrogel degradation in cells.^35–39^

Instead of relying on a single or a few point mutations, Schumacher et al. developed a phage display technique to screen for and identify D-peptide ligands that exhibit resistance to proteolytic degradation.^40^ This method involves synthesizing proteins with D-amino acids to select peptides from a phage display library that expresses random L-amino acid peptides. Notably, they discovered that L-peptides could bind to proteins composed entirely of D-amino acids. Their findings suggested that an all-D-amino acid peptide could bind effectively to its natural protein counterpart made up entirely of L-amino acids. This implies that inverso analogs of peptides or proteins might replicate the structure and function of the originals, underscoring their potential as therapeutic molecules. Encouraging outcomes from these trials subsequently prompted the synthesis of more all-D-peptide fragments.^41^ Synthetic all-D-peptides have found application as mirror-image molecules in screening libraries of nucleic acids or genetically encoded proteins to identify specific binding ligands.^31^ Moreover, D-peptides exhibit diverse functions and showcase resistance to proteolytic degradation, garnering considerable attention in the field of drug discovery.

Modifying protein domains, particularly those featuring functionally relevant surface-exposed loops, is also feasible. This is exemplified by incorporating a native receptor-binding peptide loop onto a scaffold constructed with bridges of d-cysteine residues.^42^ Remarkably, this modification does not compromise tertiary folding but significantly enhances stability against protease degradation. Etelcalcetide (Parsabiv), a drug composed of a chain of seven D-amino acids with a d-Cys at the N-terminal forming a disulfide bond with an l-Cys, was approved for the treatment of hyperparathyroidism.^43^

A study conducted by Eberle et al. has demonstrated the potential of D-peptides in developing therapies for COVID-19.^44^ Despite the availability of several vaccines for SARS-CoV-2, there remains a critical demand for effective therapeutic options due to the lack of definitive treatments. Rather than targeting the interaction between the spike protein and cellular receptors, this study aimed to inhibit one of the key proteases involved in viral replication, specifically the 3CL protease, utilizing d-enantiomeric peptide ligands to disrupt its cleavage function. The research showed that a combination of competitive and noncompetitive D-peptides significantly enhanced the inhibitory effect on 3CL protease and displayed notable resistance to metabolic degradation over an 8-h period.

### 2.2 Cα Replacement

Substituting the α-carbon of an amino acid with nitrogen results in the formation of a semicarbazide, leading to the creation of peptides with semicarbazide, known as azapeptides. This modification replaces the rotatable Cα–C­(O) bond with a rigid urea Nα–C­(O), significantly altering the chemical and biological properties of the original peptide (Figure 5b). Specifically, this substitution eliminates chirality at the α-position, causing a shift in geometry from tetrahedral to trigonal. Computational and structural analyses reveal that these sequences adopt a β-turn geometry attributed to the planarity of the urea group and lone pair-lone pair repulsion of the hydrazine.^45^

Azapeptides exhibit heightened chemical stability and enzymatic resistance compared to amides, making them attractive drug design candidates.^46^ However, Fmoc-protected aza-amino acids, unlike natural amino acids, are either unstable or commercially unavailable. Consequently, synthesizing azapeptides necessitates additional steps to introduce aza-amino acids into peptide sequences.

Several synthetic pathways are available for incorporating the aza-amino acid residue. One method leverages hydrazine chemistry and peptide coupling, where the side chain of the aza-amino acid is built on a hydrazine derivative before coupling with a proteogenic amino acid (Scheme 1a).^47^ Alternatively, this process can be reversed, with the coupling occurring prior to side chain construction on the aza-residue. While the use of substituted hydrazines in synthesis can be tedious, progress has been made through the “submonomer approach”, where diverse side chains can be added to a common semicarbazone intermediate, specifically the semicarbazone-protected aza-glycine.^48^ The latter method for introducing an aza-residue in solid-phase synthesis can be outlined in three steps: (a) activation and coupling of the hydrazone, (b) chemoselective deprotonation and alkylation of the resulting semicarbazone, and (c) orthogonal liberation and aminoacylation of the semicarbazide (Scheme 1b).^45,49^ Extending submonomer chemistry beyond N-alkylation, the methodology has been expanded to prepare aza-arylglycines. Various aryl and heteroaryl iodides, including N-Boc-3-iodoindole and N-trityl-4-iodoimidazole, yield aza-indolyl- and aza-imazolylglycine residues, respectively, serving as acid-stable mimics of aza-Trp and aza-His. The addition of N-Aryl groups onto the semicarbazone is accomplished through Cu­(I)-mediated reactions.^45^

**Scheme 1:**
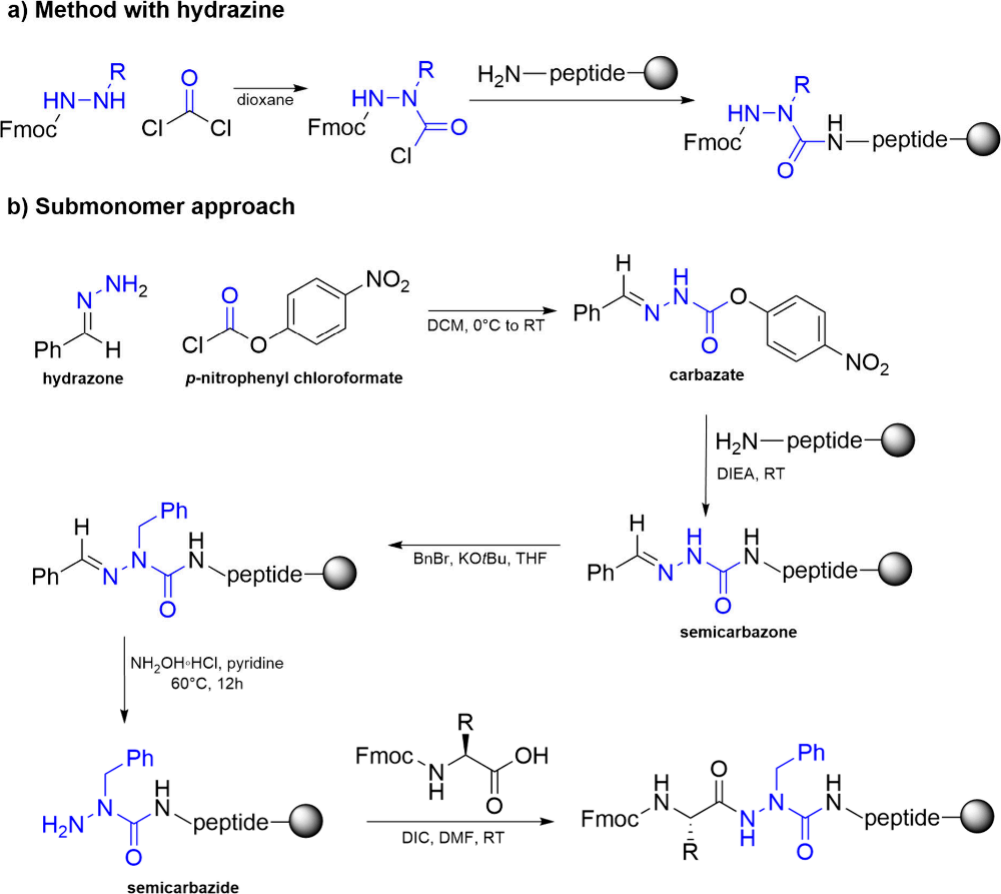
a) Method Using Hydrazine^a^ b) Submonomer Approach^b^

a Fmoc-protected hydrazine is converted into the corresponding carbazic acid chlorides with phosgene at room temperature. The aza-building block is activated to form a peptide bond.
b The hydrazone (benzaldehyde hydrazone in the scheme) is activated with *p*-nitrophenyl chloroformate to produce carbazate that is coupled to the peptide on a solid support. The product is a semicarbazone. A strong base (potassium *tert*-butoxide, KO*t*Bu) deprotonates the semicarbazone which is alkylated. In this example, the alkylation occurs with benzyl bromide (BnBr). The semicarbazide is liberated treating the semicarbazone with hydroxylamine hydrochloride (NH_2_OH) in pyridine. Amino acid was activated with diisopropylcarbodiimide (DIC) and coupled to the resulting semicarbazide.

Submonomer strategies have been adapted for other peptidomimetics, such as peptoids, streamlining the assembly of oligomers. This approach proves convenient for constructing libraries of azapeptides on the solid phase, overcoming challenges encountered in synthesis based on the activation and coupling of N-protected N’-substituted carbazate building blocks, such as oxadiazole formation. Moreover, this approach facilitates the facile addition of diverse functionalizations onto aza-residues, providing an avenue to explore various chemical reactions, including nucleophilic substitutions, [1,3]-dipolar cycloadditions, oxidation for pericyclic reactions, and Diels–Alder cycloadditions, enabling the incorporation of polar and charged side chains.^45^ While these methods have established a foundation for azapeptides, their applicability for library construction is limited due to the reagents and stringent conditions that may not be compatible with solid-phase peptide synthesis (SPPS).

Janda proposed leu-enkephalin mimetics in the form of pure azapeptides or azatides, which involveBoc-protected α-aza-amino acids coupled in a linear, stepwise, chain-lengthening fashion.^50^ Despite the existence of synthetic pathways for incorporating the aza-amino acid residue and conducting peptide synthesis, achieving pure azapeptide synthesis has proven to be a persistent challenge.

Altiti et al. introduced a new methods based on thiocarbazate building blocks as stable precursors for carbonyl-donating reagents and developed activation methods for these thiocarbazates for coupling (Scheme 2). Thiocarbazate building blocks are Fmoc-protected aza-amino acids and are easily incorporated into both solution-phase and standard SPPS protocols. They group also established protocols for incorporating these activated aza-amino acids demonostrating the can employ their methodology to systematically modify individual amino acids in peptides via an aza-scan approach, generating a small library of aza-amino acid-substituted peptide analogues for further biological evaluation.^51^

**Scheme 2:**
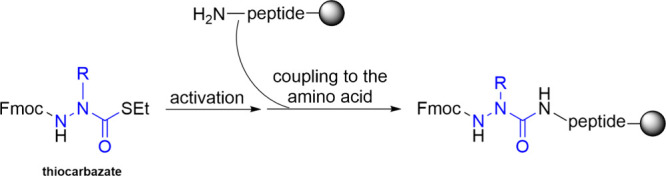
Thiocarbazate is a Building Block That Can Be Incorporated into the SPPS in a Very Practical Way Resembling the Common Incorporation of Amino Acids into the Peptide on Solid Support

### 2.3 Exploring N-Alkylation and Cα-Alkylation

Another strategy to enhance the enzymaric stability of peptides is the replacement of a natural α-residue with analogues featuring a methyl group on the N atom, known as N-Me-α analogues (Figure 5d). *N*-Methylation of amino acid residues is already present in nature and prevalent in nonribosomal peptides.^52^ Notable examples include cyclosporins, miuraenamides, lagunamides, and talaropeptides, among others.^53–56^

Drawing inspiration from the immunosuppressant cyclosporine with seven *N*-methylated peptide bonds (Figure S3), selective *N*-methylation has been utilized to produce membrane-permeable cyclic peptides overcoming some of the limitations associated with natural peptides by improving their stability, pharmacokinetic profiles, and overall bioavailability.

For many years, multiply *N*-methylated peptides were not favored by medicinal chemists due to the challenges associated with their synthesis. The primary obstacle lies in the steric hindrance at the *N*-methylated site, which complicates amino acid coupling. When Wenger achieved the first total synthesis of cyclosporine, he prompted renewed interest in their chemical production.^57^ Wenger carried out the synthesis in solution using Boc chemistry. Fortunately, cyclosporine lacks a diverse array of functionalized amino acids, allowing the difficult couplings at the *N*-methylated terminus to be accomplished via the formation of a reactive acid chloride.

Miller and Scanlan later introduced an efficient solid-phase synthesis approach, where free amines were activated with an *o*-nitrobenzenesulfonyl group, followed by direct methylation using methyl *p*-nitrobenzenesulfonate.^58^ Another solid-phase strategy involves the use of preformed *N*-methylated building blocks instead of in situ nitrogen alkylation.^59^ This method enables fast and efficient coupling of *N*-methylated amino acids through a fragmentation approach, using COMU and Oxyma as coupling reagents. Additionally, *N*-methylated building blocks are protected with Alloc rather than Fmoc (Scheme 3). The use of Alloc offers two key advantages: first, it is less bulky than Fmoc, facilitating coupling; second, it can be removed under neutral conditions, minimizing the risk of diketopiperazine (DKP) formation, which can occur when *N*-alkyl amino acids are the second residue on a CTC resin. To further improve coupling efficiency, lower-loaded resins are employed. Retratutide is a triple glucagon hormone receptor agonist (GLP-1, GIP, and GCGR receptors) and contains an N-Me-Leu residue (Section 6.1).^60^

**Scheme 3:**
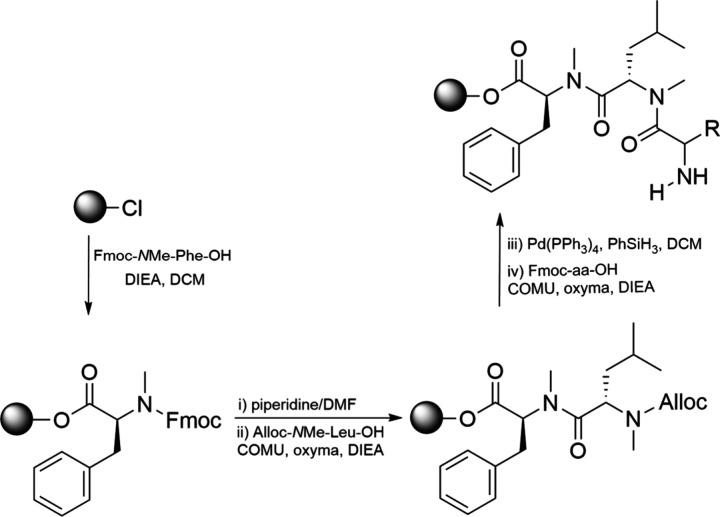
Attachment of the *N*-Methyl Amino Acid as the Second Residue on a CTC Resin^a^

a The *N*-methyl amino acid comes with Alloc to protect the amino functionality instead of Fmoc. The coupling occurs like a common SPPS with COMU and oxyma as activating reagents. Alloc is removed with tetrakis­(triphenylphosphine)­palladium(0), Pd­(PPh_3_)_4_ in the scheme, and phenylsilane (PhSiH_3_). After Alloc removal, the amino group is free and the following amino acid can couple as normally.

Advancements in genetic engineering have enabled the incorporation of *N*-methyl amino acids into peptides or proteins by expanding the genetic code. This involves reassigning specific codons to encode *N*-methyl amino acids, allowing for their incorporation by engineering tRNA (tRNA) during ribosomal protein synthesis. Through directed evolution and rational design, orthogonal tRNA–synthetase pairs have been developed to specifically recognize *N*-methyl amino acids and incorporate them into growing polypeptides. Through genetic engineering, the genetic code can be significantly expanded, allowing the incorporation of many non-natural amino acids beyond just *N*-methylated residues. In this section of the review, we have primarily focused on organic synthesis, while a separate paragraph is dedicated to biotechnological methods.

However, replacing the amide hydrogen with a methyl group disrupts both intramolecular and intermolecular hydrogen bonding, which may sffect the stabilization of bioactive conformations and recognition by receptors.^61^ in addition, the presence of the alkyl group on the nitrogen lowers the energy barrier to switch between *cis* and *trans* configuration stabilizing the *cis* configuration.^62^

Modifications are not limited to N-alkylation; inspired by nonribosomal peptide natural products, researchers are also developing peptides featuring N-amino (hydrazide) and *N*-hydroxy (hydroxamate) groups, characterized by NH_2_ or OH substituents on the backbone amide. These modifications have found applications in the development of optimally constrained folds and modulators of protein–protein interactions. Readers interested in a more detailed discussion are referred to the review by Angera et al.^63^

An alternative strategy involves attempts to rigidify amino acids by constraining the φ and ψ angles. For instance, proline inherently exhibits rigidity as its φ angle is constrained within a five-membered ring formed by the Cα-N bond. Over the years, various sophisticated approaches have been described to achieve such angle constraints, with well-known methods including the preparation of Freidinger lactam, spirolactams, and α,α-dialkylated amino acids, particularly α-methyl derivatives, which have been extensively studied.^64–71^

α,α-Dimethyl amino acids (Figure 5d), such as 2-aminoisobutyric acid or Aib, have been explored in various applications. For instance, they imparts increased stabilization, favoring the formation of a 3_10_ helix, which is more compact than the typical α-helix, featuring three amino acids per turn instead of the conventional 3.6 residues per turn.^72^ Natural peptides containing dialkylated amino acids, exemplified by alamethicin, a membrane-channel-forming peptide with several Aib residues, further underscore the practicality of these modifications.

In other examples, leveraging the replacement of one or two α-residues with rigidified Cα-methyl analogs has been instrumental in controlling dynamics, particularly in the study of intrinsically disordered sequences such as the activation domain from the p160 transcriptional coactivator for thyroid hormone and retinoid receptors.^73^ Similarly to N-Me-α analogs, α,α-dialkylated amino acids obstruct protease binding, making peptides more stable and better suited for use as drugs. Although α,α-dialkylated amino acids are now readily available commercially, they may present synthetic challenges due to increased steric hindrance requiring optimization of peptide synthesis protocols.^74^ By employing DIC, Oxyma, and microwave-assisted synthesis, it has been possible to synthesize a sequence of 17 consecutive Aib residues, leading to the first total synthesis of cephibol D, an antifungal peptide.^75^ Both semaglutide and tirzepatide contain Aib residues in their structures to improve their stability against proteases (Section 6.1).

### 2.4 Substituent Group Migration

Peptoids, a class of compounds pioneered by Zuckermann and colleagues, represent structural isomers of natural peptides wherein the side chains are shifted from the α-carbon atom to the amide nitrogen atom (Figure 5c).^76^ Notably, the side chains in peptoids, except for proline, are attached to the nitrogen, rendering peptoid monomers achiral. Compared to their peptide counterparts, peptoids exhibit significantly lower susceptibility to proteolytic degradation. Given this advantageous feature, it is not surprising that potential applications of peptoids have been extensively reviewed.^77–80^

While Zuckermann is credited with the formal discovery and development of peptoids, Bartlett and his colleagues had earlier explored the concept of N-substituted glycine derivatives as peptide mimics. They utilized N-(1-phenylethyl)-Gly and N-(methylimidazole)-Gly as monomers, mimicking phenylalanine and histidine, to construct a combinatorial array of N-substituted glycine oligomers.^81^ Zuckermann later formalized the concept of peptoids and developed a solid-phase synthesis strategy to rapidly generate peptoid libraries for drug discovery.^76^

Efforts to synthesize peptoid oligomers using the established SPPS method, with a preprepared set of Fmoc-protected monomers, presented challenges. This was due to the hindered nature of the secondary amine at the growing N-terminus in peptoid chains which leads to slower coupling reactions compared to the primary amines commonly found in SPPS. To address this limitation, Zuckermann developed a more efficient synthetic method for obtaining peptoids on solid phase, known as the “submonomer method”.^82^ This technique involves alternating acylation with bromoacetic acid and N,N-diisopropyl carbodiimide (DIC), along with nucleophilic displacement reactions of the bromide using primary amines (Scheme 4).

**Scheme 4:**
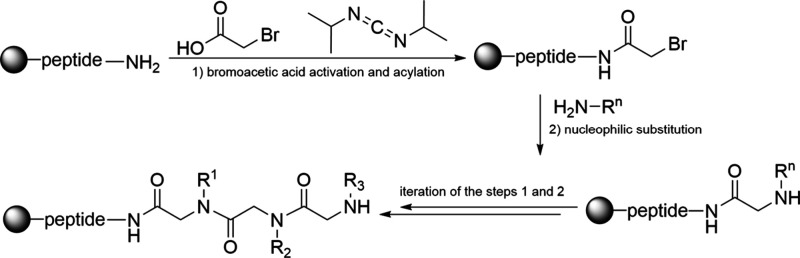
Synthesis of Peptoids Using a Submonomer Approach^a^

a The synthesis on solid phase proceeds with iteration of acylation and nucleophilic substitution and produces polypeptoids.

Peptoids hold significant relevance in the field of antimicrobial resistance. Extensive efforts have been directed toward developing peptoids that can mimic antimicrobial peptides (AMPs) with the aim of improving the poor pharmacokinetic profiles of the latter. The creation of peptide-peptoid hybrids has shown promising results in combating antibiotic-resistant bacterial pathogens, including *Staphylococcus pneumonia, Pseudomonas aeruginosa*, *Escherichia coli*, *Staphylococcus aureus*, and *Bacillus subtilis*.^83,84^ In addition, peptoids can be used to create advanced materials exploting their ability to fold and self-assemble and build large combinatorial libraries to identify protein ligands.^85,86^

### 2.5 Backbone Extension

The creation of novel peptidic oligomers, distinguished by a wide array of constitutional and configurational isomers, is accomplished by introducing additional atoms between the carboxyl and amino groups of amino acids. Seebach and Gellman were at the forefront of synthesizing extended peptides mainly derived from β (Figure 5e) or γ-amino acids, demonstrating their ability to adopt secondary structures such as helices, sheets, and turns, thereby exhibiting “protein-like” behavior and opening the research area of “foldamers”.^87–91^ These sequences not only showed resilience to proteolytic enzymes but also displayed enhanced pharmacokinetic properties. This has proven effective when applied strategically within α-helices, as demonstrated by the periodic substitution of α-amino acid residues with corresponding β-amino acid residues in the parathyroid hormone inverse agonist, PTH(7–34).^92^ This modification resulted in the analogue peptide α/β-PTH(7–34), which preserves the antagonist and inverse agonist activities of the original α-peptide while exhibiting increased stability against aggressive proteolytic enzymes. These outcomes highlight the potential of PTH-derived peptides with backbone modifications as valuable tools for examining the mechanisms of PTH metabolism and offer new prospects for developing therapeutics aimed at conditions driven by abnormal ligand-dependent or ligand-independent activity of PTHR1.

The additional carbon–carbon bond in β-amino acids increases the flexibility of the peptide bond, which can be a disadvantage when designing peptide drugs that need to bind to a specific protein site. However, when the β-carbon (the carbon adjacent to the nitrogen) in backbone-extended amino acids is replaced with oxygen, a more rigid conformation is observed (Figure 6a). This rigidity is due to the lone-pair electron repulsion in the N–O bond. Due to this stability, α-aminoxy acids hold potential in peptide drug design. The lone-pair repulsion between the heteroatoms in α-amino acids leads to the formation of a stable eight-membered-ring hydrogen bond between the amino acid and adjacent residues, known as the N–O turn (Figure 6b).^93^

**Figure 6:**
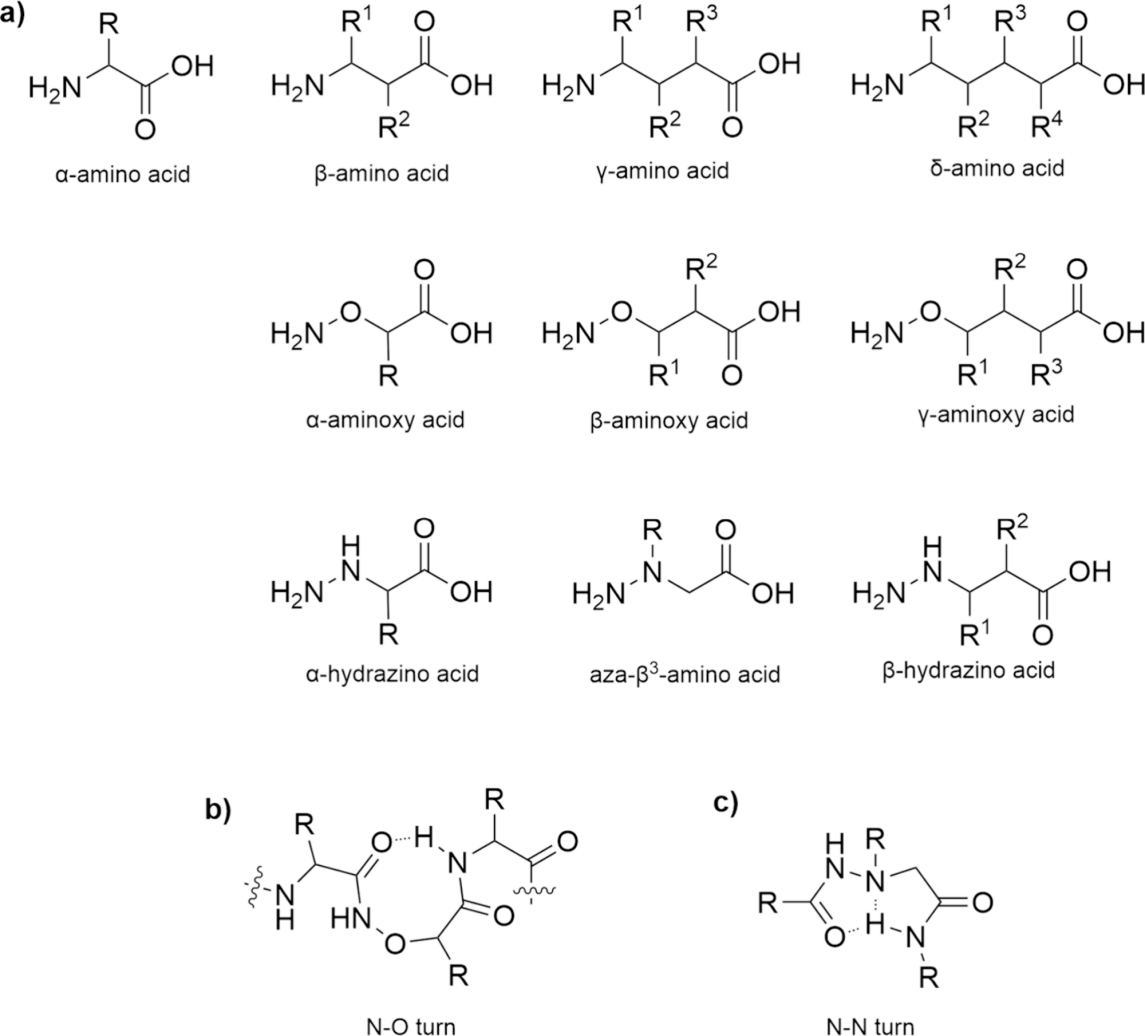
a) Structures of the different amino acids with extended backbone. b) the N–O turn formed with aminoxy acids and c) the N–N turn formed instead in the presence of hydrazine acids.

Yang et al. demonstrated that oligomers of α-aminoxy acids can form a highly stable 8-helix structure, facilitated by the N–O turn.^94^ This helical stability can be utilized to develop cell-penetrating peptides. Specifically, a hybrid peptide composed of D-α-aminoxy acids and L-α-amino acids has been shown to cross cell membranes through direct translocation.^95^ In contrast, β-aminoxy acids, possessing an additional carbon atom compared to α-aminoxy acids, exhibit more flexible structures due to the diversity of backbone extensions and substitution possibilities.^96,97^

The replacement of the β-carbon nitrogen introduced another category of peptidomimetics known as hydrazino acids (Figure 6a). In peptides containing hydrazino acids, the repulsion of lone electron pairs imparts rigidity and promotes an intramolecular hydrogen bonding pattern that facilitates unique turns. Specifically, in aza-β3-amino acids (hydrazino acids with an alkyl substituent on the extra nitrogen atom), a bifurcated intramolecular hydrogen bond forms between the carbonyl acceptor (CO_i_) and the nitrogen donor (NH_i+2_), creating an eight-membered ring. The hydrogen bonding interaction is further stabilized by the lone pair participation of neighboring nitrogen atoms (N_i+1_).^98^ This structure is referred to as the hydrazino turn or N–N turn (Figure 6c). Due to the rapid pyramidal inversion of the nitrogen, N–N turns are less rigid than N–O turns. However, when the aza-β3-amino acid is part of a small ring, its configuration and chirality are preserved.^99^

Aza-β3-amino acids can be synthesized from N^α^-substituted-N^β^-protected hydrazine and esters of bromoacetate (Scheme 5). However, this reaction typically yields a low output (36–50%). An alternative approach involves the reductive amination of glyoxylic acid with N^α^-substituted-N^β^-protected hydrazine to obtain the desired amino acid.^100^

**Scheme 5:**
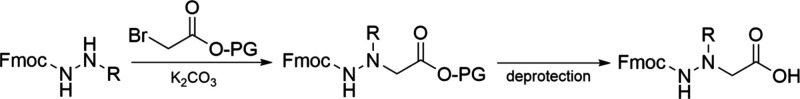
Synthesis of Aza-β3-amino Acids from *N* ^α^-Substituted-*N* ^β^-protected Hydrazine and Esters of Bromoacetate

Hydrazino-based peptidomimetics have shown promising biological activities, such as acting as protease inhibitors and antimicrobial molecules.^101,102^

Suga and colleagues developed a biotechnological method to synthesize a peptide library on ribosomes that includes both α-aminoxy and α-hydrazino acids. Since β-amino acids are much less effective substrates for ribosomal peptide synthesis compared to α-amino acids, consecutive elongation is particularly challenging. Their work successfully demonstrated the incorporation of α-aminoxyacetic acid and L-α-hydrazinophenylalanine during ribosomal translation using the tRNAPro1E2/EF-P system.^103^

### 2.6 Introducing Chemical Bonds and Carbonyl Replacement

Urea-based peptidomimetics, also known as oligoureas, represent a class of mimetics wherein a nitrogen moiety replaces the α-carbon of γ-amino acid residues (Figure 5f). Oligomeric structures composed of repeating urea linkages are named N,N′-linked oligoureas. Due to the presence of two NH groups per urea unit, these oligomers form a stronger yet tunable hydrogen bonding network in diverse compound classes, including biologically active and self-assembling molecules.

Within this category, aliphatic N,N′-linked oligoureas fall under the foldamer family. These oligoureas show a strong tendency to form stable helical structures in aqueous environments, establishing a predictable relationship between the primary sequence and the specific arrangement of side chains along the helix. The canonical oligourea helix, which features 2.5 residues per turn, presents a side chain configuration that, when viewed from above, resembles a five-pointed star spanning two turns. Guichard and co-workers have shown the ability of oligoureas to substitute the α-helix in a zinc finger domain.^104^ In fact, these mimetics adopt a native-like conformation featuring a metal-binding site, allowing them to interact with double-stranded DNA. The interaction is primarily facilitated by contacts with the substituted α-helix in the original protein, underscoring the effective structural mimicry of this protein segment.

Strategies for synthesizing urea peptidomimetics have been developed using solid-phase methods, incorporating both Boc and Fmoc protection strategies.^105,106^

Peptidosulfonamides are another class of peptidomimetics where a sulfonamide replaces the carbonyl amide. However, the S–N bond in sulfonamides is not as strong as the amide bond. In fact, it can be unstable and undergo hydrolysis under acidic or basic conditions. This instability occurs because sulfonamides lack the resonance stabilization found in peptide bonds. Introducing an additional −CH2– group results in the preparation of aminoethanesulfonic acid building blocks, which help to achieve stable derivatives that are resistant to fragmentation (Figure 5f).^107^

Large-scale synthesis of β-substituted aminoethanesulfonic acid building blocks is feasible, and these are utilized in the assembly of β-peptidosulfonamides.^108,109^ As the β-aminoethane sulfonamide residues act as potent helix or β-strand disruptors, these oligomeric peptidomimetics exhibit relatively high flexibility and do not adopt well-defined structures.

### 2.7 Expanding the Toolbox for Mimetic Design with Isosteric Groups

Another approach involves substituting particular chemical moieties with isosteres—entities that possess similar electronic distributions and physical properties. This local modification primarily focuses on single amino acids and includes replacements of backbone, side chain, and dipeptide isosteres. In this section, we focus on the peptide bond isosteres.

Various peptide bond isosteres have been reported, providing numerous options for enhancing proteolytic stability and biological activity.^110^ A noteworthy subset involves replacing the amino functionality with an isosteric atom, such as oxygen (resulting in depsipeptides) or sulfur (yielding thiodepsipeptides) (Figure 5g). These modifications significantly influence the secondary structure and folding properties of peptides by altering hydrogen-bonding patterns.^111^

Depsipeptides, present in nature and isolated from various microorganisms like bacteria and fungi, manifest diverse antimicrobial activities with a broad spectrum of action. Notable examples include valinomycin, which functions as a potassium-selective pore, and nonactin, which selectively acts as a pore for ammonium.^112,113^

Since the initial identification of natural depsipeptides, numerous methodologies for synthesizing their synthetic counterparts have been documented.^114^ Ester bonds can be formed not only on the backbone but also on the side chains, utilizing the OH side groups of serines and threonines. If the ester bond is formed on the backbone, the OH group should be added first. Common methods to achieve this involve activating the carboxylic acid group and then reacting it with α-hydroxy acids.^115^ This esterification process can be carried out using various coupling methods, such as DIC/DMAP, PyBroP/DIEA, and *N*-Hydroxysuccinimide.^116–119^ Additionally, Mitsunobu esterification offers an alternative approach by activating the alcohol rather than the carboxylic acid.^120^

Depsipeptides are highly effective therapeutics, particularly against infections. Beyond their use in therapeutics, depsipeptides serve as excellent peptidomimetics with a range of applications. For example, depsipeptides made with Ser or Thr linked to the peptide backbone via an ester bond can function as peptide switches. These peptide switches have been utilized to functionalize alginate hydrogels, where they rearrange upon enzymatic cleavage to expose the YIGSR sequence, which binds to integrins on the cell membrane.^121^

Additionally, depsipeptides are valuable in the synthesis of “difficult peptides” due to their ability to disrupt the continuity of hydrogen bonds in the peptide backbone, preventing aggregation during peptide synthesis. In this strategy, the amino acid following Ser or Thr is not attached to their N-terminus but to their side chain, where the OH group can be selectively removed during SPPS (Scheme 6). The coupling performed with common activating reagents results in the formation of an ester bond. The following amino acid is then coupled to the amino function of Ser or Thr after Fmoc deprotection. Once the peptide synthesis is complete and the peptide is cleaved, mild basic aqueous conditions promote the O→N shift, resulting in a classic peptide bond and the release of the free Ser or Thr.^122^

**Scheme 6:**
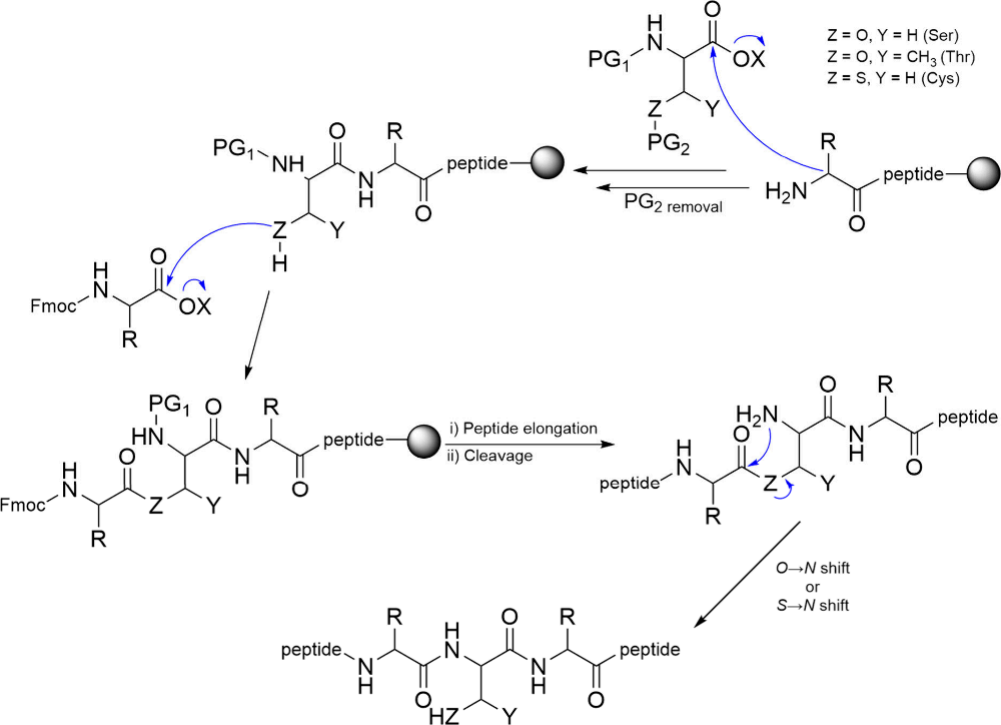
Synthesis of Depsipeptides and Thiodepsipeptides and Spontaneous Formation of Peptide Bonds in Aqueous Solution

Thiodepsipeptides are naturally occurring compounds formed through a thioesterification process, in which a cysteine thiol group reacts with the carboxylic group of amino acids or hydroxy acids. An example is the macrocyclic thiodepsipeptide thiocoraline, a potent antitumor agent isolated from *Micromonospora sp*. and *Verrucosispora sp*.^123^

Thiodepsipeptides can be synthesized in a similar manner, but instead of using Ser or Thr, Cys is incorporated. At mild basic pH, the S→N rearrangement is also observed. Although thiodepsipeptides are less stable than depsipeptides, making them less suitable for drug development, they are highly valuable as intermediates in chemical synthesis.^124^

### 2.8 Conjugations to Enhance the Half-Life of Peptide and Protein Drugs

While some therapeutic proteins, such as antibodies, inherently possess extended half-lives, many endogenous molecules, including peptide hormones, are susceptible to enzymatic degradation, renal clearance, and rapid receptor-mediated elimination, resulting in a short plasma half-life (hereafter referred to as ‘half-life’). Consequently, considerable research efforts have been focused on developing diverse strategies and technologies aimed at prolonging the half-lives of peptides.^125^

Polyethylene glycol (PEG) conjugation, commonly referred to as PEGylation, increases the hydrodynamic volume of the conjugate, which helps to prevent renal clearance and enhances the pharmacokinetic profiles of biopharmaceuticals.^126^ Glycoengineering, or the conjugation of biopharmaceuticals to complex carbohydrates, has also garnered significant interest over the years.^127^ Both strategies often result in heterogeneous products due to the polydisperse nature of PEG and carbohydrate polymers.^128,129^

Similarly, prolongation of the half-life can be achieved by fusing or conjugating a peptide or protein with hydrophilic peptides, such as XTEN, unstructured biodegradable peptides, or sequences rich in Pro, Ala, and Ser amino acids (PAS tail).^130,131^ XTEN, genetically fused to the biopharmaceuticals, 864-amino acid peptide, is rich in Ala, Gly, Glu, Pro, Ser, and Thr residues. It is highly soluble, lacks a defined secondary structure, and has a low tendency to aggregate. Additionally, shorter XTEN variants have been studied for various applications.^132,133^ The PAS tail is also highly soluble in physiological solutions and adopts a random coil conformation, demonstrating excellent stability in plasma.^134^ In contrast to PEGylation, XTENylation and PASylation produce a more homogeneous product.

The versatility of peptide conjugation extends across various fields, including biomedical research, drug development, diagnostics, and therapeutics. In drug discovery, conjugation can involve a pharmacologically active peptide combined with another active molecule to modify the pharmacokinetic properties, or a peptide may be utilized as a targeting or transmembrane delivery vehicle. The direct conjugation of compatible active agents presents significant advantages in clinical development, positioning peptides as promising candidates for this purpose. Peptides can be conjugated to a variety of molecules, including (Figure 7):

- Small molecules and imaging agents: Peptides can be tethered to drugs, imaging agents, fluorophores, radiotracers, contrast agents, toxins, or chelating agents, serving purposes in therapeutics, diagnostics, and research.^135–137^

- Antibodies and antibody fragments: These facilitate targeted drug delivery, imaging, or immunotherapy by directing them to specific cells expressing the corresponding antigen.^138,139^

- Lipids: They enhance cellular uptake, membrane insertion or stability, and are applicable for drug delivery, cell-penetrating peptides (CPPs), or membrane-targeting peptides.^140–142^

- Nucleic acids: Efficacy for gene regulation or therapy.^143^
- Nanoparticles: They improve targeting, cellular uptake, or controlled release properties for drug delivery, imaging, or diagnostics.^144^

**Figure 7:**
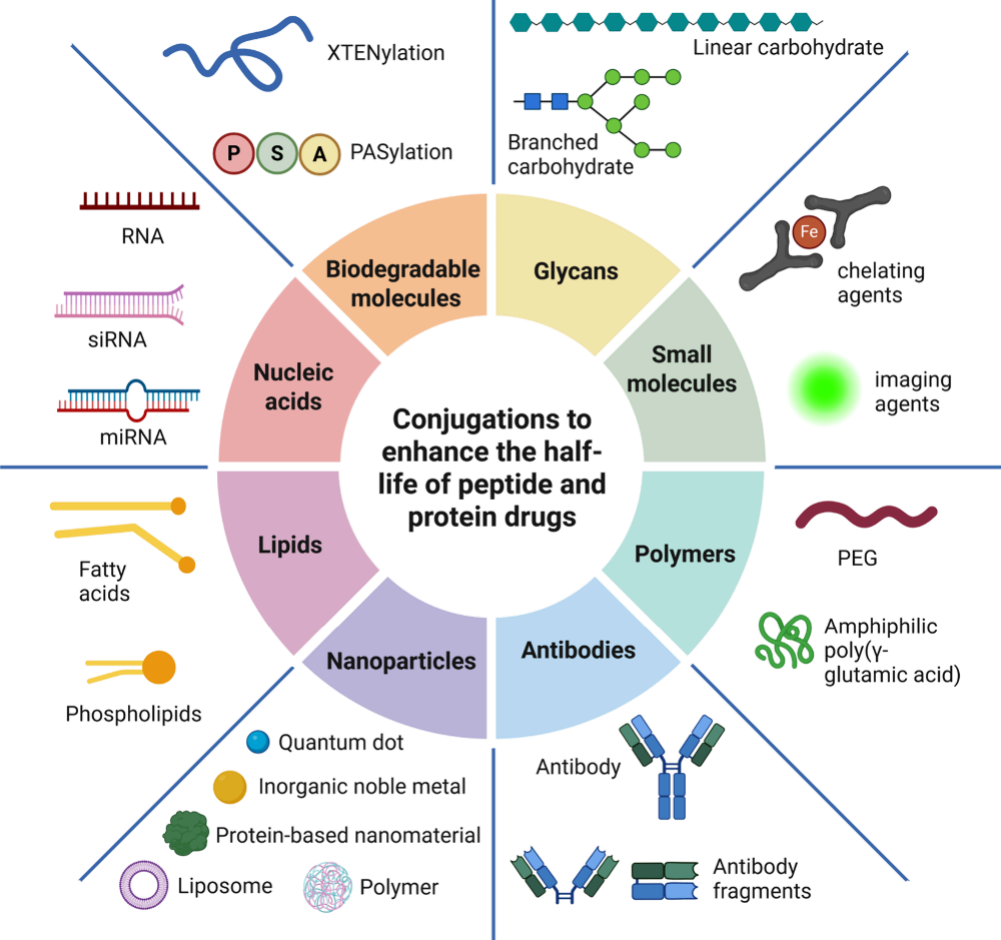
A peptide drug can be conjugated to various molecules to enhance properties such as enzymatic stability, plasma half-life, and target specificity. Common conjugates utilized in the pharmaceutical field include biodegradable entities such as lipids, proteins, glycans, antibodies, and nucleotides, as well as inorganic compounds like metal nanoparticles and chelated metals. Created in BioRender.

Noncovalent binding with albumin has been effectively employed to extend the half-life of peptides by attaching various ligands, such as fatty acids or antibody domains targeting albumin, known as AlbudAb, to the peptides or miniproteins.^145^ Because albumin is abundantly present and possesses a natural ability to carry fatty acids as reversible ligands, harnessing this characteristic highlights the potential of fatty acid derivatization and subsequent binding to albumin in prolonging the action profile of peptide drugs. As a result of these advancements, once-daily and, more recently, once-weekly formulations of several peptide drugs currently on the market or in development have been achieved (see antidiabetic peptides in Section 6.1). These formulations have largely addressed the therapeutic challenges associated with short half-lives of peptide drugs.^146^ Once-weekly therapies offer significant advantages, including enhanced convenience, improved treatment adherence, and better health-related quality of life. They also contribute to a reduced sense of burden associated with managing chronic conditions.

The process of fatty acid derivatization, commonly referred to as “lipidation”, has undergone meticulous optimization to tailor peptide-based or protein-based therapeutics with precise modifications.^147^ It has been theorized that subcutaneously administered peptide drugs, upon undergoing fatty acid derivatization, may exhibit prolonged retention at the injection site compared to their nonderivatized counterparts. The retention mechanism in this context likely involves interactions between the fatty acid side chain and albumin located at the injection site. Consequently, it is expected that the rate of diffusion within the tissue postinjection, as well as the transit across the capillary wall, will be reduced due to the increased molecular size of the albumin-peptide complex.^148^

Furthermore, it has been postulated that fatty acid derivatization may enhance the self-association of peptide drugs by promoting hydrophobic interactions among peptide monomers.^149^ This increased self-association would result in diminished absorption rates from the subcutaneous tissue, as the larger aggregates would have a greater molecular size than the monomers, thus leading to slower diffusion through the tissue and across the capillary wall. In circulation, the larger size of the complexes could protect the bound peptide from renal clearance and reduce the rate of distribution to extravascular compartments.

Chemically, the fatty acid derivatization of target molecules can be accomplished through several methods, including the direct coupling of fatty acids to the peptide backbone or via a linker and/or spacer. The linker, which connects the fatty acid to the spacer or the peptide backbone, plays a pivotal role in modulating binding affinity for the target receptor.^150^ The presence of a spacer can influence receptor binding; longer spacers may mitigate the negative effects on receptor interactions, while shorter spacers or the absence of a spacer can provide protection for the peptide against degradation, thereby contributing to longer half-lives.^146,151^

Fatty acids, particularly those characterized by long alkyl chains with a single carboxylate group at the distal end (monoacids), have been well-documented for their strong affinity for binding to albumin, with binding strength correlating positively with the length of the alkyl chain.^152^ Initially, fatty monoacids were used to mimic the transport of endogenous fatty acids, followed by the introduction of fatty diacids, which provide enhanced affinity for albumin and subsequently longer half-lives due to the presence of an additional carboxylic group at the end of the alkyl chain. The affinity for albumin is positively correlated with the length of the fatty monoacid or diacid. Among the fatty acids tested, 1,18-octadecanedioic acid (C_18_ diacid) and 1,20-eicosanedioic acid (C_20_ diacid) exhibited the highest binding affinities for albumin.^146,151^ The increased hydrophobicity of fatty monoacids compared to fatty diacids affects the solubility, receptor pharmacology, and biophysical properties of the derivatized molecule. Moreover, the derivatization of peptides with fatty monoacids enhances their association with cell membranes, promoting internalization—an attribute that has been recognized for decades.^153^ In contrast, fatty diacids possess an additional carboxylic group that enhances their solubility, resulting in fatty diacid-derivatized peptides being less likely to associate with cell membranes and undergo internalization. Consequently, incorporating fatty diacids generally aids in maintaining *in vivo* efficacy, as the target peptide or protein is less susceptible to loss due to hydrophobic interactions with cellular surfaces.

Another of the most effective and widely utilized strategies for extending the half-life of therapeutic proteins or peptides involves fusing or conjugating them with the Fc domain of immunoglobulin G (IgG) or with albumin. This approach increases the oral administration and molecular size of the therapeutic agent, resulting in reduced renal clearance and enhanced half-life due to cellular recycling mediated by the neonatal Fc receptor (FcRn).^154^ At physiological pH, FcRn exhibits a low binding affinity for albumin and IgG at the cell surface. However, upon internalization of the complex, the binding affinity of FcRn for both proteins increases within the acidified environment of endosomes, thereby protecting them from lysosomal degradation. As a result, albumin and IgG are recycled and released from FcRn at the cell surface, thereby extending their circulation time in the bloodstream.^155^ Moreover, engineering modifications in both the Fc domain and albumin to enhance their binding affinity to FcRn at a pH of 6 provide opportunities to further extend their half-lives beyond those of native Fc and albumin.^156,157^ These modifications can optimize the therapeutic efficacy of proteins and peptides by promoting sustained circulation and improved pharmacokinetic profiles *in vivo*.

#### 2.8.1 Conjugations Techniques

Numerous coupling reactions exist to facilitate the connection of these molecules, with several examples provided below (Scheme 7).

**Scheme 7:**
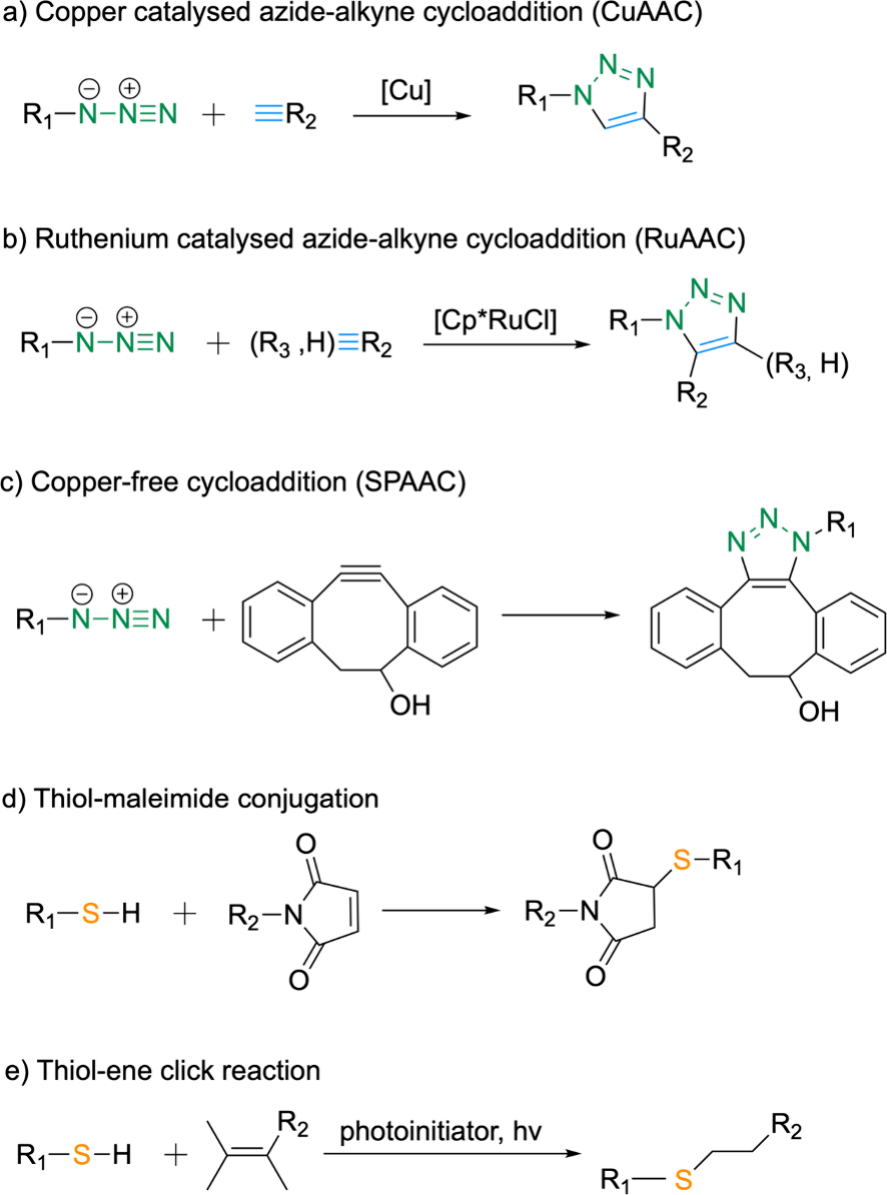
Examples of Common Click Reactions Involving the Conjugation of Two Larger Moieties^a^

a CuAAC (copper-catalyzed azide–alkyne cycloaddition) and RuAAC (ruthenium-catalyzed azide–alkyne cycloaddition) are metal-catalyzed reactions that yield two constitutional isomers. In contrast, SPAAC (strain-promoted azide–alkyne cycloaddition) involves the reaction of an azide group with a strained alkyne without the need for metal catalysis, utilizing the ring strain of cyclooctyne as the driving force. Unlike CuAAC, RuAAC, and SPAAC, which involve a triple bond, thiol-based reactions instead involve the addition of a sulfur atom to a double bond.

The copper-catalyzed azide–alkyne cycloaddition (CuAAC) is a prominent method due to its high efficiency and selectivity. This reaction involves the incorporation of azide and alkyne functional groups into the peptide and its counterpart, enabling the conjugation of the respective molecules to form a triazole linkage. First reported in 2002 by K. Barry Sharpless, Valery Fokin and Morten Meldal, this copper­(I)-catalyzed cycloaddition connects azides and terminal alkynes to produce 1,4-regioisomers of 1,2,3-triazoles as the sole products.^158,159^ The advancement of using a copper catalyst in aqueous environments improved upon the initial methodology introduced by Rolf Huisgen in the 1970s, which required elevated temperatures.^160^ While commercial sources of copper­(I), such as cuprous bromide or iodide, can be utilized, the reaction is notably more effective when conducted with a combination of copper­(II) and a reducing agent (e.g., sodium ascorbate) to generate Cu­(I) in situ. Given the instability of Cu­(I) in aqueous solvents, employing stabilizing ligands, such as tris­(benzyltriazolylmethyl)­amine (TBTA), enhances the reaction yield.^161^ The CuAAC reaction can be performed in a range of solvents, including mixtures of water and miscible organic solvents like alcohols, DMSO, DMF, and THF, while acetonitrile is typically avoided due to its strong coordinating ability toward Cu­(I). Additionally, the starting reagents can often be only partially soluble for the reaction to proceed successfully, and in many instances, the product can be isolated simply through filtration, eliminating the need for extensive purification steps.^162^

In contrast, the ruthenium-catalyzed 1,3-dipolar azide–alkyne cycloaddition (RuAAC) accommodates both terminal and internal alkynes, resulting in the formation of 1,5-disubstituted and 1,4,5-trisubstituted-1,2,3-triazoles. Unlike CuAAC, which is limited to terminal alkynes, RuAAC expands the scope by allowing both terminal and internal alkynes to participate in the reaction.^163^ Another notable development is the discovery of a broad-spectrum silver­(I)-catalyzed azide–alkyne cycloaddition reaction (Ag-AAC), which yields 1,4-triazoles. The mechanistic details of AgAAC closely resemble those of the copper­(I)-catalyzed process. It is important to note that silver­(I) salts alone are insufficient to facilitate cycloaddition; however, the presence of ligated Ag­(I) sources significantly enhances the effectiveness of the AgAAC reaction.^164^

Bioorthogonal chemistry plays a crucial role in conjugating proteins or peptides under biological conditions. One widely used bioorthogonal reaction is strain-promoted alkyne–azide cycloaddition (SPAAC) devoloped in the group of Carolyn R. Bertozzi.^165^ Unlike traditional azide–alkyne cycloaddition, SPAAC does not require a copper catalyst. Instead of activating the alkyne with Cu­(I), SPAAC introduces a strained cycloalkyne, such as difluorooctyne (DIFO), dibenzylcyclooctyne (DIBO), or biarylazacyclooctynone (BARAC).^166–168^ These strained cycloalkynes destabilize the alkyne, enhancing the reaction driving force and promoting the cycloalkyne to relieve its ring strain.^169^

The Staudinger reaction involves the reaction between a methyl ester phosphine and an azide, leading to the formation of an aza-ylide intermediate, which is subsequently captured to yield a stable covalent bond. This cross-linking chemistry, initially developed in the early 20th century by polymer chemist and Nobel Laureate Hermann Staudinger, has recently gained prominence in biological systems as a bioconjugation technique. It exhibits essential characteristics for bioorthogonal chemistry, such as biocompatibility, selectivity, and rapid, high-yield turnover, making it applicable across a diverse range of applications. This application in chemical biology is commonly referred to as Staudinger ligation.^170,171^

Thioether formation entails the reaction between a thiol group and an electrophile (such as a haloalkane or sulfonate ester) to generate a thioether linkage. A specific example of this process is thiol-maleimide conjugation, which occurs via a Michael addition mechanism between thiol (-SH) groups and maleimide to establish a stable thioether bond. Thiol-maleimide conjugation is widely used to attach chemical labels to peptides and proteins, including fluorescent dyes, polyethylene glycol (PEG), radiolabels, antibodies, and small molecules.^172^ The reaction offers several advantages, including rapid kinetics between maleimides and thiols, as well as a preference for neutral pH conditions. However, it is not without challenges; side reactions, such as thiazine rearrangement, can occur during thiol-maleimide conjugation.^173^ These side reactions are often attributed to the instability of the maleimide-cysteine conjugate. Notably, a significant increase in the rate of thiazine formation has been observed at basic pH values, indicating a base-dependent mechanism that involves nucleophilic attack of the succinimide by the N-terminal amine. Furthermore, substituting the amino acid adjacent to the N-terminal cysteine with various residues has resulted in the generation of thiazine impurities, albeit at different rates. Even when employing a maleimide linker designed for enhanced stability, considerable thiazine formation has been noted, suggesting the ubiquitous nature of this side reaction.^174^ The presence of thiazine impurities has been confirmed using various analytical techniques. Protonation of the N-terminal amino group in acidic conditions can inhibit the nucleophilic reaction and subsequent thiazine formation.^173^ However, performing conjugation under acidic conditions (around pH 5) necessitates subsequent purification and careful handling of peptide conjugates to prevent the loss of succinimidyl thioether. An alternative approach to mitigate thiazine formation involves the acetylation of the N-terminal cysteine. Given the widespread occurrence of the thiazine side reaction, it is advisible to avoid using N-terminal cysteine in peptide designs.

Thiol–ene click chemistry encompasses the reaction between a thiol group and an alkene group to form a thioether linkage.^175^ This method offers several advantages, including high yields, stereoselectivity, rapid reaction rates, and favorable thermodynamic profiles. The addition reactions generally proceed through catalyzed Michael additions or free-radical additions. In free-radical additions, various stimuli—such as light, heat, or radical initiators—can be employed to generate thiyl radical species. These radicals then react with the ene functional group via an anti-Markovnikov addition, resulting in the formation of a carbon-centered radical. Following this, a chain-transfer step occurs, wherein a hydrogen radical is removed from a thiol, allowing the process to continue through multiple propagation steps.^176^ This reaction is particularly valuable in radical-based photopolymerization because it can proceed quantitatively and rapidly through a straightforward mechanism under ambient atmospheric conditions. Depending on the thiol and ene functional groups involved, the carbon-centered radical is generated in this reaction.

Other photochemical cross-linking strategies involve the development of photoinducible reactions for conjugation, such as photoreactive small molecules like diazirines or benzophenones. Upon exposure to UV light, these molecules can be activated to form reactive intermediates that cross-link nearby biomolecules, allowing for spatiotemporally controlled modification of peptides and proteins. Additionally, photoreactive molecules can include amino acids, such as p-benzoylphenylalanine (pBPA), and photoreactive diazirine analogs of leucine and methionine.^177,178^ Upon exposure to ultraviolet light (UV), these molecules undergo activation, enabling them to covalently cross-link proteins within their native protein–protein interaction domains *in vivo*. This approach allows for the identification and characterization of both stable and transient protein interactions within cells, eliminating the necessity for traditional chemical cross-linkers and solvents that could interfere with the cellular biology under investigation in the experiment.

## 3 Topological Alterations in Peptidomimetics

Small molecules often face limitations in modulating or interfering with PPI. Their protein binding affinity tends to be lower than larger biological modulators such as antibodies, proteins, and peptides. In this context, short peptides and miniproteins emerge as promising candidates for rationalizing peptide-based drugs, offering higher target affinity and potentially reduced toxicity compared to small molecules.^179^

The biological activity and function of peptides depend highly on their ability to adopt specific shapes or conformations. Peptides with rigid conformations exhibit reduced flexibility, which enhances selectivity, improves stability against protease degradation, and lowers toxicity—key attributes that make them strong candidates for orally bioavailable peptide therapeutics. Synthetic approaches today aim to modify the topology of peptidomimetics, compelling them to assume specific conformations that stabilize the tertiary fold of proteomimetics or provide a more stable secondary structure under diverse conditions (Figure 8 and 9).

**Figure 8:**
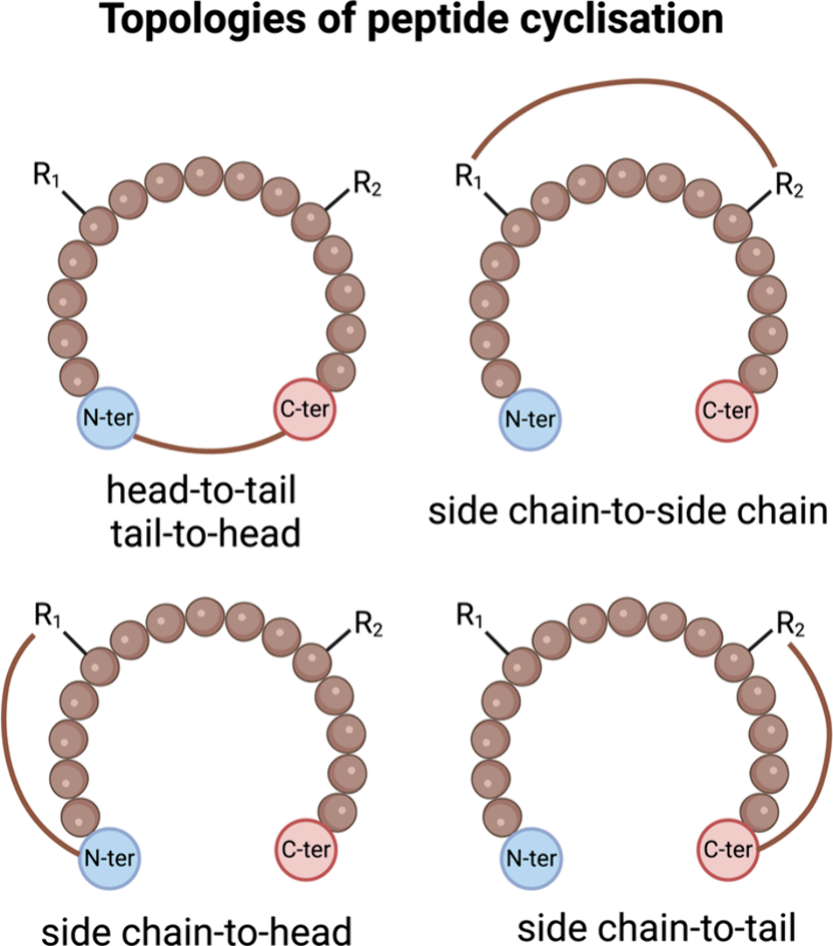
Types of topologies that can be achieved through peptide cyclization. A peptide ring can be formed via reactions between the N- and C-termini, between one terminus and a side chain, or between two side chains. In all cases, the primary objective is to stabilize the peptide in a specific conformation or to restrict the number of possible conformations, thereby reducing its flexibility. Created in BioRender.

**Figure 9:**
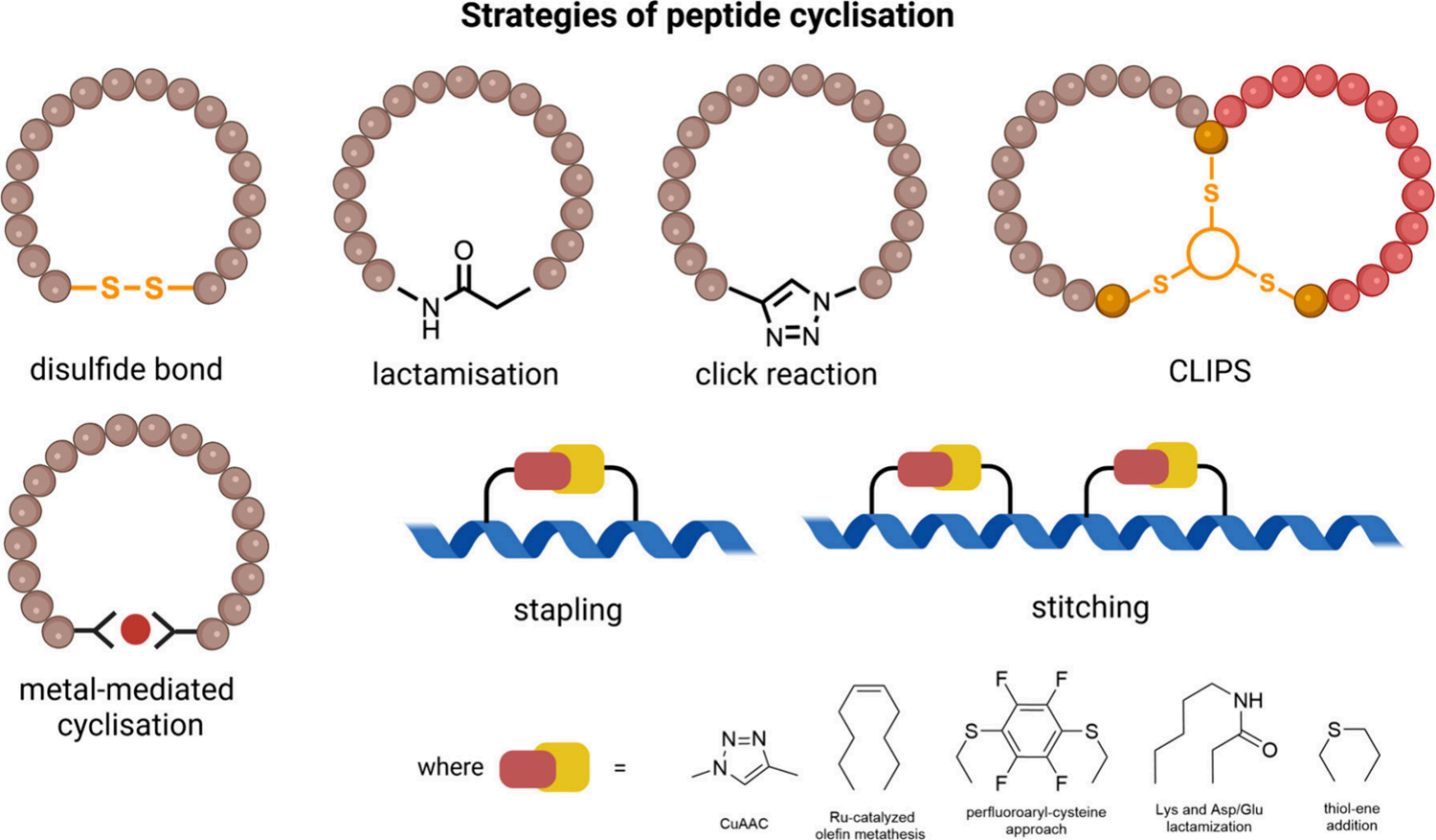
Common chemical strategies for peptide cyclization include disulfide bridge formation, lactamization, click reactions such as CuAAC, and metal-mediated cyclization where the metal is chelated by side chains or termini. Multiple cycles can also be introduced, as in the formation of a bicyclic structure using a scaffold, e.g., Chemical Linkage of Peptides onto Scaffolds (CLIPS). Cyclization methods also encompass stapling and stitching, which often involve click reactions or the formation of a carbon–carbon bridge. Created in BioRender.

In natural proteins, topology and flexibility are often altered by intramolecular cross-links, such as disulfide bridges strategically placed between distinct secondary structures to stabilize the tertiary fold. Other methods utilized other natural amino acids to form metal-mediated bonds, or lactam groups.^180–182^ It is important to note that the same approaches can be employed to stabilize structures beyond helices or produce macrocyclic structures. For instance, naturally occurring Cys-Cys bonds are found in β-sheets (e.g., defensins) and are harnessed to stabilize β-hairpins and dimers of β-sheets.^183^ Similarly, click chemistry stapling techniques can confer stability to β-turns and β-hairpins.^184^ Macrocyclic peptides have demonstrated suitability as pharmaceuticals and recent advances have moved beyond mimicking natural cyclic peptides.^185^ Medium-size peptides are emerging as molecules bridging the gap between small molecules and biologics, showing potential to target previously challenging proteins to interact with using small molecules.^186^

In addition to the macrocyclization mentioned for stabilizing peptide structures, lasso peptides represent another fascinating class of naturally occurring, highly stable, and structurally unique peptides. Synthesized ribosomally by microorganisms, these peptides feature a knot-like structure in which the peptide backbone forms a loop covalently threaded through an amino acid side chain, resulting in a “lasso” shape.^187^ This configuration imparts remarkable stability, protecting the peptide from degradation and enhancing its bioactivity. Lasso peptides have demonstrated a wide range of biological activities, particularly in antimicrobial defense. Their rigidity, due to the knot formation, makes them ideal candidates for pharmaceutical development. Indeed, lasso peptides are increasingly being engineered for therapeutic use, showing promise as antimicrobials or antitumor agents.^188,189^ Their ability to bind tightly and selectively to target proteins or enzymes opens new avenues for drug discovery, particularly for challenging biological pathways that are difficult to target with conventional small molecules. For readers interested in learning more about lasso peptides, we encourage starting with excellent papers authored by the research groups led by Mitchell, Marahiel, and Swanson.^190–193^

### 3.1 Mimicry of Cys-Cys Natural Cycles

Within the field of peptidomimetics, disulfide bonds serve as constrained structural elements frequently employed to generate macrocycles. This practice helps immobilize the peptide in its bioactive conformation, thereby enhancing the pharmacological properties of peptides. Despite their predominant role in these applications, disulfide bonds exhibit multifaceted functions, participating in oxidative folding and other biological processes.^194^ Due to their involvement in redox reactions and biological processes, disulfide bridges are not an ideal choice for generating stable cyclic peptides, and alternative strategies have therefore been explored.

One approach to mimicking or replacing disulfide bonds is the use of bis-electrophilic linkers. These linkers come in various forms, including those based on alkylation, acylation, Michael addition, nucleophilic aromatic substitution, and metal-mediated coupling—all of which exploit the unique nucleophilicity of sulfur.^195^ Linkers with two identical electrophilic groups are primarily limited to intramolecular processes such as macrocyclization, stapling, and disulfide rebridging, or to the formation of homodimers (Figure 10a-c). To enable selective cross-conjugation, nonsymmetrical linkers with sufficiently different reaction rates between their electrophilic groups are required. Maleimide-succinimidyl esters (Figure 10d and e) are among the most widely used heterobifunctional cross-linkers in bioconjugation. However, maleimide conjugates can present stability issues in biological systems, and the activated ester in (Figure 10d) is rapidly hydrolyzed in alkaline aqueous media, reacting with both thiols and amines. This issue can be mitigated by replacing the ester with an azide (Figure 10e) to enable bioorthogonal reactions, but this modification prevents the use of natural amino acids as conjugation partners.

**Figure 10:**
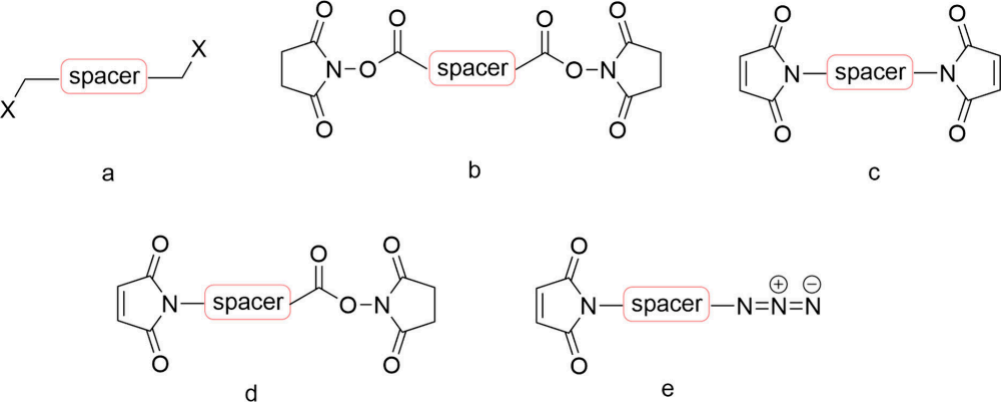
Bis-electrophilic linkers for bioconjugation and mimicking disulfide bonds. Top row: homobifunctional linkers. Bottom row: heterobifunctional linkers.

A related study explores the use of 1,4-dinitroimidazoles for macrocycle formation. These compounds function as highly efficient bifunctional bioconjugation reagents, reacting with cysteine side chains under aqueous acidic and neutral conditions via a *cine*-substitution mechanism to form stable products.^196^ In these conditions, 1,4-dinitroimidazoles react selectively with cysteine. However, in organic solvent and with base, 1,4-dinitroimidazoles can also react with lysine through a ring-opening and ring-closing mechanism (Scheme 8). By exploiting their ability to react with both cysteine and lysine via distinct mechanisms, these reagents enable the formation of bioconjugates with superior chemoselectivity and stability compared to conventional maleimide–thiol conjugates.

**Scheme 8:**
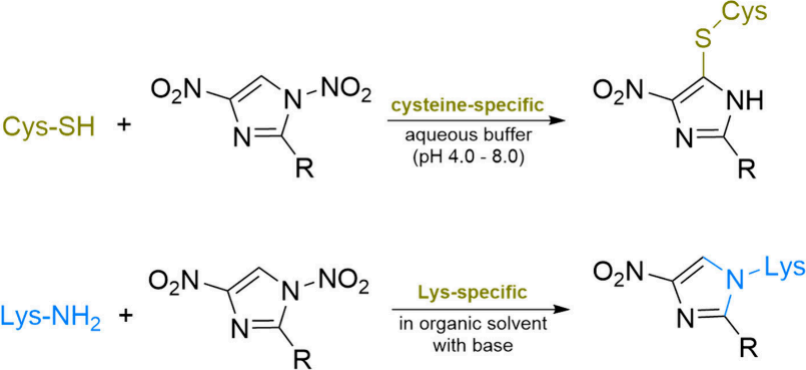
1,4-Dinitroimidazoles React with Cysteine and Lysine via Two Distinct Mechanisms, Exhibiting Differential Chemoselectivity Depending on Whether the Reaction Occurs in Aqueous or Organic Solvents

The research groups of Wade and Hossain developed strategies to replace disulfide bonds in insulin. Mature insulin is stabilized by three disulfide bonds: two linking the A and B chains and one within the A chain. Although the synthesis of the two chains was achieved many years ago, correctly forming the disulfide bonds to link and stabilize them remains a significant challenge. Many approaches rely on orthogonal cysteine protection and regioselective disulfide bond formation, while others focus on directly mimicking the disulfide bridge. The groups employed cystathionine to replace the A6-A11 intrachain disulfide bond, leading to enhanced thermal stability.^197^ This approach involved substituting a disulfide bridge with a thioether linkage, with cystathionine being generated in situ from cysteine using orthogonal protection strategies. Further details on the mechanism are illustrated in Scheme 9.

**Scheme 9:**
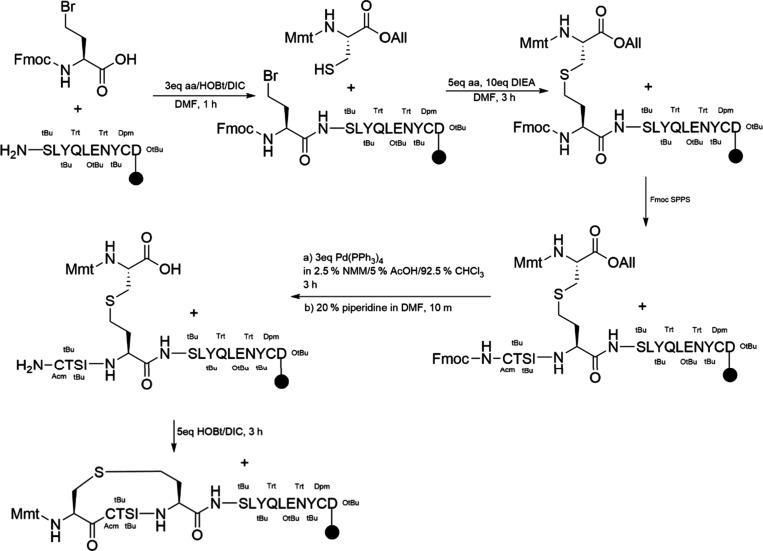
Scheme Illustrating the Use of Cystathionine Bridges to Replace Intrachain Disulfide Bonds^a^

a This strategy begins by substituting a disulfide bond with a thioether linkage, followed by peptide elongation through solid-phase synthesis. The cystathionine bridge is generated in situ from cysteine residues using orthogonal protection strategies. After cystathionine formation, peptide chain elongation proceeds to complete the full sequence (not shown). The scheme also details the reagents and conditions used up to the formation of the cystathionine linkage.

Hirudin is a 65-amino acid peptide that contains three disulfide bridges. In a study, selenium was used as a substitute for sulfur in cysteine to investigate the effects of diselenide bridges on folding, structure, and activity, both at native and non-native positions.^198^ Three designed analogues incorporated diselenide bonds at the native cross-links (6–14, 16–28, and 22–39), while a fourth analogue introduced a diselenide bridge at a non-native position (6–16), based on the proposed role of this non-native disulfide bond in the early stages of hirudin folding. Overall, the results indicate that replacing native disulfide bonds with diselenide cross-links enhances folding efficiency toward the native state, significantly reducing the formation of nonproductive intermediates. Notably, even the non-native diselenide-containing analogue (6–16) exhibited a similar improvement in folding efficiency.

Macrolactamization is a widely utilized strategy to build a bridge due to several key advantages, including its mild reaction conditions, which are compatible with sensitive functional groups, high chemoselectivity for desired cyclic structures, and versatility stemming from the broad availability of starting materials such as amines and carbonyl-containing compounds. Moreover, its biological relevance is underscored by the prevalence of lactam rings in natural products and bioactive molecules. Despite these merits, macrolactamization is limited by challenges such as steric hindrance, substrate incompatibility due to reactivity issues, and potential polymerization side reactions caused by competition between inter- and intramolecular processes. The incorporation of turn-inducing elements (TIEs), such as proline, has been shown to effectively address these challenges, facilitating the synthesis of a miniprotein with a small β-sheet structure.^199^ A critical loop connects the two β-strands, promoting protein–protein interactions (PPI). This design draws inspiration from the VP3VR-VIII region of the adeno-associated virus (AAV) capsid protein.

Additionally, strategies involving hydrocarbon bridges to “staple” peptides across side chains or hydrogen bond surrogates in the backbone have proven effective in producing biologically functional molecules stabilizing the helical structure.^200^ Stapling techniques employing non-natural elements enhance protease resistance and potency both in vitro and in vivo.

Another general cyclization method has been developed, drawing inspiration from nonribosomal peptide synthetases (NRPSs).^201^ Natural cyclic peptides, such as rufomycin and cyclosporin A, are biosynthesized by NRPSs, which possess the ability to incorporate unnatural amino acids and introduce diverse modifications, such as *N*-methylation, epimerization, and oxidation, during peptide synthesis. The total synthesis of nonribosomal cyclic peptides (NRcPs) traditionally relies on standard coupling reagents. However, this approach is often labor-intensive, requiring extensive use of protecting groups, prolonged reaction times, and yielding side reactions like epimerization and dimerization, which lower the overall efficiency. The newly developed method, inspired by the biosynthetic cyclization processes of NRPSs, enables the production of macrocycles with remarkable speed (within minutes), high selectivity, and excellent yield. This general approach to NRcP synthesis and macrocyclization is effective regardless of sequence or ring size. The process involves the synthesis of a linear peptide hydrazide via standard solid-phase peptide synthesis. After complete deprotection, the hydrazide is oxidized to an azide, facilitating tail-to-head cyclization. The study also highlights the critical role of pH and solvent choice. Oxidation of the hydrazide is most efficient in water under acidic conditions, whereas the actual cyclization step proceeds optimally in an organic solvent at neutral pH. This biphasic method achieves rapid cyclization in just a few minutes, offering a highly efficient and versatile strategy for cyclic peptide synthesis.

### 3.2 Metal-Mediated Bridging Strategies

Natural cyclic peptides, exemplified by valinomycin and various potent ionophores with metal-mediated side-chain links within their structures, have inspired scientists to leverage metals to enclose macrocycle peptides. Metals play a dual role, not only facilitating cyclization but also influencing the secondary structure of peptides.

The initial applications of metal-mediated cyclization involved the dimerization of peptide methyl esters and the binding of carbonyl and amide groups at peptide termini.^202^

Metal–ligand interactions play a pivotal role in directing peptide structural control by stabilizing helices and facilitating the formation of coiled coils and multihelical complexes. Peptides that are shorter than 15 residues typically struggle to adopt α-helical structures. However, the introduction of metal ions can assist in helix formation by creating bridges that enhance structural stability. This metal-assisted stabilization technique finds applications in investigating protein folding, designing peptidomimetics, and developing inhibitors.

Transition metals, notably Ni^2+^, Zn^2+^, Cd^2+^, and Cu^2+^, are frequently employed due to their ability to form stable complexes with side chains of histidines, cysteines, or non-natural amino acids featuring two carboxylic groups. The formation of these complexes leads to the generation of macrocycles, effectively stabilizing the peptide backbone.^203^ It is of significance to note that alkali metals (such as Li^+^, Na^+^, and K^+^) lack the ability to chelate side chains on peptides. However, they exhibit the capability to transform random coils into rigid helices. This transformation is particularly significant for coiled-coil structures, which are assemblies formed by helical sequences characterized by amino acid positions typically denoted as a-g.^204,205^ Coiled coils are formed by hydrophobic interactions, primarily involving residues located at the a and d positions. Additionally, the e and g positions play a crucial role in stabilizing these structures through ionic interactions, specifically where the g position of one helix interacts with the e position of another. Metal ions can be strategically employed to facilitate a controlled folding transition of coiled coils. By incorporating high-affinity metal binding sites at the e and g positions, one can effectively stabilize the coiled-coil formation. When both binding sites contain negatively charged side chains, they repel each other in the absence of the preferred metal ion, thus preventing assembly. The introduction of metal ions mitigates this repulsion and promotes the correct alignment and stabilization of the coiled-coil structure. Moreover, zinc ions have been utilized to mediate bridging and create a 16-helix arrangement with four copies of cytochrome cb562 (cyt cb562). Each cyt cb562 represents a 4-helix bundle heme-containing protein. In this context, Zn­(II) coordinates the di-His motifs on the surface of cyt cb562 (PDB: 2QLA). The coordination of Zn-His plays a crucial role in protein multimerization, as evidenced by the dissolution of aggregates upon adjusting the pH below 6 and treating with EDTA.^206^

Several studies have employed metals to facilitate peptide cyclization. An early example is the use of silver ions, which enable the cyclization of unprotected or minimally protected peptides. All Ag^+^-assisted cyclizations of minimally protected peptides were conducted in aqueous acetate-buffered solutions at pH 5–6, for two main reasons.^207^ First, the affinity of the Ag^+^ ion follows the order S ≫ *N* > O. Thus, coordination of one or more Ag^+^ ions between the nitrogen atom of the N-terminal amino group and the sulfur atom of the C-terminal thioester could promote the formation of the desired cyclic intermediate. Second, under these mildly acidic aqueous conditions, Ag^+^-mediated hydrolysis proceeds slowly while selectivity for aminolysis remains high. This reaction exhibits chemoselectivity toward the formation of lactams and lactones, typically requiring two equivalents or more of silver ions and a reaction time of approximately 2 h to achieve complete cyclization.

Another strategy involves exploiting the strong affinity between nickel ions and histidine residues. Positioning three histidine residues at each terminus of a peptide with low intrinsic propensity for independent secondary structure formation can induce an ordered conformation in the presence of nickel. Structural studies conducted via NMR further demonstrate that the insertion of a single histidine residue at each end of short bioactive peptides promotes a more compact and predictable folding pattern, without significantly altering the peptide backbone.^208^

The metal ion in these reactions is not merely part of the cyclization process but also acts as a type of catalyst. For example, natural peptides composed of five or seven residues have been synthesized and cyclized using 3-(diethoxyphosphoryloxy)-1,2,3-benzotriazin-4­(3H)-one (DEPBT) as a coupling reagent in solution, with the process mediated by different metal ions.^203^ Although the linear peptides lack side chains capable of strong metal complexation, metal ions such as Fe^2+^, Ni^2+^, Zn^2+^, and Cr^3+^ were found to strongly coordinate with the carboxyl groups. In contrast, alkali metal ions such as Li^+^, Na^+^, K^+^, Rb^+^, and Cs^+^ do not form strong complexes like transition metals, but they can coordinate to the oxygen atoms of carbonyl and amide groups near the C-terminus with low affinity. This coordination promotes the formation of a turn structure, as demonstrated by CD spectroscopy studies. The resulting turn brings the N- and C-termini of the linear peptide into proximity, thereby enhancing the efficiency and yield of cyclization.

The groups of Pentelute and Buchwald have reported that palladium­(II) complexes can be employed for efficient and highly selective cysteine conjugation reactions, which proceed rapidly and under a broad range of biocompatible conditions.^209^ The straightforward synthesis of these palladium reagents from a variety of readily available aryl halides and trifluoromethanesulfonate precursors makes the method highly practical, enabling access to a wide structural space for peptide and protein modifications. Palladium reagents bearing two electrophilic metal centers were effectively utilized to cross-link two cysteine residues within a peptide chain, thus allowing the generation of stapled peptides featuring various aryl linkers. Notably, performing the reaction at a peptide concentration of 10 mM in a 1:1 (v/v) acetonitrile/water mixture at pH 7.5, with a 2-fold excess of the bis-palladium complex 2A, led to the quantitative formation of the desired stapled peptide within 10 min. The resulting aryl bioconjugates demonstrated high stability against acids, bases, oxidants, and external thiol nucleophiles. These palladium complexes show considerable promise as practical benchtop reagents for diverse bioconjugation applications.

Similarly, the same groups demonstrated that, in the presence of a biarylphosphine-supported palladium­(II)–aryl complex and a weak base (sodium phenoxide, p*K*_a_ = 10), lysine amino groups in unprotected peptides underwent C–N bond formation at room temperature.^210^ This reaction and the developed protocol enable the formation of N–aryl conjugates, which exhibit greater stability compared to their corresponding S–aryl counterparts. This approach proved effective for the conjugation of a variety of organic compounds, including peptides, which were successfully cyclized.

### 3.3 Stapling Techniques

Macrocycles can be formed by creating a bridge between residues aligned on the same face of the helix, typically at positions i, i+4, i+7, and i+11, with i+4 and i+7 being the most common. Early stapling strategies involve using natural amino acids for side chain-to-side chain cross-linking. Examples include the use of lactam between Lys and Glu/Asp residues, thioether between two Cys residues, His-His via metal chelates, and various other methods involving proteinogenic amino acids and synthetic approaches.^211^ Utilizing natural amino acids as anchoring points necessitates either selective orthogonal protection or the replacement of identical amino acids with different ones within the peptide sequence, imposing limitations on this peptide stapling approach.

One of the well-established peptide stapling strategies that overcome these limitations involves the use of unnatural amino acids, specifically through hydrocarbon stapling. This technique employs Grubbs catalysts to link the side chains of two non-natural amino acids in the solid phase at positions i, i+4, or i, i+7. The Grubbs catalyst, [(PCy_3_)_2_Cl_2_Ru = CHPh], plays a pivotal role in initiating the formation of a carbon–carbon bridge, connecting specific α,α-disubstituted amino acids with olefinic side chains through a ring-closing metathesis (RCM) reaction.^212^ The hydrocarbon bridge connects at two locations along a synthetic peptide backbone and stabilizes the α-helical arrangement forming a macrocycle with increased stability and hydrophobicity.

Stapling also emerges as a method to constrain and stabilize non-natural peptide foldamers into helical-mimicking conformations. In an initial study, the Hoveyda-Grubbs generation II catalyst was employed to staple β-peptides.^213^ These stapled peptides exhibited helicity in a pure phosphate buffer and various solvents, including TFE, methanol, and a combination of acetonitrile and buffer. An extensively utilized strategy for chemical ligation and peptide stapling involves the Cu­(I)-catalyzed azide–alkyne 1,3-dipolar Huisgen cycloaddition, commonly known as the CuAAC click reaction or the strain-promoted azide–alkyne cycloaddition (SPAAC) (see Section 2.8.1).^214^

In general, stapling methods involving natural amino acids, Grubbs catalysts, or click chemistry constitute one-component stapling techniques, allowing the direct coupling of complementary side-chain groups. In contrast, two-component stapling employs a bifunctional linker compound that reacts with two complementary non-native amino acids in the peptide to form a staple.^215^ This stapling technique involves reacting linear i,i+7 diazido peptides (i.e., containing two azido amino acids that are seven residues apart) with dialkynyl stapling linkers under Cu­(I) catalysis. As this reaction produces peptides bearing a bis-triazole linkage, this process is called double-click or two-component stapling.^216^ The primary advantage of two-component stapling lies in the ability to introduce more diverse staple linkages without the need for synthesizing complex unnatural amino acids. However, the more intricate reaction pathway in two-component stapling may lead to the generation of more byproducts compared to one-component stapling. One example of a competing path involves coupling two linker moieties to a single peptide, one at each non-native amino acid.

Most two-component stapling techniques are adaptations of their one-component stapling counterparts, with bis-lactamization being an example.^217^ The use of natural amino acids simplifies the synthesis of linear peptides due to their cost-effectiveness and availability, minimizing alterations to the wild-type peptide sequence and potentially avoiding negative impacts on binding affinity. However, challenges may arise regarding orthogonality and chemoselectivity. Many two-component stapling strategies that utilize natural amino acids primarily focus on lysine and cysteine, with limited applicability to tryptophan. In contrast, employing unnatural amino acids for peptide stapling requires either procuring or synthesizing these nonproteinogenic amino acids, which can be both costly and time-consuming. Nonetheless, this approach offers excellent orthogonality and allows for a variety of staple compositions.^218^

Another stapling method was used by the Pentelute group who identified an efficient transformation process involving perfluoroaromatic molecules and a cysteine thiolate, leading to arylation at room temperature.^219^ This method allows for the selective modification of cysteine residues in unprotected peptides, enabling the incorporation of rigid perfluoroaromatic staples. When applied to a peptide sequence designed to interact with the C-terminal domain of the HIV-1 capsid assembly polyprotein (C-CA), this stapling modification resulted in improved binding affinity, cell permeability, and proteolytic stability compared to its unstapled counterpart. Importantly, the chemical stability of the resulting staples facilitated their use in the native chemical ligation-mediated synthesis of a small protein capable of binding to the human epidermal growth factor receptor 2 (HER2). The same research group reported a mild and efficient method for synthesizing macrocyclic peptides via nitrogen arylation from unprotected precursors. They explored various electrophiles and lysine-based nucleophiles, successfully generating high-yield products in a macrocyclization scan that included 14 different variants. The nitrogen-linked aryl products demonstrated greater stability against base and oxidation than thiol-arylated counterparts, highlighting the advantages of this methodology.^220^ Notably, when this N-aryl macrocyclization was applied to a p53 peptide inhibitor of MDM2, it led to the discovery of a nanomolar binder with improved proteolytic stability and cell permeability.^220^

### 3.4 Bicyclic Peptides and CLIPS Cyclization Technology

Peptides that contain two macrocyclic structures are commonly referred to as bicyclic peptides. This classification includes peptides with two loops formed by a scaffold anchored at three points within the peptide sequence. Additionally, macrocyclic peptides featuring an internal bridge are also categorized as bicyclic peptides, a structural motif frequently observed in nature. Some definitions may also encompass peptides with double-stapled or double-macrocyclic configurations. Research efforts continue to explore various bicyclic topologies, aiming to establish novel synthetic pathways.^221–226^

Bicyclic peptides have gained prominence as a significant subset within the constrained peptide family and are expected to possess substantial therapeutic potential, as evidenced by the growing interest reflected in scientific literature.^225,227–229^ Compared to monocyclic peptides, bicyclization offers several advantages, including enhanced structural rigidity, improved metabolic stability, cell permeability and increased target affinity.^230^ Their binding characteristics, similar to those of antibodies, enable them to effectively disrupt protein–protein interactions. With two macrocyclic structures, these peptides can engage with a single target structure or simultaneously bind to two different targets. As a result, bicyclic peptides are frequently designed for applications in antimicrobial or anticancer therapies, making them a compelling area of research for next-generation pharmaceuticals.^231,232^ This growing interest is underscored by the emergence of several companies, such as Bicycle Therapeutics and Pepscan, that focus primarily on developing bicyclic peptides.

Chemical Linkage of Peptides onto Scaffolds (CLIPS) is the methodology to produce bicycles wherein peptides are cyclized and tethered onto a central scaffold to impart rigidity and stability. The central scaffold may consist of organic molecules, dendrimers, or other polymeric structures. By attaching peptides at multiple points to the scaffold, a precisely defined three-dimensional structure is created. The benefits of CLIPS include precise control over peptide conformation and spatial arrangement, the ability to present multiple peptides in a defined orientation, and enhanced binding properties due to the stable and rigid conformation. CLIPS finds application in the development of vaccines and immunogens by presenting epitopes in a native-like conformation.^233^

1,3,5-tris­(bromomethyl)­benzene (TBMB) is widely utilized as a reagent for synthesizing bicyclic peptides through cysteine alkylation. Its application in conjunction with phage-displayed proteins has represented a significant stride in the development of genetically encoded bicyclic peptide libraries. Integration of TBMB with the phage display platform enables the rapid identification of bioactive bicyclic peptides through iterative selections, presenting a molecularly lighter alternative to antibodies and other binding proteins.^234,235^ However, excessive TBMB usage may induce nonspecific modification of linear peptides. To enhance this technique, additional reagents with similar symmetry and thiol-reactivity, 1,3,5-triacryloyl-1,3,5-triazinane (TATA), 1,3,5-tris­(bromomethyl)­benzene (TBMB), and N,N’,N’’-(benzene-1,3,5-triyl)­tris­(2-bromoacetamide) (TBAB), have been developed (Figure 11).^226,234^

**Figure 11:**
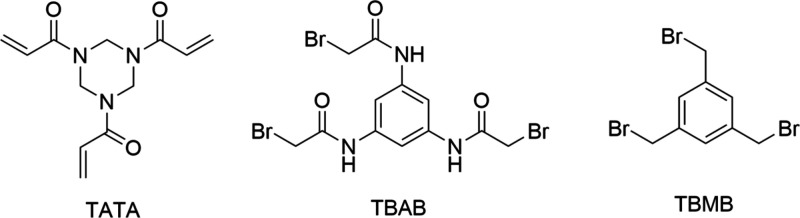
Three common organic linkers applied for the cyclization of bicyclic peptides.

In recent years, innovative approaches have been developed for synthesizing bicyclic peptides utilizing the triple cysteine motif. Much like TBMB alkylation, these strategies often capitalize on the unique nucleophilicity of cysteine residues. Pentelute and colleagues previously established a peptide stapling method using perfluoroaryl linkers.^219^ To adapt this technique for connecting three cysteine residues, they initially employed an excess of decafluorobiphenyl (DFBP) to monosubstitute each cysteine side chain. Following this step, benzene-1,3,5-trithiol (BTT) was introduced to the modified peptide, enabling bicyclization through a second nucleophilic aromatic substitution.^236^ Double-stapled peptides can be synthesized from four cysteines if two are orthogonally protected with StBu.

To expand beyond cysteine modifications, Chen and colleagues developed a reaction that utilizes lysine and arginine residues for cyclization.^237^ This method, based on a stapling strategy that employs formaldehyde to link amino acids, allows for the creation of multicyclic peptide topologies. By introducing formaldehyde along with amine and guanidine, they successfully achieved the cyclization of peptides containing two lysines and one arginine residue. Alternatively, by substituting the arginine side chain with a lysine residue and using guanidine as a reagent, a connection between three lysines could be formed. However, the inclusion of basic amino acids in this bicyclization limits the sequence diversity of potential bicyclic peptides that can be constructed using canonical amino acids.

As in macrocyclization, metals can also assist in the formation of bicyclic structures. For instance, in the case of a triple-cysteine peptide, Stauber et al. used Au^3+^ and complexes such as the *tert*-butyl substituted aminophosphine-supported Au­(III) complex, known as the (P,N) supported Au­(III) complex.^238^ The trimetallic (P,N) supported Au­(III) complex, denoted [3]^3+^, which features three metasubstituted (P,N)­Au­(C_6_H_4_)Cl fragments surrounding a central aryl anchor, was synthesized. Treatment of 1,3,5-tris­(4-iodophenyl)­benzene with the (P,N) supported Au­(III) complex in the presence of AgSbF_6_ resulted in clean conversion to [3]^3+^ at room temperature. Efficient cyclization of the model linear tricysteine peptide, H_2_N–GCAENCAFGCA–CONH_2_, via its three cysteine thiols was achieved by treating the peptide with complex [3]^3+^ in a TRIS buffer and MeCN solvent mixture.

Bi­(III) was introduced to overcome the limitations associated with TATA, TBAB, and TBMB. While TBMB binds irreversibly, potentially modifying other reactive peptide residues even when used in slight excess, scaffolds such as TATA and TBAB were specifically developed to stabilize peptide conformations by promoting hydrogen bond networks. However, all conventional scaffolds contain flexible bonds, which ultimately limit their ability to fully rigidify peptide bicycles. Bi­(III) is nontoxic, selective, stable, and rigid, and it effectively interacts with cysteine residues in peptides and proteins. Peptide–bismuth bicycles form instantaneously at physiological pH, are stable in aqueous solutions for extended periods, and exhibit significantly higher resistance to proteolysis compared to their linear precursors.^239^ These bicyclic peptides show up to 130 times greater activity and 19 times more proteolytic stability than their linear analogs without bismuth. Additionally, they target proteases from Zika and West Nile viruses, unveiling a new lead compound with inhibition constants of 23 and 150 nM, respectively.

Metal-mediated S-arylation approaches require only one reaction to bicyclize a.[ ^52^,^53^ ] Stauber et al. developed a number of Au­(III)-complexes not only limited to bicyclization, but also suitable for mediating multisite bioconjugation and peptide stapling.[^52^] Bicyclic peptides were generated in a mixture of neutral buffer and acetonitrile, whereby the central scaffold of various Au­(III) complexes was transferred onto the three cysteines of a linear peptide (Scheme 3).[^52^] Mudd et al. later expanded this work by introducing further Au­(III) complexes that contained smaller scaffolds (Scheme 3).[^54^] Previously, Chen and co-workers had found an alternative pathway to effectively create similar bicycles using Pd-catalyzed S-arylation with triiodoarenes (Scheme 3, Figure 4b).[^53^]

Recent strides have been taken in designing intramolecular bicyclization reactions, departing from traditional methodologies employing external reagents on assembled linear peptides. This innovative approach involves integrating a reactive handle into the linear peptide chain. Reymond and collaborators exemplified this by coupling 3,5-bis­(chloromethyl)-4-methylbenzoic acid to the peptide N-terminus, leading to the subsequent formation of two thioether linkages with cysteines.^240^ Through this strategy, they synthesized bicyclic antimicrobial peptides effective against multidrug-resistant strains of *Acinetobacter baumannii* and *Pseudomonas aeruginosa*. Another strategy entails synthesizing an amino acid with two carboxylic acids during solid-phase peptide synthesis. A photoreaction was employed to introduce two 3-mercaptopropionic acid molecules to propargylglycine, enabling selective internal amide couplings following orthogonal deprotection of two amines. Notably, a dual-targeted, α-helical bicycle synthesized via this method exhibited potential as a cytotoxin for cancer treatment.

Natural bicyclic peptides frequently feature internal cross-links, making this topology a significant target for chemical synthesis. Recent examples include various bridges within macrocyclic peptides, such as FF, FY, and YY-like biaryl linkages formed in cyclic peptides. The synthesis of these structures generally requires microwave-assisted Suzuki-Miyaura cross-coupling conditions. Teixidó and collaborators utilized this cross-coupling technique to connect two tryptophan residues in cyclic peptides, enabling homocouplings at different positions using various bromotryptophan derivatives.^241^

Bicyclic peptides exhibit increased stability and improved cell permeability, making them promising candidates for drug development. The Grossman group illustrated that the formation of the bicyclic structure can enhance the β-sheet character of the macrocycle, thereby improving its ability to penetrate cells. Their research focused on identifying a novel target for β-catenin, which is a central hub for intracellular interactions within the Wnt signaling pathway. They reported the creation of a library of β-sheet-mimicking bicyclic peptides that specifically target β-catenin, compete with transcription factors for binding, and inhibit Wnt signaling in cellular contexts.^242^

## 4 Advancing Synthetic Chemistry through the Integration of Biotechnology

Advancing synthetic chemistry through the integration of biotechnology involves harnessing the power of biological systems and techniques to enhance traditional chemical synthesis methods. This interdisciplinary approach combines principles from chemistry, biology, and engineering to develop innovative strategies for creating complex molecules with improved efficiency, selectivity, and sustainability. By leveraging the capabilities of biological systems, such as enzymes, microorganisms, and genetic engineering tools, researchers can overcome challenges in traditional synthetic chemistry and unlock new opportunities for drug discovery, and chemical manufacturing.

One powerful technique within this integration is directed evolution, a method that exemplifies how biotechnology can be used to accelerate the development of novel chemical compounds. Directed evolution enables the generation of diverse peptide sequences with desired properties through iterative rounds of mutagenesis, selection, and amplification.^243,244^ This approach starts with a known peptide sequence or scaffold, which is subjected to random or targeted mutations using techniques such as error-prone PCR or DNA shuffling. The resulting library of peptide variants is screened or selected for specific activities or properties of interest. Selected peptides are then subjected to further rounds of mutagenesis and selection to optimize their performance.^245^ Direct evolution allows for the creation of peptide sequences that may not exist in nature, providing access to a vast sequence space beyond what is found in biological sources.

Libraries derived from biological sources are often synthesized and screened using bacteriophages (phage display) or cell-free technologies such as mRNA display (Figure 12).

**Figure 12:**
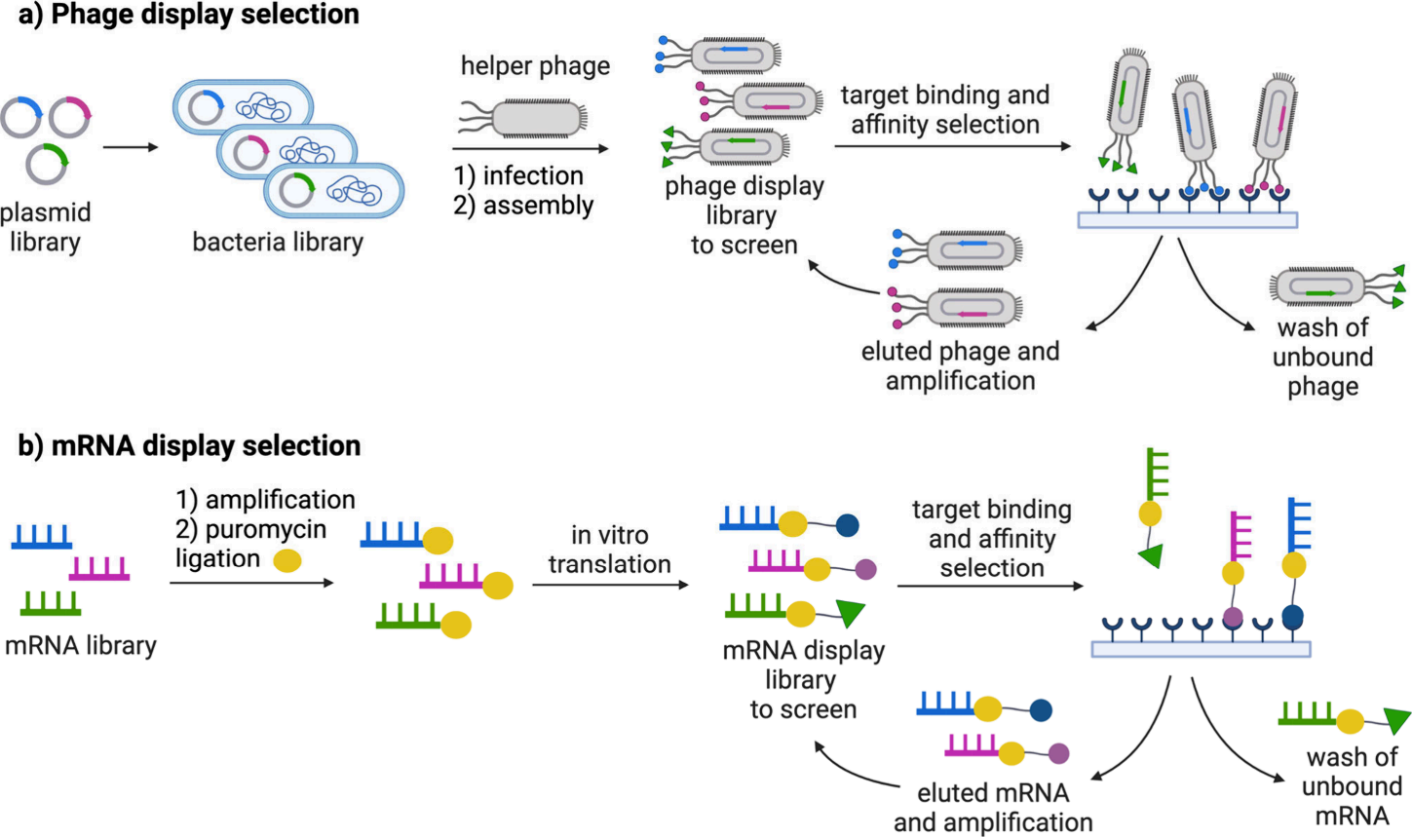
Drug discovery using display techniques. (a) In phage display, peptides or proteins for screening are expressed and displayed on the surface of bacteriophages. After interaction with the target, peptides or proteins with low affinity are washed away, while those with higher affinity are retained and subjected to further rounds of screening. Iterative cycles of screening progressively enrich for drugs with higher target affinity. (b) In mRNA display, a similar iterative screening process is employed, but potential drug candidates are covalently linked to the mRNA from which they were synthesized. Created in BioRender.

Phage display is a powerful technique utilized to investigate interactions among proteins, peptides, and DNA This method leverages bacteriophages—viruses that specifically infect bacteria—to associate proteins with their corresponding genetic sequences.^246^ In phage display, a gene encoding the protein of interest is inserted into a gene responsible for a phage coat protein, resulting in the phage displaying the protein on its surface while the genetic information is contained within.^247^ This arrangement creates a direct linkage between the genotype and phenotype. The displayed proteins can then be screened for interactions with other proteins, peptides, or DNA sequences, facilitating the identification of binding partners. As a result, extensive libraries of proteins can be screened and selectively amplified through a process called in vitro selection, which emulates the principles of natural selection.

mRNA display is an innovative technique utilized for the in vitro selection and evolution of proteins and peptides, enabling the generation of molecules with high affinity for specific targets.^248,249^ This process involves the creation of translated peptides or proteins that are linked to their corresponding mRNA progenitors through a puromycin linkage. During the selection phase, these fusion molecules interact with an immobilized target via affinity chromatography. Molecules exhibiting strong binding affinities are then reverse transcribed into complementary DNA (cDNA), followed by amplification of their sequences using polymerase chain reaction (PCR). This results in the generation of a nucleotide sequence that encodes a peptide with a high affinity for the target. Puromycin functions as an analogue of the 3′ end of tyrosyl-tRNA, mimicking both adenosine and tyrosine. In mRNA display, all mRNA templates have puromycin linked to their 3′ ends. As translation occurs, the ribosome traverses the mRNA template, and upon reaching the 3′ end, the attached puromycin enters the ribosome’s A site and is incorporated into the growing peptide chain. This incorporation leads to the release of the mRNA-polypeptide fusion from the ribosome. Unlike the cleavable ester bond found in tyrosyl-tRNA, puromycin possesses a nonhydrolyzable amide bond, which disrupts translation and causes the premature release of the translation products.^250^

Not only linear peptides but macrocyclic peptides can be produced through biological methods, with libraries generated using various techniques:

- disulfide bridge formation: cysteine residues can be randomly incorporated into sequences displayed on the surface of a phage, allowing the formation of disulfide bridges. A notable variant of this approach is the phage display combined with CLIPS cyclization technology.
- head-to-tail cyclization: this method leverages the protein splicing capability of split inteins to achieve intracellular cyclization, a technique known as SICLOPPS (Split Intein Mediated Circular Ligation of Peptides and Proteins).
- in vitro cyclization: linear peptide libraries encoded by mRNA are translated in vitro and subsequently cyclized using chemical reagents. An example is represented by the RaPID technology.

### 4.1 SICLOPPS Technology

SICLOPPS, or split-intein circular ligation of peptides and proteins, is a method for synthesizing cyclic peptides within cellular environments. It offers a robust approach with high efficiency and purity. This technique can generate libraries containing up to 10^8^ cyclic peptides.^251^ It operates based on protein splicing, a natural process involving the removal of an internal protein segment, known as an intein, from a primary translation product. In SICLOPPS, split-intein domains, comprising separately expressed N-terminal (IN) and C-terminal (IC) segments of an intein, reassemble within cells to form an active intein (Scheme 10).

**Scheme 10:**
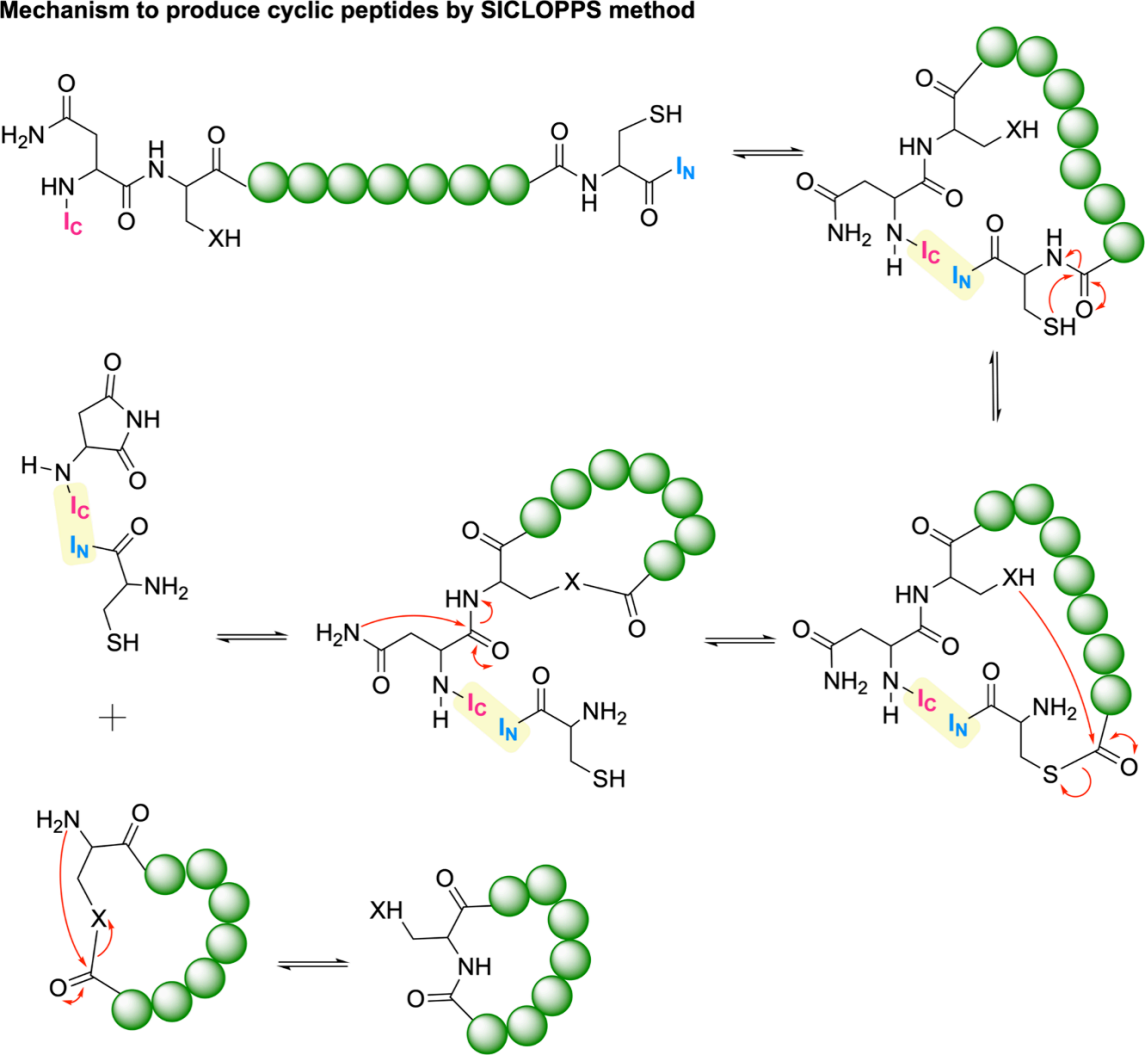
A Peptide Library Is Synthesized in Cells and Modified with Two Fragments of an Intein: One Attached to the N-Terminal Portion of the Peptide (IN) and the Other to the C-Terminal Portion (IC)^a^

a When the two fragments come into proximity, they reconstitute the complete intein. The activity of the intein triggers acyl substitution reactions, leading to the cyclization of the peptide library and the release of the intein. The first amino acid involved in the acyl substitution must be nucleophilic, such as cysteine or serine. If cysteine is present, a thioester intermediate forms, which subsequently reacts with the nucleophile at position 1 of the extein (X = O or S). This reaction creates a lariat structure that rearranges to yield a cyclic peptide.

The process initiates with the creation of a library of target peptides, also known as exteins, flanked by the C-terminal and N-terminal segments of a split intein (IC and IN, respectively), using conventional molecular biology methods.^252^ These fusion proteins undergo folding to activate the intein. To facilitate splicing, the initial amino acid of the target peptide must be a nucleophilic cysteine or serine. However, there are no further restrictions on the number or type of amino acids within the target peptide. This allows for the assembly of cyclic peptides of diverse sizes and sequences. In SICLOPPS, the peptide of interest is initially synthesized as a linear precursor with an N-terminal cysteine forming thioester. The thioester reacts with the nucleophile at position 1 of the extein (X = O or S), forming a lariat that rearranges to yield a cyclic peptide.

To create a plasmid library that encodes a diverse array of cyclic peptides, the extein sequence is modified using a degenerate oligonucleotide. The number of variable amino acid positions in the library is influenced by the transformation efficiency of the host organism, typically *E. coli*.^253^ The degenerate oligonucleotide encodes the variable segment as repeats of NNS or NNB, where N signifies any of the four DNA bases (A, C, G, or T), S represents either C or G, and B indicates C, G, or T. The NNS and NNB sequences cover 32 and 48 codons, respectively, including all 20 amino acids while excluding the UAA and UGA stop codons from the library. The design and synthesis of the degenerate oligonucleotide carefully control the number of randomized amino acids, as well as the inclusion of specific amino acids at designated positions, at the DNA level. Typically, 5 or 6 variable amino acids are introduced, ensuring that the total number of cyclic peptide library members (3.2 × 10^6^ and 6.4 × 10^7^, respectively) remains below the maximum number of *E. coli* transformants (typically 10^9^), which guarantees that each member of the library can be assessed. While it is possible to generate and screen larger cyclic peptide rings with more randomized amino acid positions, the size of such a library would still be limited by the transformation efficiency of the host organism. Historically, the trans-splicing split intein from DNA polymerase III (DnaE) derived from the cyanobacterium *Synechocystis sp*. (Ssp) PCC6803 has been utilized in the SICLOPPS approach. However, inteins from *Nostoc punctiforme* (Npu) have shown faster splicing rates and better tolerance to amino acid substitutions near the splice junctions compared to Ssp inteins. Despite these advantages, some variants from the Npu SICLOPPS library were found to be toxic to *E. coli*. To mitigate this issue, a SsrA degradation tag was integrated into the Npu SICLOPPS inteins, enabling the bacterial protease ClpXP to degrade the spliced inteins.

This high-throughput screening platform has been used to discover cancer treatments, particularly for identifying cyclic peptides that inhibit the HIF-1α/HIF-1β protein–protein interaction.^254^ HIF-1 is a heterodimeric transcription factor, and its role in angiogenesis, tumor growth, and metastasis is well established. In fact, the HIF-1α isoform is overexpressed in many cancers, and its activation, along with oncogene activation and loss of tumor suppressor function, is associated with HIF-1 activation. A HIF-1 bacterial reverse two-hybrid system (RTHS) was developed and used to screen a plasmid-encoded SICLOPPS library of 6-mer cyclic peptides to inhibit the dimerization of HIF-1. From a library of 3.2 million peptides, cyclo-CLLFVY was identified and proven to effectively inhibit the HIF-1α/HIF-1β protein–protein interaction both in vitro and in cells.

A more recent study combines SICLOPPS with next-generation sequencing (NGS) and biopanning to identify novel cyclic hexapeptides targeting tumors.^255^ The study presents a refined SICLOPPS screening method and workflow, incorporating pooled colony collection, NGS, and biopanning, which improves screening accuracy and reduces false positives. Among the peptides identified, cyclo-CLLFCL exhibited the highest activity both in vitro and in cellular assays.

Another study demonstrated how the identified cyclic peptide interferes with the Gag-TSG101 interaction, disrupting the complex and preventing HIV from budding out of the cell.^256^ The Gag-TSG101 interaction involves the binding of the HIV Gag protein to TSG101, a host cell protein. Because the peptide targets the host protein, it is less likely to be circumvented by viral mutations, in contrast to treatments that target viral functions directly.

### 4.2 RaPID Technology

While phage display peptide libraries offer extensive diversity, they may exhibit low affinity for the target and encounter issues related to the use of live cells and phages. mRNA-encoded libraries have emerged as a promising alternative to overcome these limitations.

mRNA display, a technique akin to phage display, is increasingly employed to discover new high-affinity peptide-based ligands for challenging therapeutic targets. It leverages synthetic oligonucleotides and cell-free transcription/translation to generate large, naïve libraries of mRNA-barcoded peptides, enabling rounds of selection to identify high-affinity binders to a protein target of interest. Initially developed in Nobel laureate Jack Szostak’s lab, significant innovations have since been made by Hiroaki Suga.^257,258^

The RaPID (Random nonstandard Peptide Integrated Discovery) platform, pioneered by Suga’s lab, represents a significant advancement in mRNA display technology. It introduces procedural improvements allowing for expedited selections within a week and incorporates unnatural amino acids (UAAs) using robust RNA aptamers called flexizymes. RaPID integrates mRNA display with a flexible in vitro translation (FIT) system, utilizing artificial flexible ribozymes to generate the desired aminoacyl-tRNA.^259^ This system allows for the incorporation of any amino acid, natural or synthetic, expanding the diversity of peptide sequences (Figure 13).^260^

**Figure 13:**
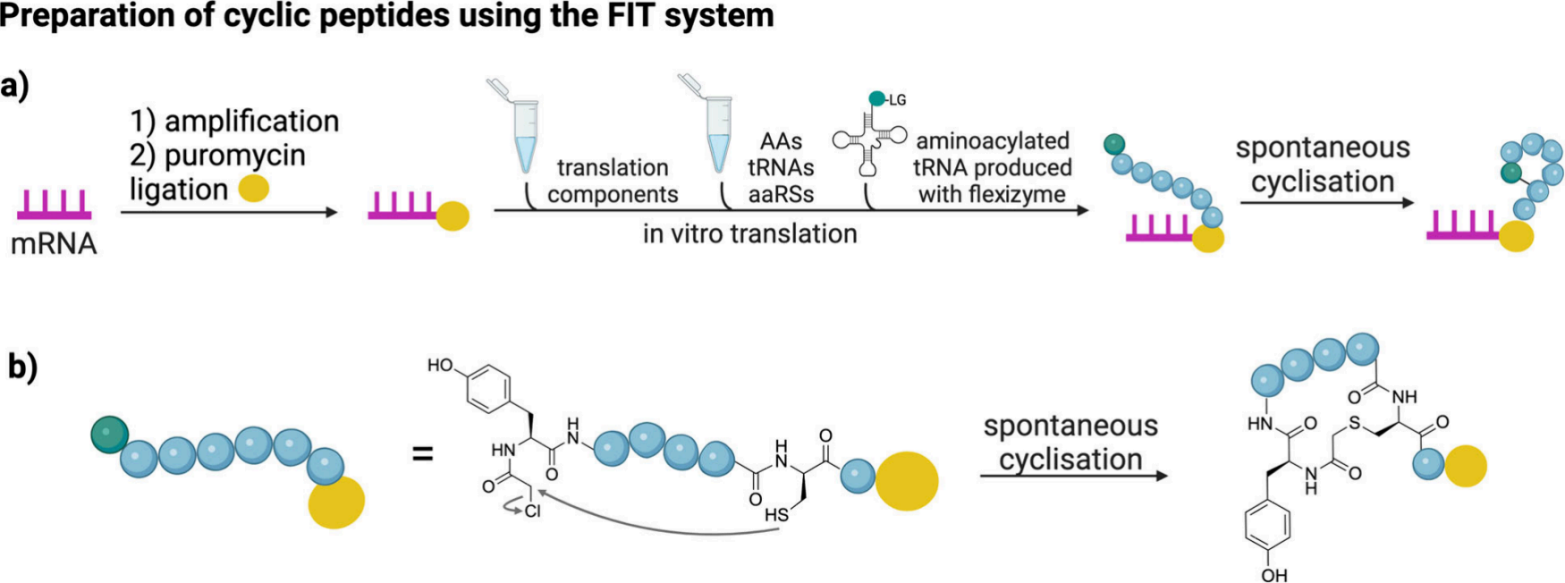
(a) RaPID leverages mRNA display technology and employs flexizymes to incorporate unnatural amino acids. This expands the diversity of peptides that cyclize spontaneously while still attached to their mRNA through puromycin (yellow sphere). (b) Cyclization occurs through the formation of a thioester, generated by a spontaneous reaction between N-(chloroacetyl)-Tyr (depicted as a green sphere) and a cysteine residue. Created in BioRender.

RaPID enables the construction and screening of extensive libraries of cyclic peptides, offering a technologically advanced approach compared to conventional methods.

In a standard RaPID experiment, mRNAs conjugated with puromycin are expressed using the FIT system, encoding N-chloroacetylated (ClAc) amino acids. Thioether macrocyclic peptides are generated by introducing unnatural N-(chloroacetyl)-d-Trp or N-(chloroacetyl)-Tyr into mRNA-encoded libraries, followed by spontaneous cyclization with Cys residues. The resulting products, cyclic peptides conjugated to puromycin, along with their respective mRNAs, are subjected to binding affinity assessment against target proteins using a systematic screening approach.^261,262^ Additionally, amino acid derivatives like 5-hydroxytryptamine and benzylamine are synthetically assembled into linear peptidic sequences, then cyclized using photogenic oxidative coupling to yield fluorescent cyclic peptides.^263^

The RaPID system enabled the identification of thioether-macrocyclic peptides with high affinity for the target protein. However, peptides produced via this system are unprotected, which imposes constraints on macrocyclization that must be chemo- and regioselective and occur under mild, aqueous conditions. As a result, traditional macrolactonisation methods commonly used in solid-phase peptide synthesis cannot be easily applied during ribosomal peptide synthesis. To overcome this limitation, the Suga group developed an innovative approach for generating macrolactones directly within the context of ribosomal peptide synthesis.^264^ This method involves incorporating the SPCG motif into the peptide sequence. During the standard RaPID process, cysteine forms a self-acylating macrocycle, followed by serine forming an *O*-acyl isopeptide through an intramolecular *S*-to-*O* acyl transfer. This post-translational modification occurs spontaneously, producing cyclic depsipeptides in a one-pot reaction with variable sizes, ranging from 7 to 17 residues. The study found that proline and glycine play a role in facilitating the correct arrangement of residues for the acyl transfer. However, the most critical factor is the positioning of serine and cysteine, as the SXCX motif is essential for the transfer process. For instance, the CPSG motif was observed to be less efficient in facilitating this reaction.

RaPID has transformed mRNA display into a powerful tool for identifying potent peptide inhibitors, leading to the establishment of successful companies like PeptiDream. While integrating unnatural amino acids into biological libraries presents challenges, advancements in genetic technologies have facilitated the engineering of mRNAs and tRNAs. In recent years, several biological cyclic peptide libraries incorporating unnatural amino acids have been reported.^265–267^ The thioether linkage, utilized in the RaPID system, holds significance in the development of cyclic mimetics containing Cys.

### 4.3 Chemo-Enzymatic Synthesis

Methods such as RaPID and other recombinant and enzymatic approaches are considered green technologies because they do not require the use of harmful solvents or reagents, making them safer for both the environment and human health. However, in pharmaceutical companies, peptides are still synthesized using chemical methods, often requiring a significant excess of protected amino acid monomers, costly activation agents, harsh reagents, and large amounts of organic solvents. This approach, especially at scale, generates considerable waste.^268,269^ While efforts have been made to develop greener synthetic processes, challenges in achieving sustainable production and efficient purification persist.^270^ The production of large peptides and proteins typically involves the synthesis of smaller peptide fragments, which are then coupled. In the pharmaceutical sector, the use of protected peptide fragments for coupling is a common strategy for therapeutic peptide production while condensation of unprotected fragments has proven less efficient and practical.^270,271^

Recent successes in the development and market approval of long peptides (>30 residues) containing unnatural amino acids underscore the utility of chemical approaches, particularly hybrid processes, for minimizing impurities, streamlining purification, and meeting stringent regulatory standards. For example, the hybrid synthesis of Tirzepatide integrates solid-phase peptide synthesis (SPPS) and liquid-phase peptide synthesis (LPPS), facilitating impurity control and purification.^272^ Chemo-enzymatic peptide synthesis (CEPS) presents an alternative, leveraging enzymes for fragment condensation, as demonstrated by ligases.^273,274^ CEPS, a promising green alternative, employs water-based conditions for fragment coupling instead of organic solvents such as those used in LPPS.^275^ Nonetheless, SPPS remains the primary method for fragment synthesis, often utilizing dimethylformamide as a solvent.

Biocatalysis has had a transformative impact on the synthesis of small molecules (e.g., sitagliptin), but its application to medium-sized molecules like peptides and oligonucleotides has been comparatively limited.^276^ Enzymes such as sortases, butelases, trypsiligases, and engineered variants of subtilisins like omniligases, subtiligases, and peptiligases have been employed for peptide fragment ligation, with significantly advancing CEPS.^277–279^ These enzymes facilitate the production of linear and cyclic peptides, protein conjugates, and therapeutic peptides.^280–282^ Notably, omniligase-1, a broad-specificity ligase engineered from subtilisin BPN’, was successfully used to synthesize exenatide, and peptiligase has been applied for gram-scale quantities of therapeutic peptides such as thymosin-α1, exenatide, and the kalata B1 variant T20K.^283^ These results highlight the potential of CEPS for adoption in sustainable, large-scale manufacturing of therapeutic peptides.

Typically, CEPS employs Cam esters as acyl donors and catalyzes the condensation of C-terminal peptide esters with N-terminal peptide fragments in aqueous conditions. This method minimizes hydrolysis by favoring the condensation reaction kinetically. Furthermore, the engineering of optimized enzymes significantly reduces the formation of side products, such as those arising from ester hydrolysis or unintended coupling with other N-terminal amines present in the reaction mixture.^284^

Pawlas et al. employed the CEPS method to synthesize exenatide, specifically by preparing the fragments H-1–21-O-Cam-L-NH_2_ and H-22–39-NH_2_ through solid-phase peptide synthesis and ligating them using omniligase-1.^276^ Their study demonstrated that enzymatic ligation proceeds efficiently under physiological pH and in the presence of 10% acetonitrile as a cosolvent, working effectively with both crude and purified fragments. However, the carboxamidomethyl (O-Cam) linker exhibited limited stability at high temperatures. To address this, the aromatic 4-hydroxymethylbenzoic acid (HMBA) linker was evaluated as a more robust alternative. Using this approach, the H-1–21-HMBA-K fragment was synthesized with high yield and purity and subsequently coupled to the H-22–39-NH_2_ fragment on a large scale via omniligase-1 catalysis, yielding 53 g of crude exenatide. The process was further assessed in terms of manufacturing cost, complete E factor (cEF), and carbon intensity (CI), comparing it to both conventional and lab-scale CEPS benchmark processes. The results revealed that the CEPS process employing the H-1–21-HMBA-K fragment was not only successfully scaled up but also demonstrated significant improvements in economic efficiency and environmental sustainability compared to both benchmark methods.

The Cabri group reported another hybrid system based on green solid-phase peptide synthesis (GSPPS) for the preparation of peptide fragments, combined with omniligase-1 for fragment coupling.^285^ This approach was tested for the synthesis of liraglutide. Initially, two liraglutide fragments, H-(1–11)-CamFK-NH_2_ and H-(12–31)–OH, were synthesized (Figure 14).

**Figure 14:**
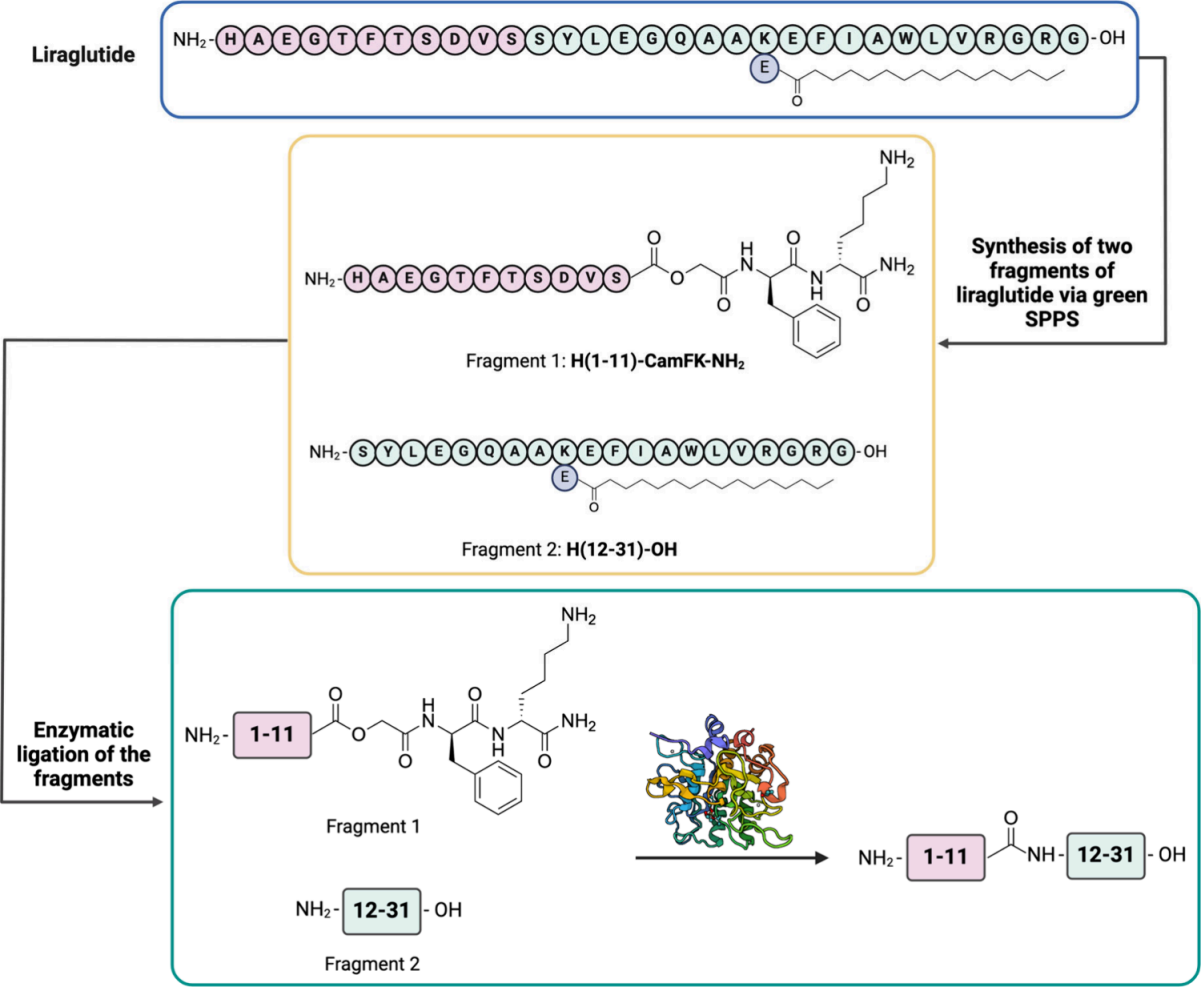
CEPS (Chemo-Enzymatic Peptide Synthesis) is an approach that combines organic chemistry strategies with enzymatic catalysis to form peptide bonds. Specifically, the peptide of interest (e.g., liraglutide, shown in the figure) is synthesized in fragments using solid-phase synthesis and green solvents. The crude fragments are then condensed enzymatically in an aqueous solution to produce the complete peptide. This method offers a more sustainable alternative to conventional SPPS in DMF and results in peptides with fewer impurities, thanks to the fragment condensation process. Created in BioRender.

The first fragment featured a C-terminal activated with the carboxamidomethyl ester (OCam) and was extended by two amino acids, phenylalanine and lysine. The addition of the two amino acids following the Cam ester moiety improved both the solubility and substrate interaction with the enzyme, further enhancing coupling efficiency. However, the use of the OCam ester significantly reduced atom economy as the -OCam-FK-NH_2_ fragment is not present in the final product. Additionally, introducing -OCam-FK requires more solvent and reagent, negatively impacting the overall greenness of the process. This limitation further emphasized the need to replace DMF with greener alternatives. The group evaluated various green solvents and their combinations, identifying N-butylpyrrolidone/dimethyl carbonate (8:2) as the most effective mixture. This combination provided good results compared to standard conditions with DMF, yielding higher purity and greener process metrics. To further enhance process efficiency, fragment ligation was performed on crude peptides. This strategy, inspired by previous work on CEPS, demonstrated significantly lower process mass intensity (PMI) metrics compared to approaches using purified fragments. The ligation reaction was monitored by HPLC, and after 24 h, it resulted in an 81% yield in solution.

Biocatalysis holds significant potential for peptide cyclization. However, commercially available cyclases are limited in their ability to cyclize peptides smaller than 10 amino acids. This limitation underscores the need to investigate alternative nonribosomal cyclases, particularly those capable of cyclizing natural peptides with scaffolds ranging from 4 to 15 residues. Among these, the SurE cyclase, a key enzyme in the surugamide biosynthetic pathway from various Streptomyces species, is of particular interest due to its remarkable substrate tolerance, making it a promising candidate for biocatalytic applications.^286^

The SurE enzyme can be employed in combination with the CuAAC reaction to produce bicyclic peptides. After the enzyme catalyzes the head-to-tail cyclization, the subsequent click reaction between the azide and alkyne, introduced immediately after the enzymatic step, can occur. It was sufficient to add a copper-based catalyst and ascorbic acid directly into the enzymatic reaction mixture. This two-step cyclization proceeds in a one-pot reaction without the need to purify the monocyclic intermediate. This chemoenzymatic strategy facilitated the efficient synthesis of bicyclic peptides containing hexa-, octa-, and undecapeptidyl head-to-tail cyclic scaffolds.^287^

## 5 Strategies without a Backbone in Peptidomimetics

A category of peptidomimetics comprises molecules devoid of a peptide main chain, featuring only side chains. These structures are commonly employed to emulate secondary structures. However, their lack of a peptide backbone renders them highly flexible. As a result, there is no distinct global minimum energy state corresponding to a specific secondary structure. Instead, they resemble various secondary conformations concurrently and can adapt to diverse binding scenarios. For instance, they may occupy compact enzyme cavities typically inaccessible to other peptides or peptidomimetics with a backbone, especially when the precise binding conformation is unknown.^288^ The key consideration in designing such molecules is to avoid high thermodynamic costs and insurmountable kinetic barriers, ensuring easy obtainment of the desired structures. Therefore, their backbone designs must incorporate moieties restricting degrees of freedom.^289^

These peptidomimetics are alternatively termed minimalistic or universal mimics, reflecting the absence of a backbone or the capability to adopt any secondary structure. Pioneering examples were introduced by Hirschmann and Smith, who designed β-turn analogues. Their approach involved incorporating additional molecules such as sugars, catechols, and steroids to position significant side chains at appropriate distances, elucidating their activity.^290,291^ The Hamilton group proposed minimalist helical mimics, utilizing terphenyl scaffolds to present side chains in optimal orientations. In contrast to the structures suggested by Hirschmann and Smith, these helical mimetics exhibit sufficient rigidity.^292^

Additional examples of minimalist peptidomimetics include pyrrolinone-pyrrolidine oligomers derived from tetramic acids as critical starting materials. These mimetics, featuring two noncontiguous side chains, can adopt the conformation of three different helix types and both parallel and antiparallel β-sheets. The mimetic with three noncontiguous side chains predominantly assumes antiparallel β-sheet conformations.^293^

When paired with Val, cyclophanes serve as minimalist cyclic peptidomimetics. Val-cyclophanes exhibit self-assembly into an organized architecture based on a fibrillar network. The resulting supramolecular network is assembled through physical interactions and entraps a diverse range and substantial amounts of solvents, forming robust gels.^294^

## 6 Applications of Peptidometics

### 6.1 Mimetics in Diabetes and Obesity Studies

Obesity and overweight pose a significant global health concern, affecting millions of people, including both adults and children. Obesity is strongly linked with dyslipidemia, characterized by elevated blood levels of low-density lipoprotein (LDL) and cholesterol, and is also a major risk factor for type 2 diabetes mellitus (T2DM).^295^ Historically, type 2 diabetes has been treated with metformin, which remains a first-line therapy alongside sulfonylureas, thiazolidinediones, and insulin.^296–298^ Insulin is also a medicine commonly used to control glucose levels. Insulin is a peptide hormone initially extracted from animal sources, was later produced through recombinant DNA technology, yielding a safer product that minimized patient allergic reactions. Advances in genetic engineering enabled specific amino acid modifications to enhance the ADMET properties of insulin.^299^ Notably, researchers have engineered insulins that avoid hexamer formation—an inactive storage form in the body—and developed both fast-acting (lispro, aspart, glusine) and slow-release (glargine, detemir, deglutec) insulin analogues, offering various therapeutic options for patients. Insulin is also effective in managing type 1 diabetes (Figure 15).^300^

**Figure 15:**
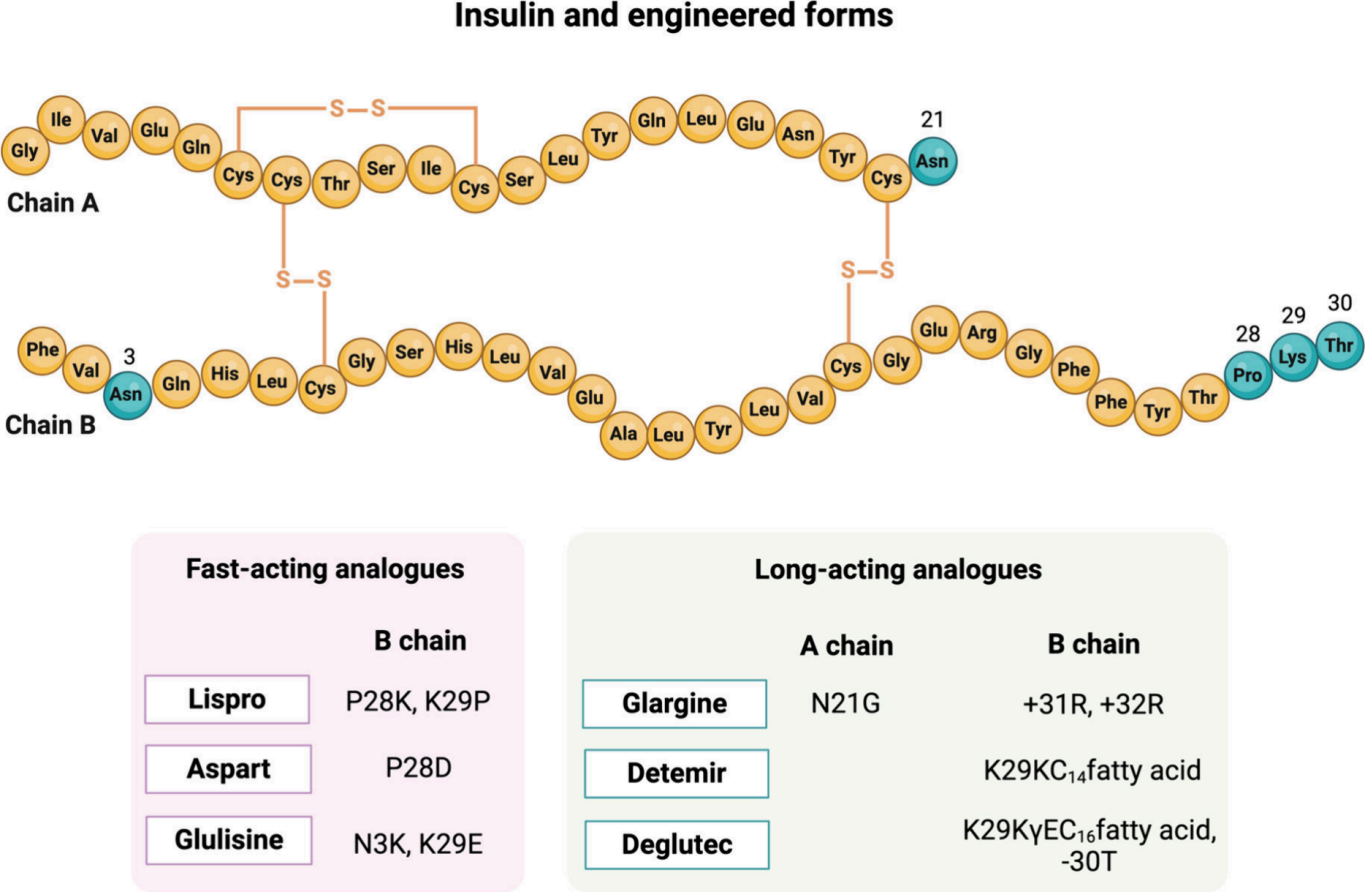
Structure of insulin, consisting of two chains, is shown at the top. Several engineered insulin analogues have been developed through amino acid modifications or additions. In the figure, green spheres indicate residues in the original insulin sequence that have been modified. Most engineered insulins involve modifications to residues on the B-chain, particularly at positions 3, 28, 29, and 30. For example, position 29 has been modified with the addition of a lipid chain, as in detemir and degludec, which exhibit long-lasting action. Degludec also lacks threonine at position 30. Long-lasting action can also be achieved by adding arginine residues to the B-chain, extending it to 32 residues, as in glargine. By modifying the charges at positions 3, 28, and 29 of the B-chain, fast-acting analogs such as lispro, aspart, and glulisine can be developed. Created in BioRender.

Amlyn, a peptide hormone cosecreted with insulin by pancreatic β-cells in response to meals, is present at low levels in type 1 diabetes patients but elevated in those with type 2 diabetes. An amylin analogue, pramlintide, was approved in 2005;^301,302^ it is coadministered with insulin at mealtime, often in combination with metformin and/or sulfonylureas. Human amylin is highly amyloidogenic, but studies showed that rat amylin, which includes proline residues, does not readily form amyloid aggregates. As a result, prolines were substituted for Ala25, Ser28, and Ser29 in human amylin to develop the pramlintide analog.^303^

However, maintaining glucose homeostasis can be challenging for certain patients, necessitating new therapeutic targets and more effective treatments. A major advancement in diabetes treatment was achieved with the development of glucagon-like peptide-1 receptor agonists (GLP-1RAs). GLP-1RAs mimic the action of the natural peptide GLP-1 by binding to the GLP-1 receptor (GLP-1R), thus producing effects similar to those of the endogenous peptide. GLP-1 is a peptide hormone belonging to the incretin family, released in two phases: an initial phase approximately 10–15 min postmeal, followed by a secondary release 30–60 min later from intestinal L-cells. Its active forms include GLP-1(7–37) and amidated GLP-1(7–36).^304^ GLP-1 receptors are present on the membranes of various cell types, enabling GLP-1 and its agonists to exert multiple effects throughout the body, impacting not only the digestive system but also the brain, heart, kidneys, and muscles (Figure 16).^305^

**Figure 16:**
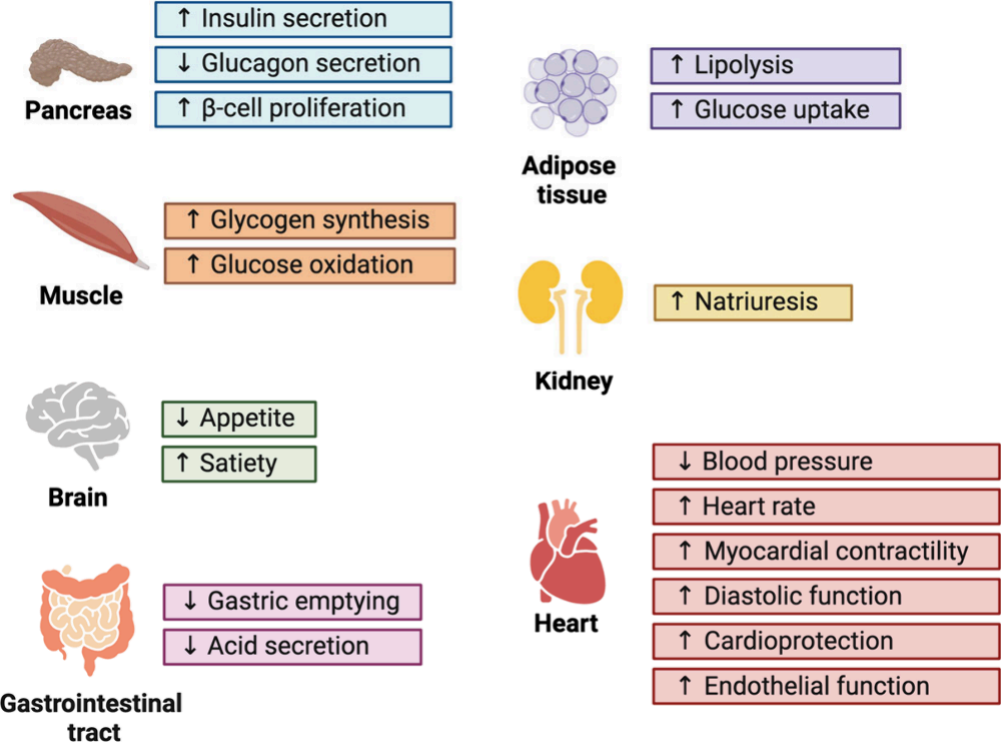
GLP-1 exerts multiple effects by binding to its receptor, which is expressed on the membranes of various cell types. GLP-1 receptors are found in the pancreas, muscle tissue, gastrointestinal tract, brain, heart, kidneys, and adipose tissue. GLP-1 helps regulate blood glucose levels by stimulating insulin release, promoting glucose storage as glycogen, and increasing satiety. Additionally, it has cardiovascular benefits, such as lowering blood pressure, and plays a role in combating obesity. Created in BioRender.

In the gastrointestinal tract, GLP-1 enhances insulin secretion from pancreatic β-cells, triggers somatostatin release from δ-cells, inhibits glucagon release from α-cells, and slows gastric emptying. This leads to improved blood glucose regulation and promotes satiety, which assists in weight loss (Figure 17).

**Figure 17:**
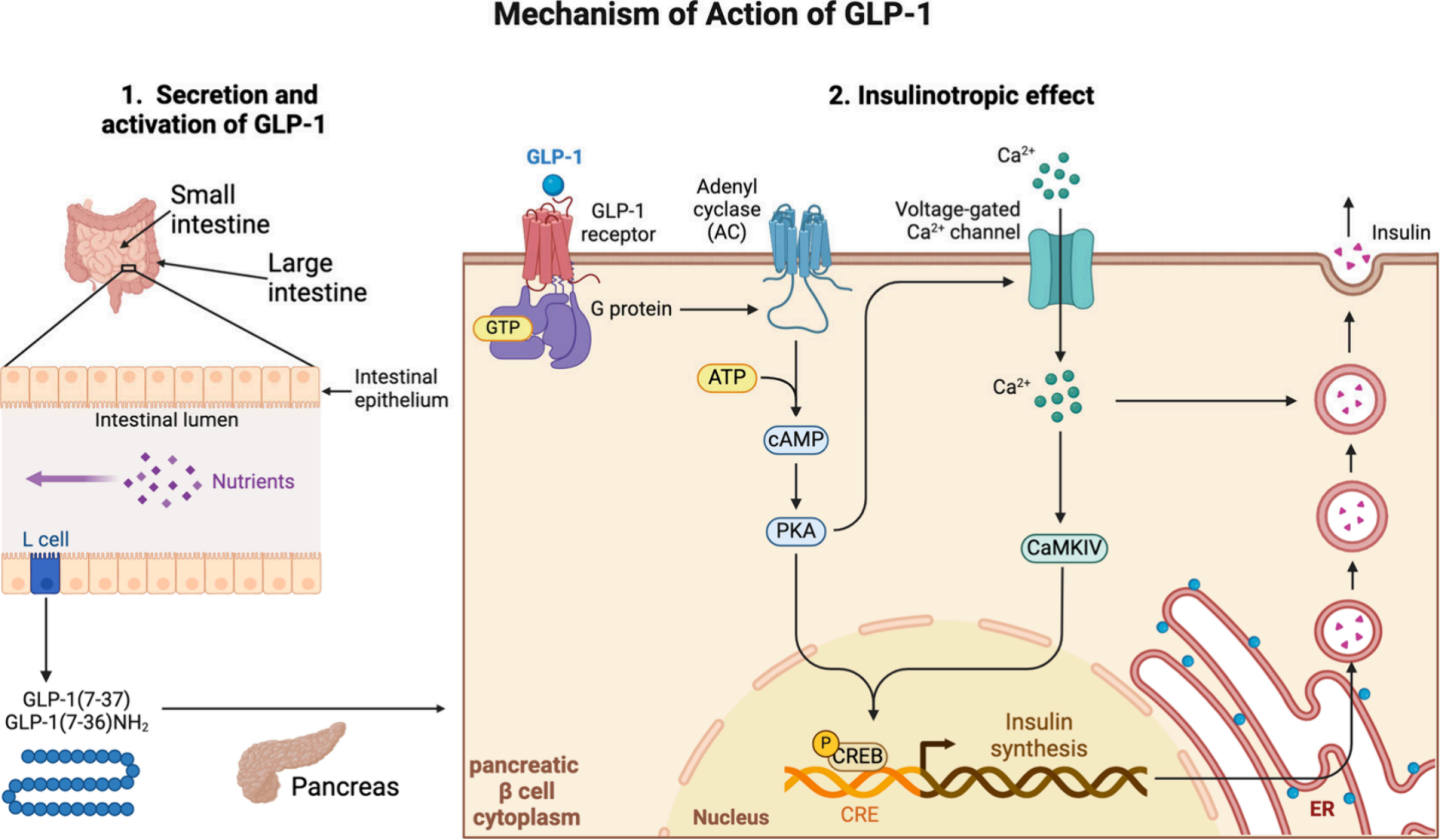
When nutrients enter the small intestine, L-cells synthesize and secrete GLP-1, which is proteolytically processed to yield the active forms GLP-1(7–37) and GLP-1(7–36)­NH_2_. Active GLP-1 binds to its receptor on the cell membranes of pancreatic β-cells. This receptor is a G protein-coupled receptor, and its interaction with GLP-1 triggers an intracellular signaling cascade that leads to the synthesis of insulin. Insulin is then secreted via vesicles that fuse with the plasma membrane. Created in BioRender.

This glucose-dependent insulinotropic effect means that GLP-1 actions are triggered only when blood glucose levels are elevated above normal fasting plasma levels. This is particularly beneficial for diabetes management as it reduces the risk of hypoglycemia—a common side effect of several antidiabetes drugs, including insulin.^306^ Additionally, GLP-1 demonstrates positive effects on multiple tissues and organs, with the widespread presence of GLP-1R suggesting that GLP-1 plays broader roles beyond glucose metabolism. Significant efforts were made to develop GLP-1 as a therapeutic drug after researchers observed that intravenous injections of GLP-1 had beneficial effects on insulin secretion and blood glucose control in patients with type 2 diabetes.^307^

Structure–activity studies using alanine-scanning have revealed that residues His7, Gly10, Phe12, Thr13, Asp15, Phe28, and Ile29 are critical for GLP-1 receptor interaction, with the active form identified as GLP-1(7–37).^308^ The natural active GLP-1(7–37), hereafter referred to simply as GLP-1 in this article, is rapidly degraded by dipeptidyl peptidase 4 (DPP-4), which cleaves between residues Ala8 and Glu9, and is swiftly cleared renally within 1–2 min.^304^ Substituting the position-8 residue improves DPP-4 resistance, though the peptide remains susceptible to rapid renal clearance. DPP-4 is the primary enzyme responsible for GLP-1 inactivation. Replacing Ala8 with the α,α-dimethyl amino acid Aib (α-aminoisobutyric acid) or Gly has effectively prevented unwanted proteolytic cleavage.

In the early 1990s, a GLP-1 analogue was discovered in the venom of the Gila monster. This peptide, exendin-4, exhibited 53% sequence homology with human GLP-1 and proved highly stable against DPP-4 degradation and resistant to renal clearance in humans.^309^ Exendin-4 contains glycine at position 8, replacing alanine and avoiding DPP-4 cleavage. It also has a tail of proline, alanine and serine residues (PASylation; Section 2.8) followed by three proline residues that form a steric shield around the peptide, reducing protease accessibility. Specifically, this tail forms a “Trp cage” at the C-terminus, which protects the peptide from degradation by another protease, neutral endopeptidase (NEP).^310,311^ The C-terminal tail also increases the peptide’s molecular size and hydrodynamic radius, reducing renal filtration and extending its half-life to approximately 2.4 h.

Exendin-4 acts as a GLP-1 receptor agonist and served as the foundation for the pharmaceutical formulation marketed as exenatide, sold under the brand name Byetta by AstraZeneca in 2005 and later by Bristol-Myers Squibb. The sequence of exenatide is identical to that of exendin-4. The drug is administered to adults twice daily via subcutaneous injection before main meals.^312^ In 2017, a long-acting formulation, Bydureon BCise, was approved, enabling once-weekly administration.^313^ Further advancements have been made in developing GLP-1RAs. Currently, the FDA has approved seven different GLP-1RAs for the treatment of type 2 diabetes and obesity (Figure 18, Table 1).^314^

**Figure 18:**
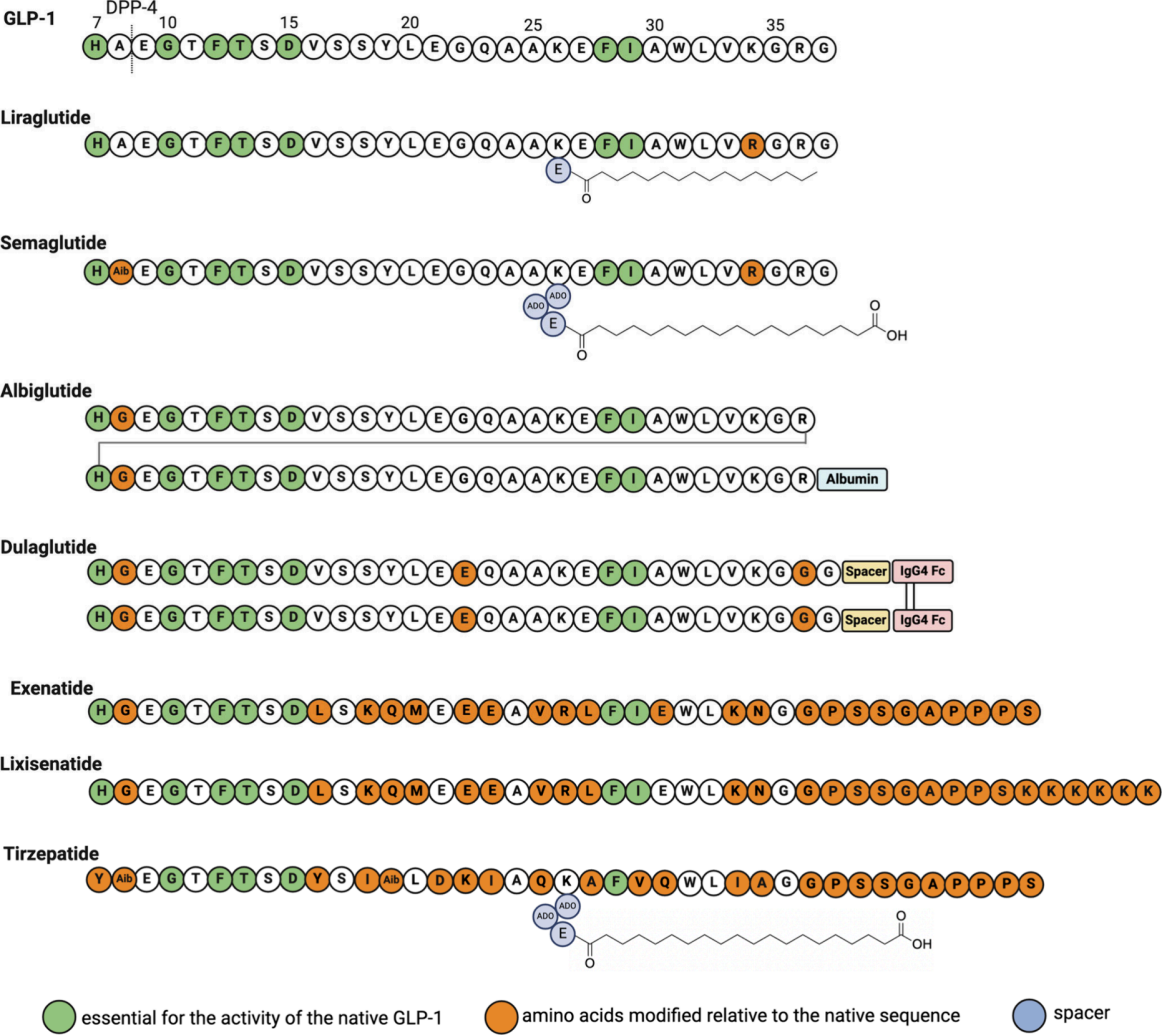
Native GLP-1 sequence contains essential amino acids (green) that are critical for its interaction with the receptor. In designing GLP-1 analogues, efforts have been made to preserve these essential residues while modifying less critical ones. Modifications relative to the native sequence are shown in orange. A key modification common to all analogues, except liraglutide, is at position 8, where alanine—a target for proteolytic cleavage—has been replaced with Aib or Gly to enhance stability. Additional changes have been made to internal residues or at the termini, such as in exenatide and lixisenatide, which feature added residues. Residues left unchanged are shown in white, while those in blue represent spacers attached to the side chain of lysine 26 to link lipid moieties, as seen in liraglutide and semaglutide. In contrast, albiglutide and dulaglutide incorporate proteins at the C-terminus: albumin in the case of albiglutide and an antibody for dulaglutide. Both albiglutide and dulaglutide feature two copies of the GLP-1 sequence. Tirzepatide is unique, as it combines key sequences from GLP-1 and GIP, enabling the analogue to bind to both receptors and provide more effective treatment for diabetes and obesity. Tirzepatide also includes a lipid tail to increase the circulation time of the drug. Created in BioRender.

One of these GLP-1RAs is liraglutide, approved in 2010 and marketed by Novo Nordisk in two formulations: Victoza for diabetes treatment and Saxenda for weight loss.^315^ In 2014, dulaglutide, marketed by Lilly as Trulicity, received FDA approval.^316^ Also approved in 2014, albiglutide was developed by GSK and marketed as Tanzeum; however, it was later withdrawn from the market for commercial reasons.^316^ Lixisenatide, produced by Sanofi, was approved in 2013 and sold as Lyxumia in Europe.^317^ In 2016, it was also approved under the brand name Adlyxin in the United States, though it was later discontinued for business reasons.^318^ In 2017, semaglutide received FDA approval and was marketed by Novo Nordisk as Ozempic, with an additional oral formulation approved in 2019 as Rybelsus and a version for weight management called Wegovy approved in 2021.^319–321^ Semaglutide has shown superior efficacy compared to liraglutide. In 2022, Lilly received FDA approval for tirzepatide, marketed as Mounjaro.^322^

It is established that increasing the molecular weight of peptides is crucial in drug development, as steric hindrance enhances stability against degradation. Additionally, the larger size reduces renal clearance, thereby prolonging plasma circulation time. In the development of new GLP-1 receptor agonists, researchers have recognized that increasing the molecular weight of peptides can be achieved through various strategies and molecules. Lixisenatide is a modified form of exenatide, featuring a longer peptide chain composed of 44 amino acids, with the C-terminal proline residues replaced by six lysines. This modification has demonstrated an increased affinity for the GLP-1 receptor compared to both exenatide and native GLP-1, and lixisenatide can be administered once daily.^323^ The lysine tail enhances receptor binding affinity; however, it does not significantly extend the half-life of lixisenatide, which is approximately 3 h, comparable to exenatide’s half-life of 2.4 h. Although amino acid addition and substitution effectively reduce proteolytic degradation, modified GLP-1 analogues continue to face rapid renal clearance, which limits efforts to extend peptide circulation time. Consequently, strategies beyond adding amino acids, such as those employed in exenatide, have been explored. These include lipidation and conjugation to larger proteins, which may enhance stability and prolong the circulation time of the peptides in the bloodstream (Section 2.8).^324^ Lipid conjugation has been employed in the development of long-acting analogues such as insulin detemir, insulin degludec, liraglutide, semaglutide, and tirzepatide, allowing for administration either once daily (as seen with detemir, degludec, and liraglutide) or once weekly (as with semaglutide and tirzepatide). The incorporation of lipids not only increases the peptide’s size but also enhances its binding to albumin, significantly improving plasma circulation compared to the addition of the proline, serine, and alanine tail in exenatide. Albumin acts as a protective shield for the peptide, preventing protease degradation. Furthermore, lipid conjugation provides additional benefits, including delayed release from the injection site and reduced immunogenic response.^325^ In the case of liraglutide, it has been observed that the lipid tail promotes the aggregation of the peptide into hexa-, hepta-, or octamers, remaining in this oligomeric form under specific pH or ionic strength conditions.^326^ When these conditions change, the oligomers dissociate into monomers, which enter circulation and bind to albumin noncovalently. This aggregation mechanism is responsible for the slow release of the peptide at the injection site. Overall, incorporating the lipid significantly enhances the pharmacokinetics of the peptide.

Like all peptides, GLP-1 analogues have a preferred orientation for binding to their receptor. The N-terminus contains critical residues necessary for receptor activation, and attaching the lipid tail to this end significantly reduces activity.^327^ Consequently, hydrophobic components are typically conjugated to the C-terminus, with a spacer used between the lipid and the peptide to enhance flexibility. For liraglutide, the spacer is γ-glutamic acid at position Lys26, while the lipid component is palmitic acid. In semaglutide, the lipid tail is longer, consisting of 18 carbon atoms and featuring two carboxylic acid groups. The spacer in semaglutide includes one γ-glutamic acid and two 8-amino-3,6-dioxaoctanoic acid (ADO) units.^328^ Both liraglutide and semaglutide maintain the same sequence as natural GLP-1. Their distinguishing characteristics compared to the native peptide include modifications at positions 26 and 34, where lysine is conjugated to a lipid chain or substituted by arginine, and at position 8 in semaglutide, where an Aib is incorporated to enhance stability against DPP-4 degradation.^329^

Another successful strategy involves the conjugation of peptides to proteins, as exemplified by albiglutide and dulaglutide. Albiglutide is linked to human albumin, while dulaglutide is covalently attached to a fragment of human IgG4.^139,330^ These analogues consist of two peptide chains with sequences similar to natural GLP-1, incorporating a few mutations to enhance stability, particularly at position 8, where glycine replaces alanine.

In dulaglutide, the two peptide chains are identical and truncated, linked covalently to the antibody fragment via a flexible linker. Their stability is ensured by a disulfide bridge formed between the two IgG4 Fc regions. In contrast, albiglutide also contains two peptide copies; however, they are arranged sequentially, with conjugation to human albumin occurring solely at the C-terminus.^331^

Another approach to improve circulation half-life of GLP-1 agonists involves the use of carriers such as polymeric hydrogels, nanoparticles, or microparticles. However, when it comes to peptides, only poly­(lactic-*co*-glycolic acid) (PLGA) has been approved by the FDA, which considers it a safe and effective carrier for therapeutic peptides. PLGA is biocompatible and is gradually degraded by the body, allowing for prolonged drug release.^332^ PLGA has also been employed in formulations for antidiabetic peptides. For instance, in a collaboration between Lilly, Amylin, and Alkermes, PLGA microspheres were developed to deliver exenatide. Although a PLGA formulation capable of releasing the drug over several months is theoretically possible, the exenatide formulation, marketed as Exenatide QW (Bydureon) by AstraZeneca, was limited to a once-weekly injection.^333^ Upon subcutaneous administration, exenatide is initially released from the surface and surface pores of the microspheres during the first 48 h. The product was designed for a slow initial release phase to minimize adverse effects such as nausea and vomiting.^334^ The second phase involves the gradual diffusion of the drug from the polymer matrix, with peak plasma concentrations observed after approximately 2 weeks.^334^ In the third phase, degradation of the microparticles occurs. Overall, drug release with this technology lasts for about 11 weeks. Before administration, PLGA microparticles must be suspended in a phosphate buffer. Subsequently, the Bydureon Bcise device was developed, in which PLGA particles are suspended in triglycerides and delivered via a single-dose autoinjector.^334^

As noted, one limitation of PLGA microparticles in the Bydureon formulation is the inability to achieve a consistent release rate of GLP-1RAs. To address this issue and provide steady, continuous delivery of GLP-1RAs, other controlled-release devices such as osmotic pumps have been explored. A representative product is ITCA 650, developed by Intarcia Therapeutics.^335^ ITCA 650 is an implantable subdermal osmotic titanium mini-pump designed for the continuous release of exenatide over a period of up to six months. In 2024, the FDA rejected the New Drug Application (NDA) for ITCA 650 following a unanimous vote by an FDA advisory committee, which raised significant safety concerns. These concerns included potential risks of acute kidney injury and cardiovascular side effects. Additionally, the delivery system, which operates on a continuous release mechanism, was criticized for its inconsistent drug release, further jeopardizing patient safety. This rejection marks the third setback Intarcia company has encountered regarding ITCA 650.^336^

The discussion surrounding tirzepatide is distinct, as it functions as a dual agonist by combining the actions of two incretins: gastric inhibitory polypeptide (GIP) and GLP-1 (Figure 19).

**Figure 19:**
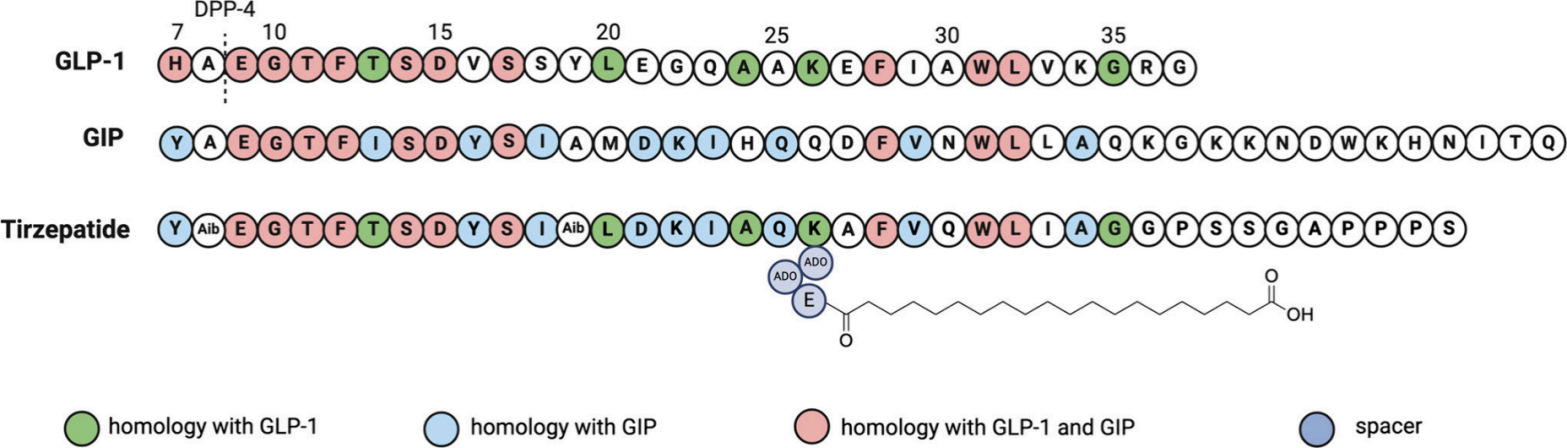
Tirzepatide features a sequence homologous to both GIP and GLP-1, giving the peptide dual action by targeting two receptors. The amino acids at the C-terminus enhance stability, while the lipid tail prolongs circulation time, improving its pharmacokinetic profile. Created in BioRender.

GIP, like GLP-1, stimulates insulin secretion. The dual agonist effect of tirzepatide appears to confer a superiority over semaglutide, evidenced by a greater reduction of more than 2% in glycated hemoglobin (HbA1c), a marker of chronic hyperglycemia.^337^ Tirzepatide comprises 39 amino acids and is based on the GIP sequence with several modifications. Similar to semaglutide and liraglutide, it contains a lipid tail, specifically a C-20 fatty acid (1,20-eicosanedioic acid) linked to lysine at position 26 via a spacer moiety. Additionally, it incorporates two Aib residues at positions 8 and 19, along with amidation at the C-terminus.

Various synthetic strategies for the production of GLP-1 have been reported. These approaches can be broadly classified into two main categories: recombinant techniques and fully synthetic strategies.

A recombinant strategy employed by Novo Nordisk involves the production of liraglutide through recombinant DNA techniques, followed by the in vitro attachment of a γ-(Pal-Glu-O*t*Bu) moiety to Lys26. In this approach, the peptide is initially synthesized as a precursor with an N-terminal extension, which serves multiple functions: it protects the precursor molecule from proteolytic degradation within the host cell or culture medium, facilitates purification, and minimizes fibril formation. Following expression and purification, the N-terminal extension is removed, exposing Lys26. Under controlled in vitro conditions, a lipid chain, specifically a palmitic acid moiety, is subsequently conjugated to Lys26 via a glutamic acid linker.^338^ To ensure that lipid chain attachment occurs exclusively at Lys26 and does not affect Lys34, the latter was replaced with arginine. This substitution prevents nonselective lipidation, ensuring site-specific modification of the peptide. Another approach to obtain liraglutide involves the use of fusion peptides. A fusion peptide consists of three components: the target peptide, an affinity tag, and a cleavable tag. Following recombinant production, the peptide is purified using affinity chromatography. In the final step, the cleavable tag is removed, yielding the purified peptide.^339^

Recombinant techniques have also been employed for the production of albiglutide and dulaglutide. These two GLP-1 analogs are large molecules composed exclusively of naturally occurring amino acids, making in vivo expression the most suitable approach for their production.^139,340^ For the synthesis of the dulaglutide dimer, the Fc portion of IgG4 was modified to introduce serine residues. Following protein synthesis, the two Fc chains spontaneously dimerize through the formation of disulfide bonds, resulting in the final dimeric structure.

In chemical synthesis, challenges such as peptide aggregation and the presence of numerous deletion peptides, which often coelute with the target peptide, are commonly encountered.^270^ The branched structure, combined with fatty acid modifications and a distinct amino acid sequence, promotes peptide folding and aggregation, making the chemical synthesis of high-purity liraglutide and semaglutide particularly challenging. This complexity was one of the key reasons why recombinant approaches were initially favored for industrial production.

In general, research on chemical synthesis has focused on optimizing reaction conditions through the use of efficient and selective coupling reagents, orthogonal protecting groups, specialized resin linkers, and the incorporation of pseudoprolines and depsipeptide intermediates into the peptide sequence.

Some orthogonal protecting groups employed in the total chemical synthesis of liraglutide and semaglutide are Alloc, Mtt, or ivDe on the Lys residue to enable selective modification.^341–345^ With this approach, at the end of the SPPS process, the protecting group on the lysine residue is removed, followed by the coupling of N^α^-protected Glu-O*t*Bu. Subsequently, N^α^-deprotection is performed, allowing for the final conjugation of Pal–OH to complete the synthesis (Scheme 11).

**Scheme 11:**
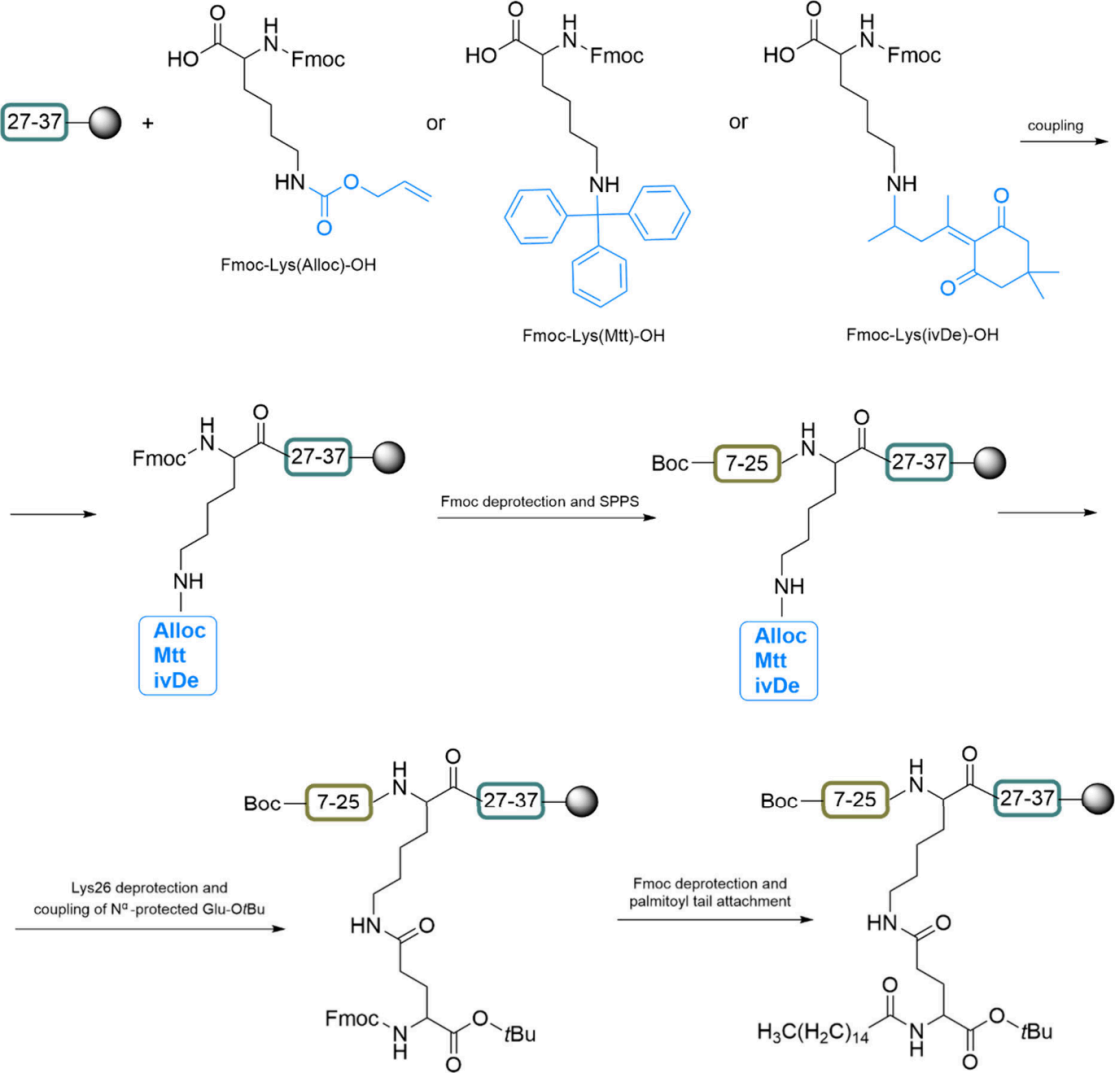
Liraglutide and Semaglutide Are Synthesized Using Orthogonal Protecting Groups such as Alloc, Mtt, or ivDde on the Lysine Residue to Enable Site-Selective Modification^a^

a Following SPPS, the protecting group on the lysine side chain is selectively removed, allowing for the subsequent coupling of additional amino acids and the attachment of a lipid tail to complete the molecule.

Another approach for liraglutide involves the solution-phase synthesis of the dipeptide Fmoc-Lys­(Pal-γ-Glu-OtBu), which contains lysine and glutamate modified with a palmitoyl chain. This dipeptide is then incorporated during SPPS. Compared to the previous method, this strategy requires fewer protecting groups. Specifically, Pal–OH reacts with H-Glu-O*t*Bu to form Pal-Glu-O*t*Bu, which subsequently reacts with Fmoc-Lys-OH, yielding the dipeptide with high purity (Scheme 12). The only protective group used in this method is O*t*Bu on the carboxyl group of glutamate.^346^

**Scheme 12:**
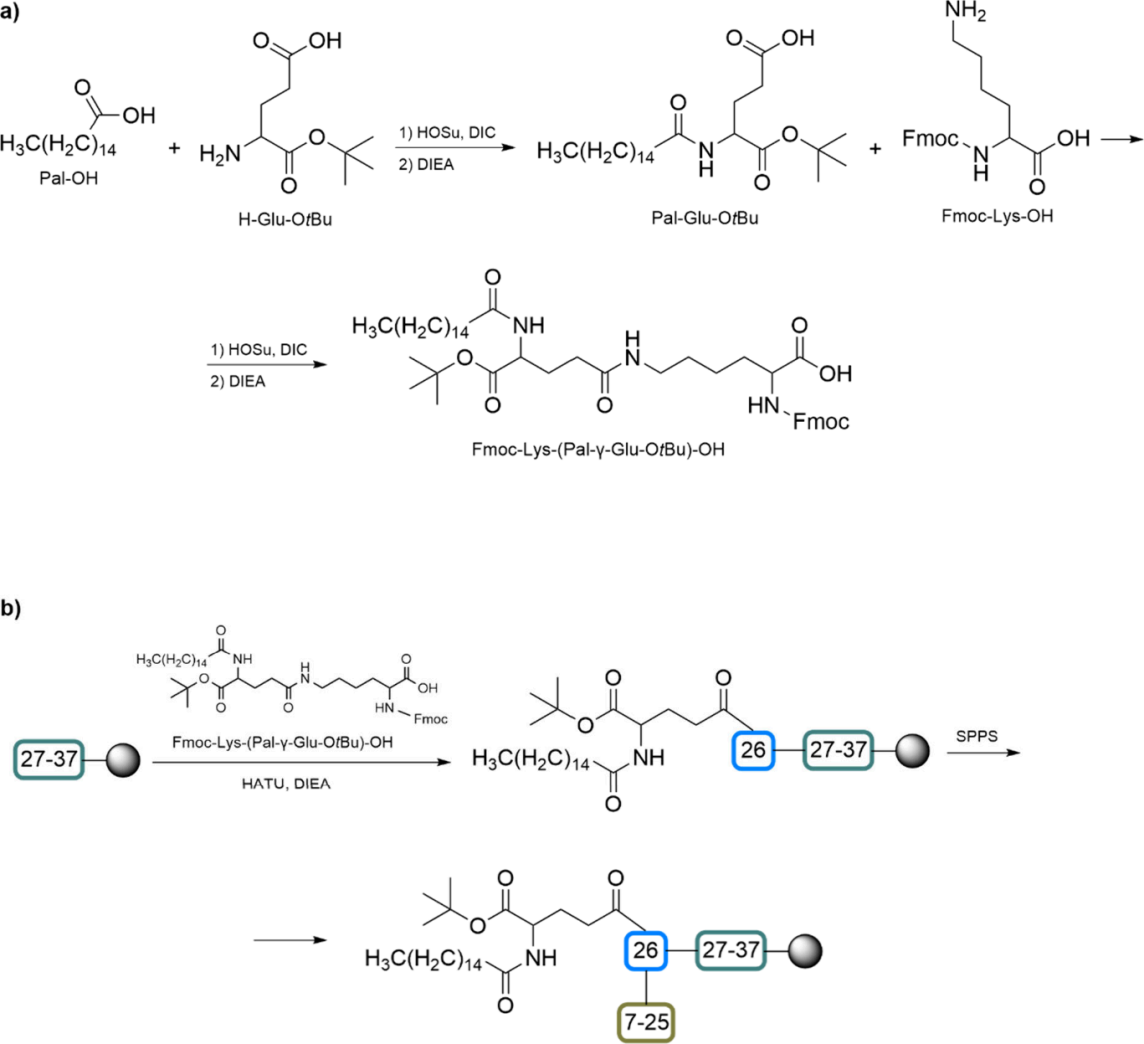
a) Synthesis of Fmoc-Lys­(Pal-γ-Glu-OtBu). b) The Solid-Phase Approach Used to Perform the Synthesis of Liraglutide Using Fmoc-Lys­(Pal-γ-Glu-OtBu)

The use of copper­(II) lysinate offers a significant simplification in the preparation of palmitoylated intermediates. Copper­(II) complexes of trifunctional amino acids, such as Lys, Asp, and Glu, can serve as temporary protecting groups, enabling the selective introduction of modifications into the side chain (Scheme 13). The potential application of copper­(II) lysinate has been explored for the synthesis of lipidated intermediate building blocks, which can subsequently be incorporated into the amino acid sequences of liraglutide and semaglutide. This strategy eliminates the need for orthogonally protected lysine as a starting material, making it particularly advantageous for the industrial-scale production of peptides.^347^

**Scheme 13:**
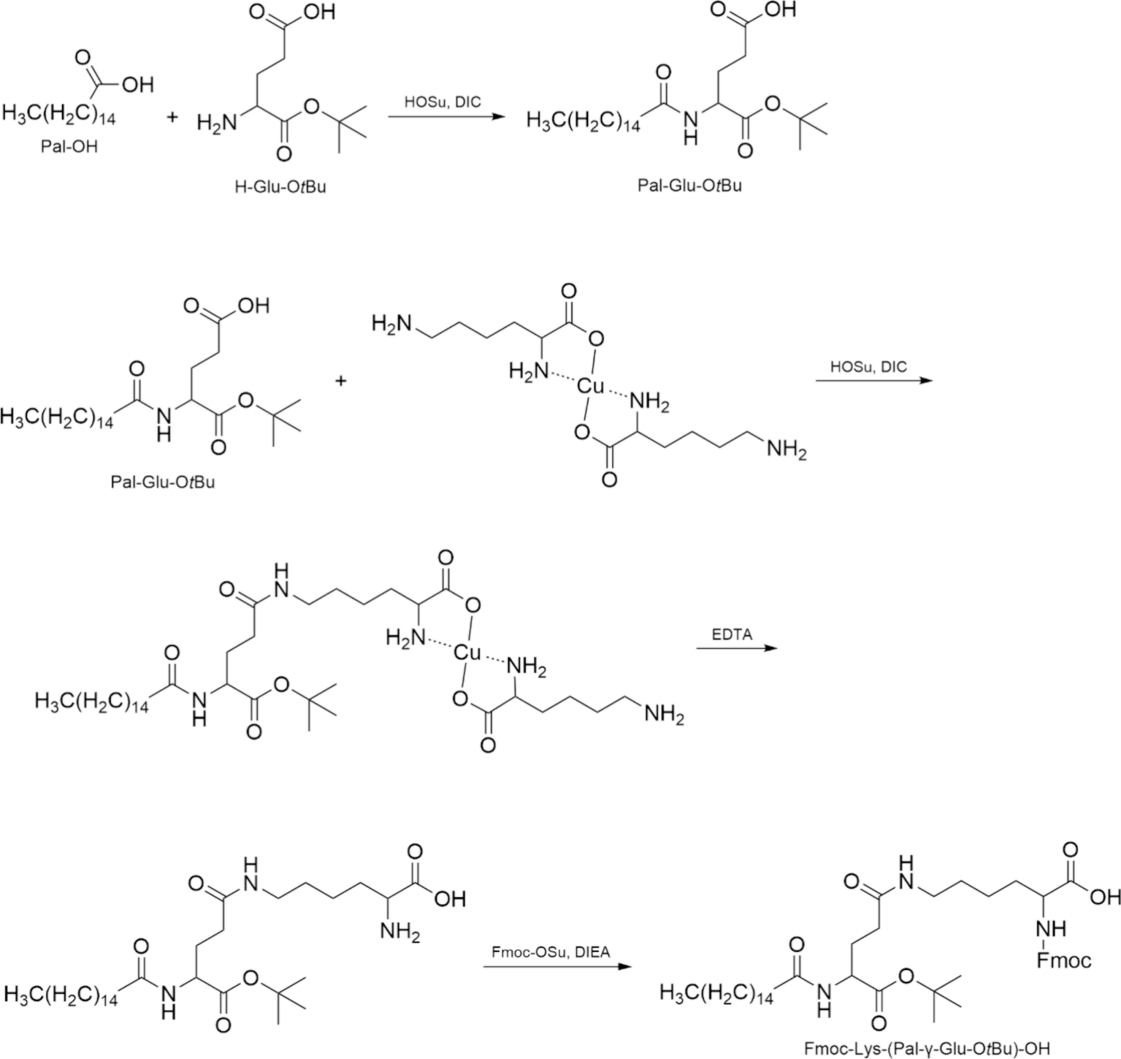
Synthesis of Fmoc-Lys­(Pal-Glu-OtBu)–OH Using Copper­(II) Lysinate As Liraglutide Intermediate^a^

a Copper­(II) coordinates with lysine, serving as a temporary protecting group. This strategy eliminates the need for pre-synthesized orthogonally protected lysine derivatives, offering a more efficient and flexible approach to site-specific modification.

Other approaches have integrated solid-phase peptide synthesis (SPPS) and liquid-phase peptide synthesis (LPPS) for the production of liraglutide. These methods involve the stepwise synthesis of peptide segments via SPPS, followed by their coupling in solution. The SPPS/LPPS hybrid approach represents a promising alternative for minimizing the formation of impurities, such as truncated sequences or peptides missing one or more amino acids. The condensation site is typically selected based on the presence of amino acids that do not undergo epimerization during coupling. These include residues such as glycine or proline at the C-terminal position of the fragments, which help maintain the stereochemical integrity of the peptide during fragment assembly. In a study conducted by the Cabri group, liraglutide was synthesized using three peptide fragments: residues 7–16, 17–24, and 27–36.^348^ The combined solid-phase/solution-phase strategy followed the assembly order: 7–16 + [17–24 + (25–36 + 37)]. However, the 7–16 segment exhibited high hydrophobicity, resulting in significant solubility issues and aggregation, making its use less efficient. A more effective strategy was found to be 7–22 + (23–36 + 37), which improved solubility and facilitated peptide assembly.

A subsequent approach involves the incorporation of a pseudoproline residue at the site of fragment condensation. Pseudoprolines are cyclic derivatives of Ser, Thr or Cys that form oxazolidine or thiazolidine rings, preventing the formation of the oxazolone intermediate, thereby reducing the risk of epimerization during peptide bond formation.^349^ Additionally, pseudoproline residues play a crucial role by effectively suppressing peptide aggregation.

In the case of liraglutide, as demonstrated by Cabri et al., a pseudoproline residue was introduced between threonine-13 and aspartic acid-15, in the place of serine-14 (Figure 19).^347^ This modification led to an almost complete conversion to the target peptide, achieving excellent purity and a high yield. However, pseudoproline residues linked to a resin via a trityl-type linker exhibit a high propensity for intramolecular cyclization, leading to diketopiperazine formation after Fmoc removal using 20% piperidine in DMF (Figure 20). The use of a piperidine/DBU/DMF mixture was explored to mitigate dipeptide detachment from the solid support. Despite this adjustment, the strategy was ineffective, resulting in a low yield of only 23%. To improve fragment solubility, 1% TritonX was added to the condensation reaction, which was successfully completed within 3.5 h. Conversely, increasing the reaction temperature to 60 °C did not yield favorable results, likely due to ester inactivation.

**Figure 20:**
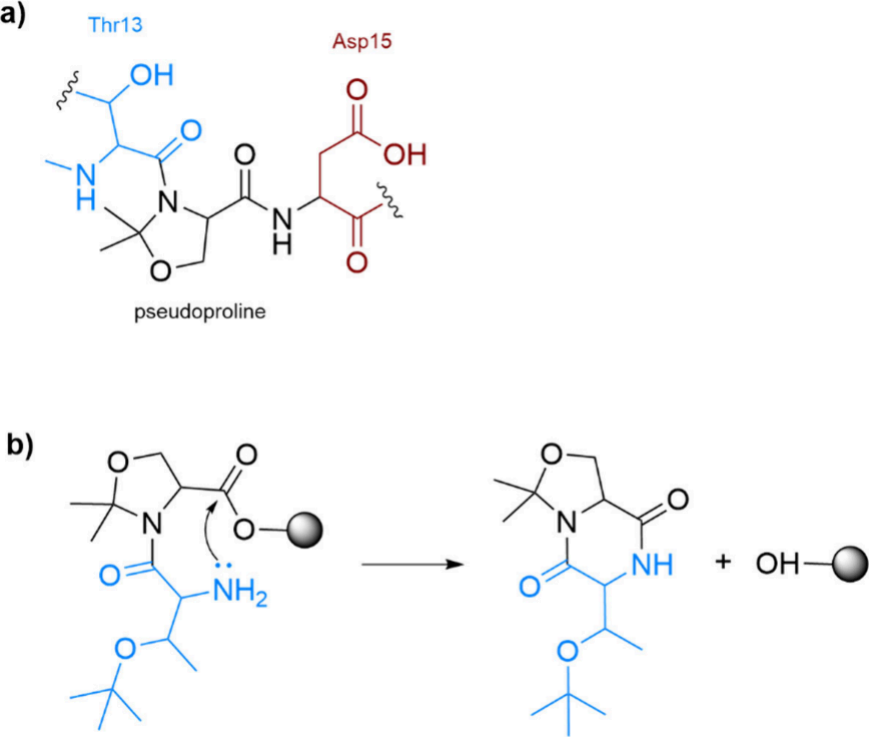
(a) The structure of the pseudoproline residue introduced between threonine-13 and aspartic acid-15, replacing serine-14. (b) The diketopiperazine (DKP) formation of the pseudoproline dipeptides on the chlorotrityl chloride (CTC) resin is depicted. During SPPS, pseudoproline residues can promote intramolecular cyclization, leading to DKP byproducts when the N-terminal amine of a growing peptide chain reacts with the carbonyl group of the adjacent pseudoproline residue, particularly if the peptide-resin attachment is labile.

The SPPS/LPPS hybrid process was initially used for the synthesis of enfuvirtide and later applied to tirzepatide.^272,350^ For the production of tirzepatide, four fragments were selected, and the disconnection points were chosen based on the potential for the epimerization of the amino acid at the C-terminal of each fragment. Each fragment was obtained with a purity of approximately 98.5% and condensate using PyOxim/*i*Pr_2_Net or HATU/*i*Pr_2_Net in DMSO/ACN (Scheme 14).

**Scheme 14:**
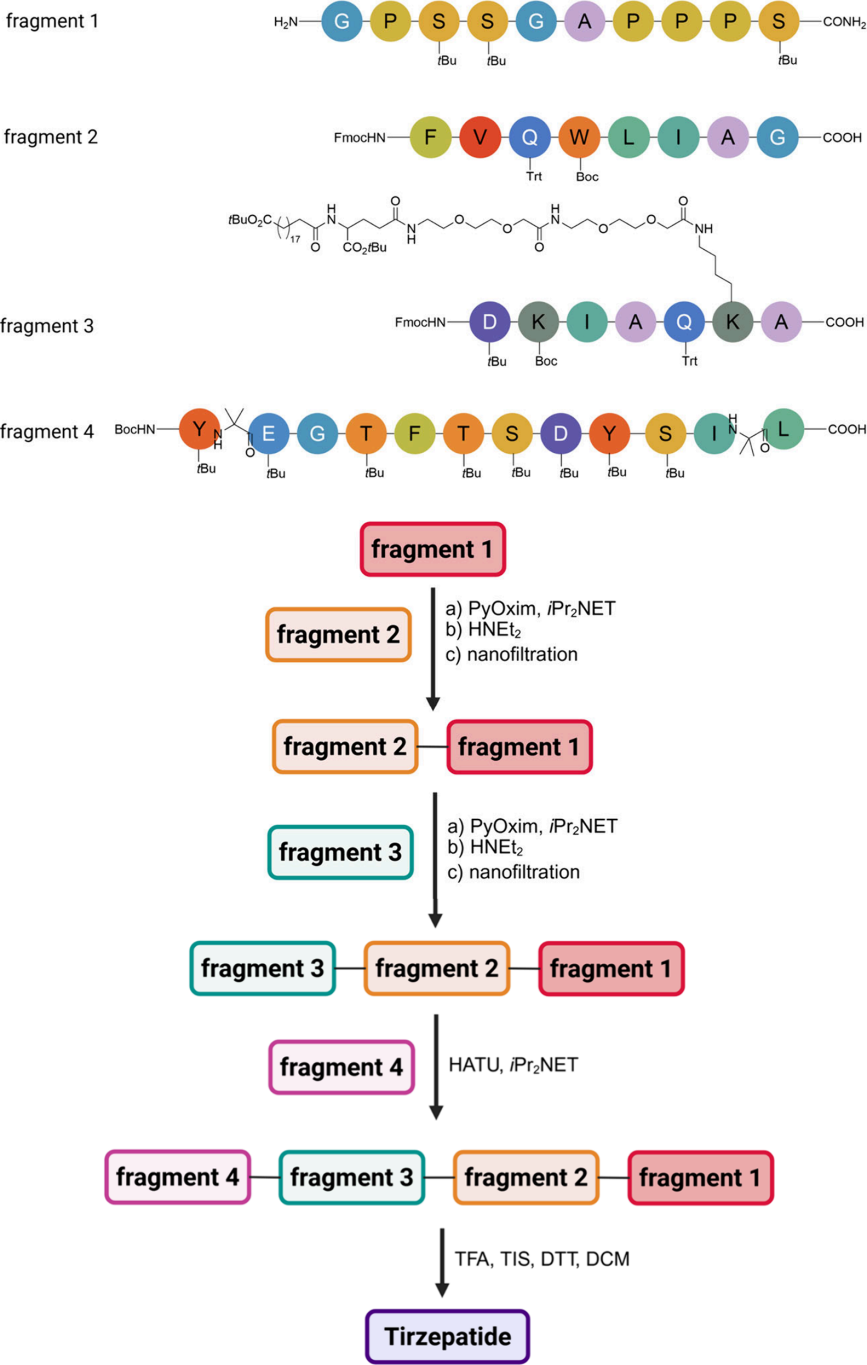
Top: Four Fragments Were Selected for the Synthesis of Tirzepatide Using a Hybrid Approach^a^ Bottom: The Scheme Details the Synthetic Method and Reagents Employed^b^

a Disconnection points were carefully chosen based on the risk of epimerization at the C-terminal amino acid of each fragment. Each fragment was synthesized with a purity of approximately 98.5% and coupled using PyOxim/iPr_2_NEt or HATU/iPr_2_NEt (also known as DIEA).
b Each fragment was used at 1 equiv, with iPr_2_NEt at 4.0 equiv and PyOxim at 1.4 equiv. The coupling reaction was carried out for 3 h at 20 °C. Following the coupling, the Fmoc protecting group was removed using 20 equiv of diethylamine (HNEt_2_) for 1 h. The final fragment was coupled using HATU (2.0 equiv) instead of PyOxim. Created in BioRender.

Lilly has also developed retatrutide, a triple agonist which is currently undergoing phase 2 clinical trials (ClinicalTrials.gov identifier: NCT04881760). Retatrutide is a single protein linked to a fatty diacid moiety that activates the GIP, GLP-1, and GCG receptors, with the GCG receptor being associated with glucagon signaling.^351^ In cell culture studies, retatrutide displayed lower potency than the natural ligands of the GCG and GLP-1 receptors (0.3 and 0.4 times as active, respectively), but exhibited significantly higher potency at the GIP receptor (8.9-fold increase). The pharmacokinetics are dose-proportional, with an estimated half-life of around 6 days, making it suitable for once-weekly subcutaneous injection. In a phase 2 study involving obese individuals without type 2 diabetes, retatrutide led to a weight reduction of up to 24.2% after 48 weeks.^352^ The treatment also improved blood pressure, lipid profiles, and glycemic control. In an additional study, the aim was to assess the mean relative change in liver fat (LF) from baseline at 24 weeks among participants with metabolic dysfunction-associated steatotic liver disease and at least 10% liver fat content. The observed mean changes in LF at 24 weeks were: – 42.9% (1 mg), – 57.0% (4 mg), – 81.4% (8 mg), – 82.4% (12 mg), and +0.3% for the placebo group. The reductions in liver fat were strongly associated with decreased body weight, abdominal fat, and improvements in metabolic indicators tied to better insulin sensitivity and lipid metabolism.

There are significant concerns about the long-term use of GLP-1RAs, particularly regarding the risk of pancreatitis and the potential development of pancreatic cancer. Animal studies have shown that exendin-4 can cause expansion of pancreatic duct glands and exacerbate chronic pancreatitis.^353^ Clinical data indicate that patients treated with exenatide had a 6-fold higher incidence of pancreatitis compared to those using other antidiabetic medications, such as rosiglitazone, nateglinide, repaglinide, and glipizide.^354^ However, other studies have found no evidence of a link between exenatide use and pancreatic injury in animal models.^355^ Due to these inconsistent findings, there is currently no definitive conclusion regarding the association between exenatide use and serious adverse pancreatic events.

Similarly, liraglutide has been associated with an increased risk of thyroid cancer in rodent studies. Long-term activation of GLP-1 receptors was observed to stimulate calcitonin secretion and induce C-cell hyperplasia, leading to a higher incidence of medullary thyroid cancer in mice.^356^ However, in human studies, liraglutide did not significantly affect calcitonin secretion.^357^ As a result, the potential correlation between GLP-1RAs and thyroid cancer remains unclear.

A frequently reported side effect of semaglutide treatment is nausea. An alternative nonpeptidic drug has been introduced for weight management: MK-801 (also known as dizocilpine), an NMDA receptor antagonist. Prolonged systemic administration of MK-801 induces anorexia and weight loss in rodents, but it is also associated with severe adverse effects, such as hyperthermia and hyperlocomotion, which have limited its clinical application.^358^ A study has shown that the conjugation of semaglutide with MK-801 can safely enhance the weight-lowering properties of this NMDA receptor antagonist. The conjugation of MK-801 to a GLP-1 analogue was achieved through a chemically cleavable reducible disulfide linker. After binding to GLP-1 receptor-expressing neurons in the brainstem and hypothalamus, the conjugate is internalized, leading to the cleavage of the linker and the release of MK-801. In mice, the glucose-lowering effect of the MK-801-semaglutide conjugate was comparable to that of semaglutide alone. At the same time, the MK-801-semaglutide combination resulted in an additional weight loss of 7%, whereas semaglutide reached a plateau. This approach demonstrates the feasibility of using peptide-mediated targeting to achieve cell-specific modulation of ionotropic receptors. It highlights the therapeutic potential of unimolecular mixed GLP-1 receptor agonism and NMDA receptor antagonism for safe and effective obesity treatment.^358^

### 6.2 Advancement in Cancer Treatment and Diagnostics

In 2022, nearly 20 million new cancer cases were recorded globally, alongside 9.7 million cancer-related deaths. Current estimates suggest that approximately one in five people will develop cancer in their lifetime, with around one in nine men and one in 12 women succumbing to the disease. Lung cancer was the most commonly diagnosed form, followed by breast cancer in women, colorectal cancer, prostate cancer, and stomach cancer.^359^

Traditional anticancer drugs, including alkylating agents, platinum-based compounds, anthracyclines, topoisomerase inhibitors, and antimicrotubule agents, often lack specificity, targeting all rapidly dividing cells rather than solely cancerous ones.^360^ This approach affects normal, healthy cells as well, leading to adverse effects such as immunosuppression, hair loss, and gastrointestinal toxicity.^361^

Advances have led to the development of monoclonal antibodies and antibody-drug conjugates (ADCs), which combine antibodies with cytotoxic drugs or radioactive particles. These targeted therapies can focus directly on tumor cells, significantly reducing toxicity. Examples of monoclonal antibodies include rituximab, trastuzumab, and bevacizumab, while ADCs include brentuximab vedotin, trastuzumab emtansine, and sacituzumab govitecan.^362^ Despite their effectiveness, these therapies face challenges related to cost, accessibility, drug stability, and immune-related side effects. In addition to antibody-based therapies, research has been exploring peptide-based approaches inspired by natural regulatory mechanisms within the body. Various peptide drugs have been developed and commercialized, including somatostatin analogues, which have shown promise in targeted cancer treatment.

Somatostatin, also known as growth hormone-inhibiting hormone (GHIH), plays an important role as a “universal inhibitor” in inhibiting the secretion of various growth hormones, such as insulin, glucagon, gastrin, secretin, and thyroid-stimulating hormones, to minimize hormone fluctuations. Somatostatin was the first human peptide produced using recombinant technology, paving the way for synthesizing complex peptides that were previously costly to produce synthetically and often induced allergic reactions when extracted from animal sources.^363^ Following the success of recombinant somatostatin, Genentech and Eli Lilly pioneered the development of the first recombinant human insulin.

Somatostatin has limited pharmacological value due to its short length and high instability, with a half-life of only 3 min. Systematic structure–activity studies identified the FWKT peptide sequence within somatostatin, representing its β-turn pharmacophore. This sequence served as a lead for developing more stable and potent analogues.^364^ Two types of somatostatin peptides occur naturally: somatostatin-14, a shorter variant with 14 amino acids, and somatostatin-28, a longer form that contains the somatostatin-14 sequence. Somatostatin-14 is mainly found in the central nervous system and pancreatic islets, where it plays a crucial role in inhibiting the release of growth hormone, insulin, glucagon, and other hormones. In contrast, somatostatin-28 is primarily located in the gastrointestinal tract and is released from intestinal cells in response to food intake; it plays a key role in regulating the digestive system by inhibiting gastrointestinal hormone release and slowing gastric emptying. Somatostatin-14 has been the primary focus for optimization and anticancer drug development.^365^ Modifications, such as truncations, incorporation of d-Phe at the N-terminus, and threonine alcohol at the C-terminus, led to the creation of the first somatostatin analogue, octreotide, for treating acromegaly, breast cancer, and prostate cancer.^366^ The enzymatic recognition site is hidden in octreotide, showing enhanced activity and stability with an extended half-life of 2 h.

Despite its relatively short sequence, the synthesis of octreotide remains challenging. One of the major difficulties is the formation of an intramolecular disulfide bond in the presence of a tryptophan residue. Additionally, the presence of a threoninol moiety at the C-terminus necessitates the use of nonconventional SPPS strategies, particularly in the choice of resin, linker and cleavage solution. An early method developed in 1991 involves the formation of a cyclic acetal in solution between the two hydroxyl groups of Fmoc-threoninol and *p*-formyl-phenoxyacetic acid. The resulting intermediate is then anchored to an aminomethyl resin, followed by peptide chain assembly on a solid phase using standard Fmoc/tBu protocols. Selective deprotection of Acm-protected cysteine residues, followed by on-resin oxidation, allows for the formation of the disulfide bridge. The peptide is subsequently cleaved from the resin using 20% TFA in DCM.^367^

Alternative strategies have since been explored, including cleavage from the resin using NaBH_4_/LiBH_4_ to yield the corresponding alcohol under reductive conditions.^368^ Another method employed HMP resin to synthesize the protected hexapeptide d-Phe-Cys­(Acm)-Phe-d-Trp­(Boc)-Lys­(Boc)-Thr­(tBu)-Cys­(Acm), which was cleaved via aminolysis using threoninol.^369^ However, these approaches generally afforded modest yields, typically not exceeding 14%.

Improved outcomes were obtained using 2-chlorotrityl resin, which provided higher yields and is also commercially available as a preloaded Thr­(tBu)-ol-2Cl-trityl resin. Disulfide bond formation was achieved on the fully deprotected peptide using either charcoal-catalyzed oxidation or an iodine solution.^370,371^ The threoninol can be linked to the 2-chlorotrityl resin through an amino group. The peptide is then synthesized on the hydroxyl group, forming an ester bond. Following cleavage, an O–N shift occurs in an aqueous solution, resulting in the formation of the threoninol and the linear form of octreotide, which is then cyclized by the formation of a disulfide bond (Scheme 15).^372^

**Scheme 15:**
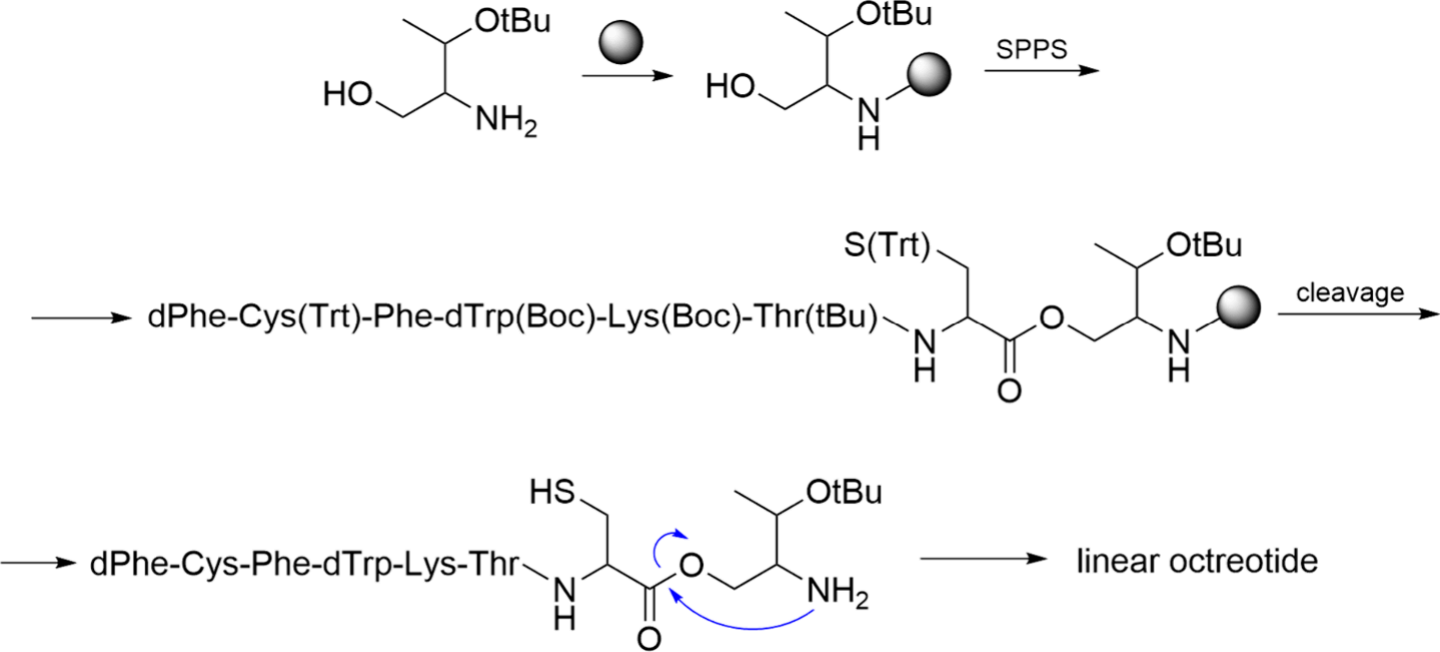
Synthesis of Octreotide via Threoninol Formation on 2-Chlorotrityl Resin^a^

a A β-amino alcohol is initially anchored to the 2-chlorotrityl chloride (2-CTC) resin. Peptide elongation is then performed on the hydroxyl group, resulting in the formation of an ester bond. Upon cleavage from the resin, an O→N acyl shift occurs in aqueous solution, leading to the generation of the threoninol moiety and the linear form of octreotide. Subsequently, the peptide is cyclized through disulfide bond formation to yield the final cyclic structure.

Other resins, such as Rink amide, have also been employed. In one approach, threoninol was introduced as N-Boc-O-Bzl-threoninol on a succinimidyl carbonate resin, with peptide synthesis carried out via the Boc/Bzl strategy. To enable the use of the Fmoc/tBu approach, a novel acid-labile linker was developed by condensing the two hydroxyl groups of N-protected threoninol with the aldehyde group of *p*-carboxybenzaldehyde. This linker can be anchored to Rink amide resin, allowing synthesis via the Fmoc/tBu strategy.^373–375^ After full cleavage, disulfide bond formation was accomplished via air oxidation over 48 h in a dilute (ca. 1 mM) ammonium acetate/ammonium hydroxide buffer.

At the industrial level, octreotide synthesis has focused on solution-phase coupling of peptide fragments. The strategy disclosed in several patents involves the preparation of two tripeptides (Boc-d-Phe-Cys­(Acm)-Phe-OMe and Z-d-Trp-Lys­(Boc)-Thr-OMe) and one dipeptide (H-Cys­(Acm)-Thr-OMe or -ol), followed by methyl ester hydrolysis and fragment condensation according to the [3 + 3] + 2 scheme.^376,377^ A refinement of this approach involved coupling the C-terminal dipeptide alcohol H-Cys­(Acm)-Thr-ol to a hexapeptide intermediate, Boc-d-Phe-Cys­(Acm)-Phe-d-Trp-Lys­(Boc)-Thr-OH, synthesized from a dipeptide and a tetrapeptide. This strategy avoids racemization of the phenylalanine residue at position 3.^378^ Final disulfide bond formation was conducted after complete deprotection using hydrogen peroxide.

Furthermore, five distinct subtypes of somatostatin receptors (SSTR1 to SSTR5) have been identified, with one subtype predominantly overexpressed in tumors. This discovery prompted the development of receptor-selective somatostatin analogues, including lanreotide, vapreotide, and pasireotide.^379^ In pasireotide, cyclization between the N- and C-termini extends its half-life to 12 h, making it particularly effective for treating Cushing’s disease compared to other somatostatin analogues (Figure 21, Table 2).

**Figure 21:**
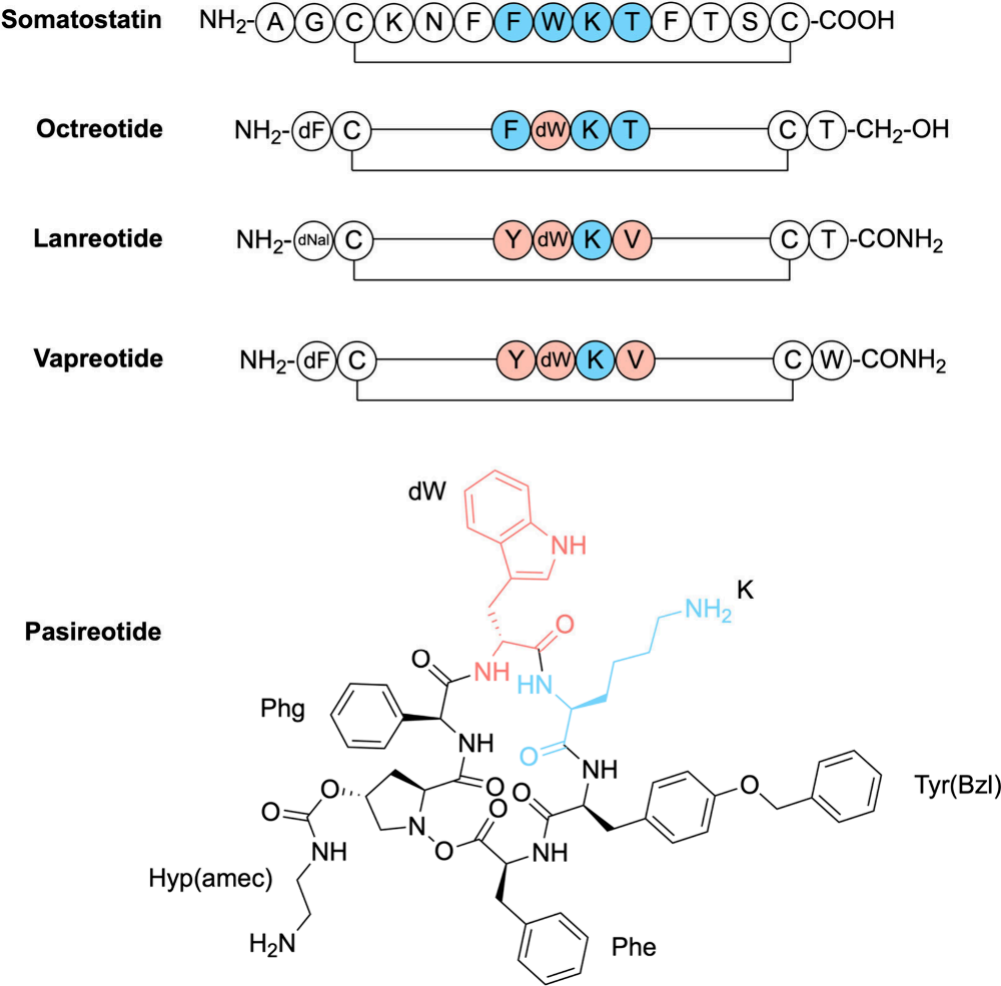
On the top is the sequence of the native somatostatin peptide. The blue sequence in somatostatin represents a key pharmacophore in somatostatin, responsible for receptor binding. In somatostatin analogues, lysine (K) is conserved, while tryptophan is replaced with its stereoisomers in octreotide, lanreotide, vapreotide, and pasireotide. Phenylalanine is substituted with tyrosine, which has an additional hydroxyl group, and threonine is replaced with a more hydrophobic amino acid, such as valine, in lanreotide and vapreotide. Pasireotide incorporates the highest number of unnatural amino acids. In this analogue, cyclization occurs between the N- and C-terminal ends, while in octreotide, lanreotide, and vapreotide, cyclization involves the side chains of cysteine residues, similar to native somatostatin.

**Table 2:** Peptide Drugs Launched on the Market to Treat Cancer and Fertility

Generic name	Brand name	Drug class	FDA first approval year	Company	Therapeutic indication	Route^a^
octreotide	Sandostatin, Octreotide Acetate	somatostatin analogue (SSA)	1988	Abraxis, Bedford Laboratories, Sandoz-Novartis, Sun, Teva	acromegaly, neuroendocrine tumors, and conditions with excess hormone secretion	SC, IV
octreotide	Sandostatin LAR	somatostatin analogue (SSA)	1998	Novartis	acromegaly, neuroendocrine tumors, and conditions with excess hormone secretion	SC
octreotide	Mycapssa	somatostatin analogue (SSA)	2020	Chiasma	acromegaly, neuroendocrine tumors, and conditions with excess hormone secretion	O
octreotide	Bynfezia pen	somatostatin analogue (SSA)	2024	Sun Pharma	acromegaly, neuroendocrine tumors, and conditions with excess hormone secretion	SC
lanreotide	Somatuline	somatostatin analogue (SSA)	2007	Ipsen, Globopharm, Tercica	acromegaly, neuroendocrine tumors, and conditions with excess hormone secretion	SC
lanreotide	Somatuline depot	somatostatin analogue (SSA)	2007	Ipsen	acromegaly, neuroendocrine tumors, and conditions with excess hormone secretion	SC
pasireotide	Signifor	somatostatin analogue (SSA)	2012	Novartis	Cushing’s disease and acromegaly	SC
pasireotide	Signifor LAR	somatostatin analogue (SSA)	2014	Novartis	Cushing’s disease and acromegaly	IM
vapreotide	Octastatin, Sanvar	somatostatin analogue (SSA)	not yet	Debiovision	acute esophageal variceal bleeding	IV
gonadorelin	Factrel, Kryptocur, Lutrelef, Lutrepulse, Relefact, Stimu-LH	GnRH agonist	1982	Baxter Healthcare, Ferring, Wyeth, Sanofi-Aventis	Evaluation of hypothalamus and pituitary gland function, infertility, central precocious puberty	SC
leuprolide	Camcevi, Enantone, Eligard, Fensolvi, Lupron Depot, Lupron Depot-Ped, Lupron, Lupron Depot-Gyn, Viadur	GnRH agonist	1985	Abbott, Alza, Astellas, Bayer, Bedford Laboratories, Genzyme, Johnson & Johnson, QLT, Sanofi-Aventis, Takeda, Teva, Wyeth	prostate cancer, endometriosis, and precocious puberty	SC
buserelin	Bigonist, Suprefact, CinnaFact, Metrelef, Suprecur	GnRH agonist	1988	Sanofi-Aventis, CinnaGen, Ferring	prostate cancer, endometriosis, and infertility	SC
goserelin	Zoladex	GnRH agonist	1989	AstraZeneca	prostate cancer, breast cancer, and endometriosis	SC
nafarelin	Synarel, Synrelina	GnRH agonist	1990	Pfizer, Searle	endometriosis	SC
histrelin	Supprelin, Supprelin La, Vantas	GnRH agonist	1991	Endo, Roberts, Shire	prostate cancer	SC
triptorelin	Decapeptyl, Diphereline, Gonapeptyl, Pamorelin, Trelstar	GnRH agonist	2000	Debiopharm, Ferring, Ipsen, Watson	prostate cancer	SC
ganirelix	Ganirelix Acetate, Antagon, Fyremadel, Orgalutran	GnRH antagonists	1999	Organon	prevent premature ovulation in fertility treatments	SC
cetrorelix	Cetrotide	GnRH antagonists	2000	Aeterna Zentaris, Merck-Serono	control ovulation timing	SC
abarelix	Plenaxis	GnRH antagonists	2003	Praecis, Specialty, European Pharma	advanced prostate cancer	SC
degarelix	Firmagon, Degareli Acetate	GnRH antagonists	2008	Ferring, Astellas	advanced prostate cancer	SC
romidepsin	Istodax	histone deacetylase inhibitors	2004	Bristol-Myers, Teva	cutaneous T-cell lymphoma	IV
secretin	Chirhostim	in vivo diagnostic biologicals	2004	Chirhoclin	diagnosis of tomour in the pancreas or bowel	IV
carfilzomib	Kyprolis	proteasome inhibitors	2012	Onyx Pharms Amgen	multiple myeloma	IV

a SC: subcutaneous, IV: intravenous, O: orally, IM: intramuscular.

Due to their ability to bind to receptors that are overexpressed in specific tumor types, a range of somatostatin analogues have been developed for diagnostic purposes. Diagnostic somatostatin analogues (Table 3), including In-111 pentetreotide, Ga-68 DOTA-TATE, Ga-68 DOTA-TOC and Cu-64 DOTA-TATE, are employed for diagnostic and therapeutic purposes.^380–383^ These analogues consist of the peptide octreotide linked to a radioactive tracer via N-terminal chelation, which enables precise tumor detection and treatment. Through techniques such as peptide scintigraphy, targeted radiotherapy, computed tomography (CT) and positron emission tomography (PET), these radiolabeled peptides facilitate the visualization of somatostatin receptor-expressing tumors, enhancing diagnostic accuracy and therapeutic efficacy.^384^ Lu-177 DOTA-TATE is another approved metal-containing peptidomimetic and is used as a therapeutic isotope.^385^

**Table 3:** Peptide-Based Radiopharmaceuticals Approved by the FDA

Generic name	Brand name	Drug class	FDA first approval year	Company	Therapeutic indication	Route^a^
In111 pentetreotide	Octreoscan	radiotracer for scintigraphy	1994	Mallinckrodt	assessment of neuroendocrine tumors	IV
Depreotide	Noetect	radiotracer for scintigraphy	1999	Diatide	PET imaging to detect and stage somatostatin receptor-positive neuroendocrine tumors in pulmonary masses	IV
Ga68 DOTA-TATE	Netspot	radiotracer for PET imaging	2016	Advanced Accelerator Applications	PET imaging to detect and stage somatostatin receptor-positive neuroendocrine tumors	IV
Lu177 DOTA-TATE	Lutathera	radiotherapeutic agent	2018	Advanced Accelerator Applications	somatostatin receptor-positive neuroendocrine tumors	IV
Ga68 DOTA-TOC	SomaKit TOC	radiotracer for PET imaging	2019	Advanced Accelerator Applications	PET imaging to detect and stage somatostatin receptor-positive neuroendocrine tumors	IV
Ga68 PSMA-11	Illuccix	radiotracer for PET imaging	2020	Telix Pharmaceuticals	PET imaging to detect prostate cancer	IV
Cu64 DOTA-TATE	Detectnet	radiotracer for PET imaging	2020	RadioMedix	PET imaging to detect and stage somatostatin receptor-positive neuroendocrine tumors	IV
piflufolastat F 18	Pylarify	radiotracer for PET imaging	2021	Progenics Pharmaceuticals	detecting prostate-specific membrane antigen	IV
Lu 177 vipivotide tetraxetan	Pluvicto	radiotherapeutic agent	2022	Novartis	metastatic castration-resistant prostate cancer	IV
gadopiclenol	Elucirem	radiotracer for MRI	2022	Guerbet	enhance image clarity, particularly in detecting lesions in the brain, spine, liver, and other soft tissues	IV
Flotufolastat F-18	Posluma	radiotracer for PET imaging	2023	Blue Earth Diagnostics	PET imaging in prostate cancer to detect prostate-specific membrane antigen	IV
Pegulicianine	Lumisight	fluorescence imaging agent	2024	Lumicell	identify cancerous tissue during surgery	IV

a IV: intravenous.

The Ga-68-PSMA-11 complex represents another important diagnostic peptide. Unlike the somatostatin analogues mentioned above, it is a urea-based peptidomimetic that has become an essential tool in prostate cancer diagnosis. Prostate-Specific Membrane Antigen (PSMA) has emerged as a key biomarker and therapeutic target in oncology, particularly for prostate cancer. PSMA-11 is a peptidomimetic in which the Glu-Urea-Lys sequence enables selective binding to PSMA.^386^ Conjugation of PSMA-11 with the radioactive isotope Ga-68 enables its application in PET/CT imaging.^387^ The resulting 68Ga-PSMA-11 complex offers high sensitivity and specificity for the detection of metastatic or recurrent prostate cancer, playing a crucial role in diagnosis, staging, and monitoring.^388^ It has transformed prostate cancer imaging by allowing the identification of smaller lesions that may evade detection by conventional imaging techniques. Furthermore, 68Ga-PSMA-11 provides superior accuracy, yielding clearer and more reliable images of malignant tissues, and has become an integral component of modern prostate cancer management.

All somatostatin analogues primarily act as agonists; however, certain peptide drugs have been developed as antagonists, particularly those that mimic gonadotropin-releasing hormone (GnRH) (Table 2). GnRH stimulates the release of follicle-stimulating hormone (FSH) and luteinizing hormone (LH), with LH playing a crucial role in initiating ovulation during the menstrual cycle. In situations where an egg is released prematurely before it is ready for fertilization, GnRH antagonists function by binding to GnRH receptors to inhibit the activity of the natural GnRH and preventing the premature release of the egg, thereby facilitating reproductive management. Notable examples of GnRH antagonists include cetrolix, ganirelix.^389,390^ Additionally, degarelix, and abarelix are effective GnRH antagonists used in the treatment of advanced prostate cancer (Figure 22).^391,392^

**Figure 22:**
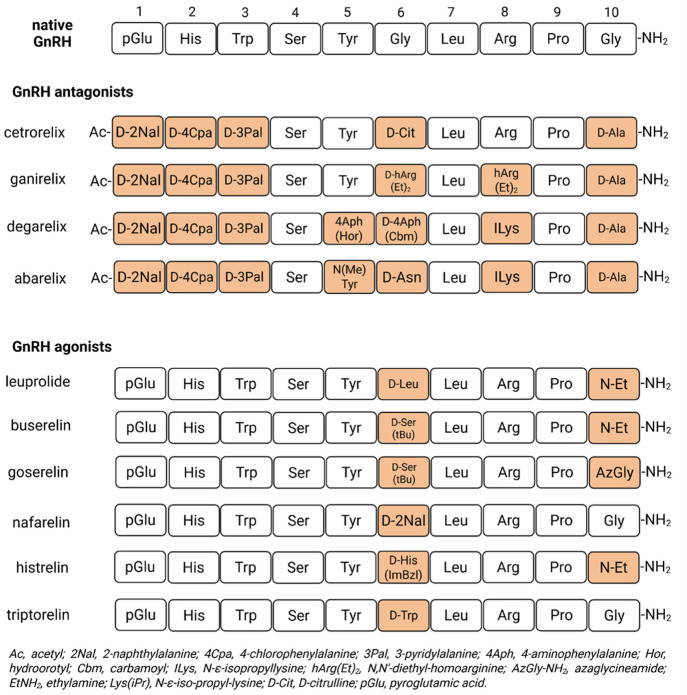
At the top is the sequence of the native GnRH peptide, followed by various commercially available analogues. Residues differing from the original sequence are highlighted in orange, showcasing the extensive use of unnatural amino acids. For such short sequences, numerous modifications are necessary to ensure a sufficiently prolonged half-life for these peptide drugs. Created in BioRender.

In these antagonists, all positions have been substituted with D-amino acids or unnatural amino acids, except for positions 4, 7, and 9, which remain unchanged from the original sequence.

Conversely, other drugs have been developed as superagonists of GnRH, which serve to desensitize and downregulate GnRH receptors, indirectly exerting their antagonistic effects. The GnRH superagonists include leuprolide, buserelin, goserelin, nafarelin, histrelin, and triptorelin.^393–398^ These agents are clinically applied in cancer treatment, puberty suppression, management of estrogen-dependent female disorders, sex reassignment, and in vitro fertilization therapy. Superagonists have been designed by substituting the glycine at position 6 with a D-stereoisomer amino acid. This modification enhances the peptide stability against proteolytic degradation and increases their receptor affinity. In the native peptide, C-terminal amidation is present to confer resistance to carboxypeptidase degradation. In contrast, superagonists incorporate ethylamine or a hydrazine glycine mimetic, which protects against carboxypeptidase similarly to the original amidation while also increasing hydrophobicity (ethylamine) and conformational rigidity (hydrazine glycine mimetic), thereby enhancing potency and duration of action. Unlike GnRH antagonists, the superagonists feature fewer modifications, with alterations primarily observed at positions 6 and 10.

Several methods have been described for the synthesis of peptide *N*-alkyl amides, such as leuprolide. These methods rely on nucleophilic displacement of the peptide, which is anchored to a Merrifield-type resin, oxime resin, or polyacrylic resin.^399,400^ Standard features of these approaches include: (i) elongation of the peptide chain using either Boc or Fmoc chemistry, (ii) cleavage of the peptide from the resin via a nucleophile, and (iii) side-chain deprotection using either HF or TFA, depending on the strategy employed in step (i).

In the case of leuprolide, synthesis was performed on a Merrifield-like resin using Boc chemistry. The protected peptide was cleaved from the polymeric support using MeOH/TEA, followed by treatment of the resulting ester with ethylamine.^401^ To enable the use of the Fmoc strategy, leuprolide was then synthesized on a hydroxymethyl-Nbb resin, which can be prepared from MBHA resin and 4-hydroxymethyl-3-nitrobenzoic acid using alanine as an internal standard.^402^ The first four amino acids were coupled using Boc chemistry to avoid the formation of diketopiperazine (DKP), after which the synthesis was continued using the Fmoc strategy (Scheme 16).

**Scheme 16:**
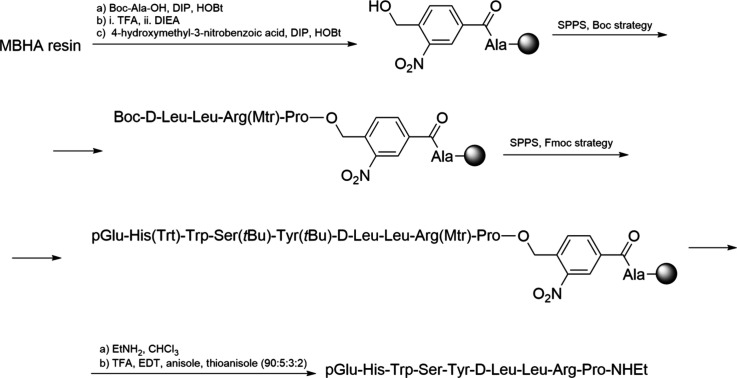
Leuprolide Was Synthesized Using the Fmoc-Based SPPS on a Hydroxymethyl-Nbb Resin, Prepared from MBHA Resin and 4-Hydroxymethyl-3-nitrobenzoic Acid, with Alanine Employed as an Internal Standard^a^

a To prevent DKP formation, the first four amino acids were coupled using Boc chemistry. Following this initial step, peptide chain elongation proceeded using the Fmoc strategy. The final cleavage was carried out using a standard cocktail of TFA.

Other FDA-approved peptide-based drugs with antitumoral effects include carfilzomib and romidepsin. Carfilzomib, approved in 2012, is indicated for treating multiple myeloma in patients who have received at least two prior therapies, including bortezomib and an immunomodulatory agent, but continue to exhibit disease progression within 60 days of their last treatment.^403^ This drug is a tetrapeptide derived from the natural products epoxomicin and eponemycin, which exhibit antitumor activity. Carfilzomib selectively inhibits the chymotrypsin-like (CT-L) activity of the 20S proteasome via an epoxyketone moiety at the C-terminus, allowing irreversible binding to the CT-L site. This mechanism differentiates it from bortezomib. This inhibition results in the accumulation of polyubiquitinated proteins, leading to cell cycle arrest, apoptosis, and suppression of tumor growth. Carfilzomib has a short half-life of approximately 30 min and is primarily cleared through biliary and renal excretion, a characteristic that may contribute to its favorable safety profile.

Romidepsin is an anticancer agent that exerts its effects through chromatin remodelling, specifically as a histone deacetylase (HDAC) inhibitor. Approved by the FDA in 2009 for treating cutaneous T-cell lymphoma, a rare form of non-Hodgkin lymphoma, romidepsin is a bicyclic pentapeptide with both N-to-C terminal cyclization and a disulfide bond. It was originally isolated from *Chromobacterium violaceum*, a Gram-negative bacterium sourced from Japanese soil.^404^ The structure of romidepsin comprises d-Val, DCys, Z-dehydrobutyrine, l-Val, and (3S,4E)-3-hydroxy-7-mercapto-4-heptenoic acid. Romidepsin functions as a prodrug, activated intracellularly through disulfide bond reduction by glutathione. The released free thiols coordinate with zinc ions within the active sites of class I and II zinc-dependent HDAC enzymes, leading to enzyme inhibition. In its active form, romidepsin is rapidly inactivated in serum, with an approximate half-life of 3 h. It is commercially produced through fermentation.

In addition to drugs that mimic natural hormone peptides, substantial research is directed toward developing peptides capable of interfering with protein–protein interactions. Significant advancements have been achieved, but further investigation is needed before effective peptide-based drugs can be developed to interfere with the tumor pathways that regulate cellular proliferation. Below, we highlight some of the most notable examples.

Grb7, a crucial protein associated with cancer cell proliferation and migration, is a noteworthy target of interest, particularly in breast cancer subtypes. The main interaction of Grb7 occurs with its upstream signaling partners via its Src homology 2 (SH2) domain, which results in Grb7 tyrosine phosphorylation and subsequent signal transduction. Consequently, there is a hypothesis suggesting that inhibiting the Grb7-SH2 domain could potentially impede breast cancer cell migration, along with other signal transduction pathways associated with Grb7-SH2. Inhibiting Grb7 has the potential to enhance the effectiveness of anticancer treatments. G7–18NATE, an 11-residue peptide (WFEGYDNTFPC), cyclized via a thioether bond from the N-terminus to the C-terminal thiol side chain of cysteine, exhibits robust binding to Grb7-SH2 under specific conditions.^405^ Notably, this binding is phosphate-dependent, with the presence of phosphate stabilizing the interaction and its absence resulting in reduced affinity and specificity. The Grb7-SH2 domain plays a critical role as an interaction site, especially for peptides featuring a pYXN motif in a turn conformation, where pY represents phosphotyrosine. Researchers have explored pY mimetics to enhance Grb7 inhibitors, motivated by concerns about the impact of the phosphate group on membrane permeability and stability. Carboxylic acid–based pY mimetics, such as carboxymethylphenylalanine (cmF) and carboxyphenylalanine (cF), have demonstrated promising binding to Grb7-SH2 under physiological conditions (Figure 23).^406^

**Figure 23:**
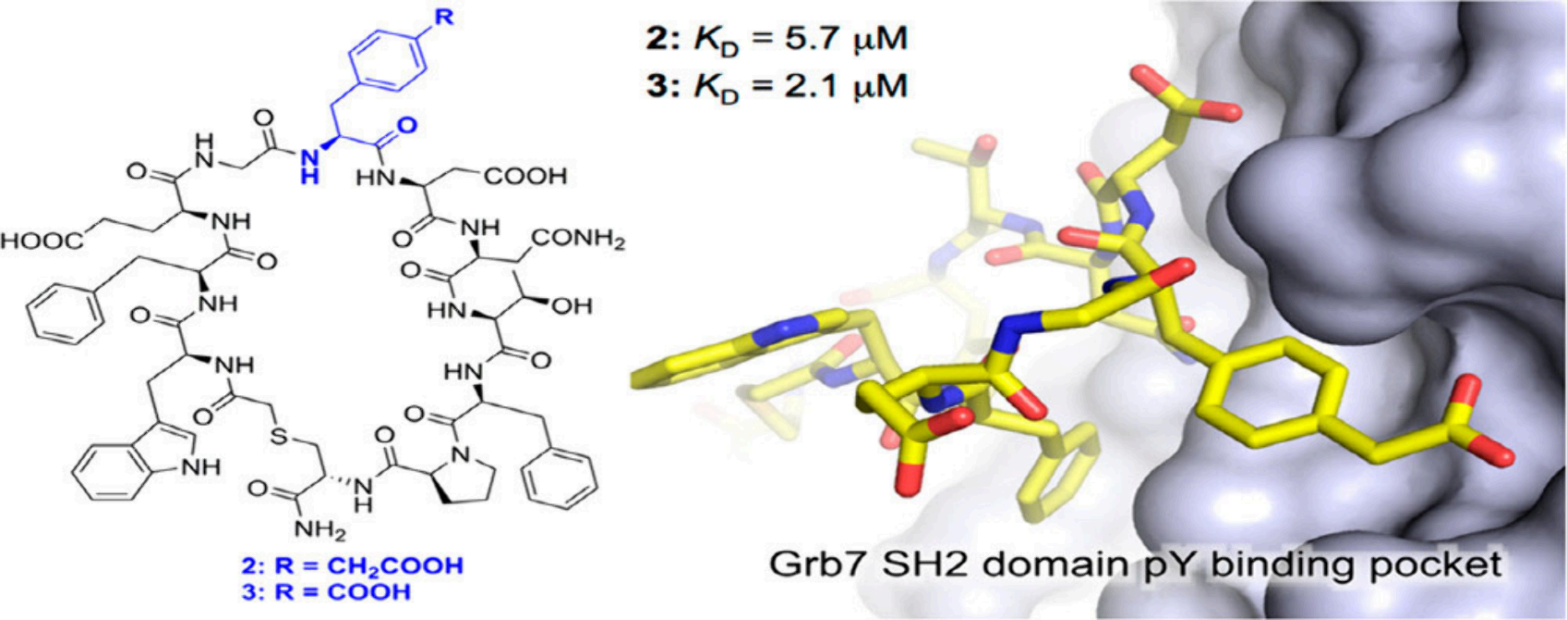
G7–18NATE is an 11-residue peptide cyclized through a thioether bond between the N-terminus and the thiol side chain of the C-terminal cysteine. This construct exhibits robust binding affinity for the Grb7-SH2 domain under specific conditions. Peptidomimetics derived from G7–18NATE, incorporating carboxymethylphenylalanine (cmF) or carboxyphenylalanine (cF)—represented as the blue amino acid in the figure—have demonstrated enhanced binding to Grb7-SH2 under physiological conditions. Reproduced with permission from ref 406. Copyright 2015, American Chemical Society.

The initial success of G7–18NATE spurred the development of a series of second-generation Grb7-SH2 inhibitors, resulting in anenhancement in affinity for the interaction with Grb7-SH2. These enhancements included the addition of a covalent tether to create a bicyclic peptide, removal of two unnecessary amino acids at positions 9 and 10, and incorporation of phosphotyrosine mimetics. This led to the creation of a nine-amino-acid bicyclic peptide scaffold named G7-B7, with a K_D_ of 0.27 μM. Despite its higher affinity for Grb7-SH2 in vivo, G7-B7 exhibited lower activity than its predecessor, G7–18NATE, in vitro.^407^

Small GTPases, including Ras, Rab, and Rho, play crucial roles in various cancer types, where their dysfunction contributes to abnormal cell growth and differentiation, prolonged cell survival, disturbed membrane trafficking, and impaired vesicular transport. Targeting the activity of these small GTPases presents an opportunity for developing innovative chemotherapeutic agents in cancer treatment. A viable strategy to pursue this objective involves addressing the GDP-GTP exchange process in Ras, a rate-limiting step dependent on the interaction with the Ras-specific guanine nucleotide exchange factor Sos. Inhibition of Sos-mediated Ras activation is a promising strategy for experimental and therapeutic intervention. Structural analyses revealed the involvement of multiple interactions in Ras-Sos interactions, particularly the insertion of a helical hairpin from Sos into Ras switch regions. Computational and experimental analyses identified key residues for helix binding to Ras.^408^ Stabilized helices mimicking the full-length Sos αH helix were designed using the hydrogen bond surrogate (HBS) approach. HBS helices, preorganized and targeting specific protein receptors, were chosen for their high affinity and specificity. Optimization of the helical mimic sequence enhanced solubility and inhibitory potential against Ras-Sos association. The resulting optimized sequence, FEGIYRLELLKAEEAN, showed promise as a synthetic mimic of the Sos αH helix.

Stapled peptides have been investigated for their potential as cancer inhibitors, exemplified by their application in the PPI between the tumor suppressor p53 and its negative regulator MDM2 and MDMX.^409^ This interaction has garnered extensive research attention and has made significant progress in clinical development. P53, recognized as the guardian of the genome, is a crucial transcription factor responsible for regulating processes such as cell cycle arrest, apoptosis, and cellular senescence. The functional significance of p53 is underscored by somatic mutations that deactivate p53 in up to 50% of human cancers. In the remaining cases, functional p53 is often hindered by negative regulators acting through post-translational modifications or protein sequestration. Consequently, extensive efforts have been directed toward overcoming these regulatory challenges and harnessing p53 tumor suppressor capabilities to induce cell death. Despite several classes of compounds reaching clinical trials, questions persist regarding their toxicity, off-target effects, and how to effectively address mutational resistance.^410^ To tackle these challenges, researchers have explored a diverse array of peptidomimetics, including peptide hybrids, achiral peptoids, oligobenzamides, and foldamers.^411–413^ Additionally, initiatives have been undertaken to create high-affinity peptide ligands through phage display technologies.^414^ Among these innovative modalities, stapled peptide-based inhibitors have made significant strides in clinical development, serving as a platform for testing inventive staple architectures and chemical approaches, particularly those designed using the 1-CPS and 2-CPS methods to generate inhibitors for the p53-MDM2 interaction.^415–417^ The one-component (1-CPS) and two-component (2-CPS) thiol–ene reactions have proven effective in producing stapled peptides targeting MDM2.^418^

### 6.3 Combating Viral Infections

Given the considerable genetic variability observed in viruses like human immunodeficiency virus 1 (HIV-1), hepatitis C virus (HCV), and SARS-CoV, the rapid development of drug resistance has become a pressing concern. As a result, research on antiviral peptides is relatively limited, with only a few examples reported in the literature.

Strategies to create antiviral drugs focus on impeding viral entry into host cells, a goal that can be achieved by employing peptides designed to mimic the binding sites of crucial proteins involved in the entry process. For example, HIV-1 relies on its viral envelope trimeric protein, gp120, to initiate binding with the CD4 protein, facilitating its access to host T-cells. This interaction induces a structural transformation in gp120, enabling the virus to subsequently attach to coreceptors expressed on the host cell, specifically the chemokine receptors CCR5 or CXCR4.^419^ Subsequent to this engagement, a structural alteration in another viral trimeric protein, gp41, triggers the insertion of a fusion peptide into the host cell membrane, facilitating the merging of the virus’s outer membrane with the cell membrane (Figure 24a).

**Figure 24:**
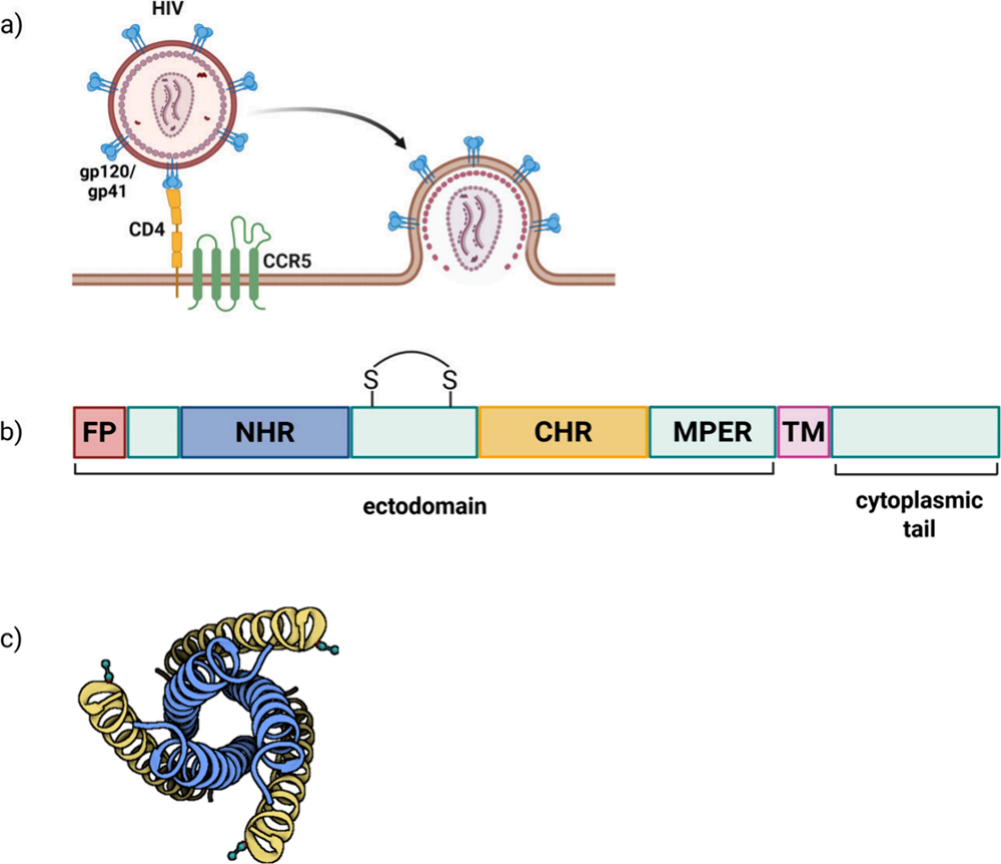
(a) HIV is an enveloped virus that requires fusion of its lipid membrane with the target cell membrane to initiate infection. This process is mediated by glycoproteins. Specifically, the gp120 glycoprotein binds to the CD4 receptor on T-cells. This interaction induces conformational changes in gp120, enabling it to bind to chemokine receptors such as CCR5 or CXCR4. Following this, gp41 inserts its fusion peptide into the target cell membrane, initiating the membrane fusion process. (b) Gp41 can be structurally divided into three main domains: the ectodomain, the transmembrane (TM) region, and the cytoplasmic tail. The ectodomain contains several distinct functional regions that play critical roles in membrane fusion and viral infectivity. At the N-terminus, a hydrophobic segment known as the fusion peptide (FP) is followed by an α-helical region termed the N-heptad repeat (NHR). A disulfide-bridged loop connects the NHR to a C-terminal helical region (CHR). The CHR is in turn linked to the TM domain by a conformationally flexible region known as the membrane-proximal external region (MPER). (c) The postfusion structure of the gp41 core is characterized by a six-helix bundle formed by the NHR and CHR regions (PDB: 1AIK). At the center of this bundle is a parallel, trimeric coiled coil composed of three NHR helices arranged in a left-handed superhelix. Surrounding this core, three CHR helices are oriented antiparallel to the NHR helices and wrap around the outside of the central coiled-coil trimer. Created in BioRender.

Initial research focuses on exploring peptides that replicate segments of gp41 (Figure 24b). Specifically, considerable attention has been directed toward peptides emulating the six-helix bundle configuration formed by the helical structures located at the N-terminal (NHR) and C-terminal (CHR) regions (Figure 24c). The formation of this six-helix bundle in gp41 holds significant importance in the fusion process between viral and cellular membranes. Peptides presenting segments of this six-helical bundle are believed to harbor the potential to disrupt its assembly, thereby hindering the fusion of the virus with host cells. A study conducted by Wild and colleagues has elucidated the antiviral activity of a helical peptide mimicking the NHR, demonstrating efficacy against HIV-1.^420^ Following this, it was noted that trimeric formations of the NHR-mimetic peptide displayed enhanced inhibition of HIV-1 entry when compared with their monomeric counterparts. The incorporation of covalent stabilization within these peptide trimers, accomplished by forming interchain disulfide bridges, led to a significant augmentation of their antiviral efficacy.^421^ Similar to the NHR mimetics, peptides engineered to emulate the CHR of gp41 were synthesized with the objective of impeding the formation of the six-helix bundle.^422^

Subsequently, building upon the foundation of peptides emulating CHR, an HIV-1 fusion inhibitor known as enfuvirtide was derived and granted approval for use in 2003 (Table 4, Figure 25).^423^ In addressing the rise and spread of enfuvirtide-resistant strains of HIV-1, a computational methodology contributed to the creation of sifuvirtide as an alternative fusion inhibitor. Sifuvirtide exhibited notable efficacy in impeding the formation of the six-helical bundle and demonstrated activity against HIV-1 variants resistant to enfuvirtide.^424^

**Figure 25:**
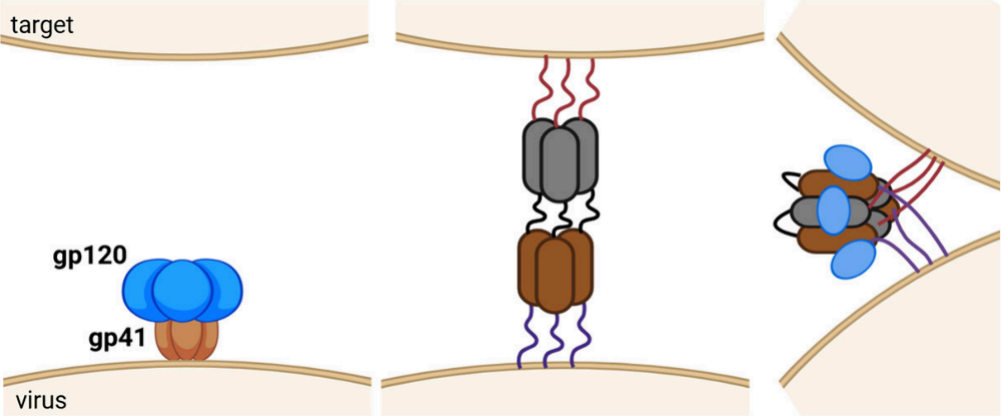
After the insertion of the fusion peptide, gp41 undergoes a conformational change, bringing its N-terminal and C-terminal regions together to form a stable six-helix bundle. This structural arrangement pulls the viral and cellular membranes into close proximity, facilitating lipid bilayer fusion. The formation of this six-helix bundle has inspired the development of peptide-based inhibitors. These inhibitory peptides mimic specific regions of gp41, preventing the assembly of the six-helix bundle and thereby blocking membrane fusion and viral entry. Created in BioRender.

**Table 4:** Antimicrobial Peptides Approved by FDA

Generic name	Brand name	Drug class	FDA first approval year	Company	Therapeutic indication	Route^a^
gramicidin	Gramicidin	ionophoric peptide	1952	J&J	minor skin infections and some eye infections	ophthalmic
vancomycin	Vancocin	cyclic glycopeptide	1958	Lilly	infections caused by Gram-positive bacteria	IV, O
bacitracin	Baciguent	cyclic	1962	Various manufacturers	prevent infection in minor cuts, scrapes, and burns caused by Gram-positive bacteria	T
polymyxin B	Bacitracin	cyclic lipopeptide	1964	Various manufacturers	severe bacterial bacterial infections caused by susceptible Gram-negative bacteria	IV, IM, T
daptomycin	Cubicin	cyclic lipopeptide	2003	Cubist	bacterial infections of the heart, skin, or blood caused by Gram-positive bacteria	IV
enfuvirtide	Fuzeon	viral entry inhibitor	2003	Roche	individuals with HIV-1 infection	SC
micafungin	Mycamine	cyclic lipopeptide	2005	Astellas	invasive candidiasis and prophylaxis for fungal infections	IV
dalbavancin	Dalvance	cyclic lipoglycopeptide	2014	Allergan	acute bacterial skin and skin structure infections caused by susceptible Gram-positive bacteria	IV
oritavancin	Orbactiv, Kimyrsa	cyclic lipoglycopeptide	2014	Melinta	acute bacterial skin and skin structure infections caused by susceptible Gram-positive bacteria	IV
polymyxin E or colistin	Coly-Mycin M	cyclic lipopeptide	2016	Various manufacturers	severe bacterial bacterial infections caused by susceptible Gram-negative bacteria	IV, IN
rezafungin	Rezzayo	antifungal	2023	Cidara Therapeutics	invasive fungal infections	IV

a IV: intravenous, IN: intranasal, SC: subcutaneous, IN: intramuscular, T: topical, O: orally.

Enfuvirtide, commercialized as Fuzeon or T20, was synthesized in industry via linear SPPS using the Fmoc strategy and HBTU/HOBt as coupling reagents, yielding approximately 8% of the final purified peptide. To improve efficiency, a hybrid SPPS/LPPS strategy was subsequently employed.^268^ In this approach, three Fmoc-protected fragments, each consisting of 9–16 amino acids, were synthesized on solid phase using 2-chlorotrityl chloride (2-CTC) resin. The peptide fragments were then cleaved from the resin under mild acidic conditions and assembled in solution. Specifically, the fragment Fmoc-AA27–35-OH was coupled to phenylalanine to generate Fmoc-H-AA27–36-NH_2_. Following Fmoc deprotection, the H-AA27–36-NH_2_ fragment was coupled to the Fmoc-AA17–26-OH fragment. After another Fmoc removal step, the resulting H-AA17–36-NH_2_ intermediate was coupled to Ac-AA1–16-OH to yield the fully protected enfuvirtide precursor (Ac-AA1–36-NH_2_) (Figure 26). The final global deprotection was carried out using TFA. The individual fragments were obtained with purities of approximately 90%, while the final deprotected peptide was isolated with a purity of about 75%. Overall, the synthetic process afforded a yield in the range of 85–90%.

**Figure 26:**
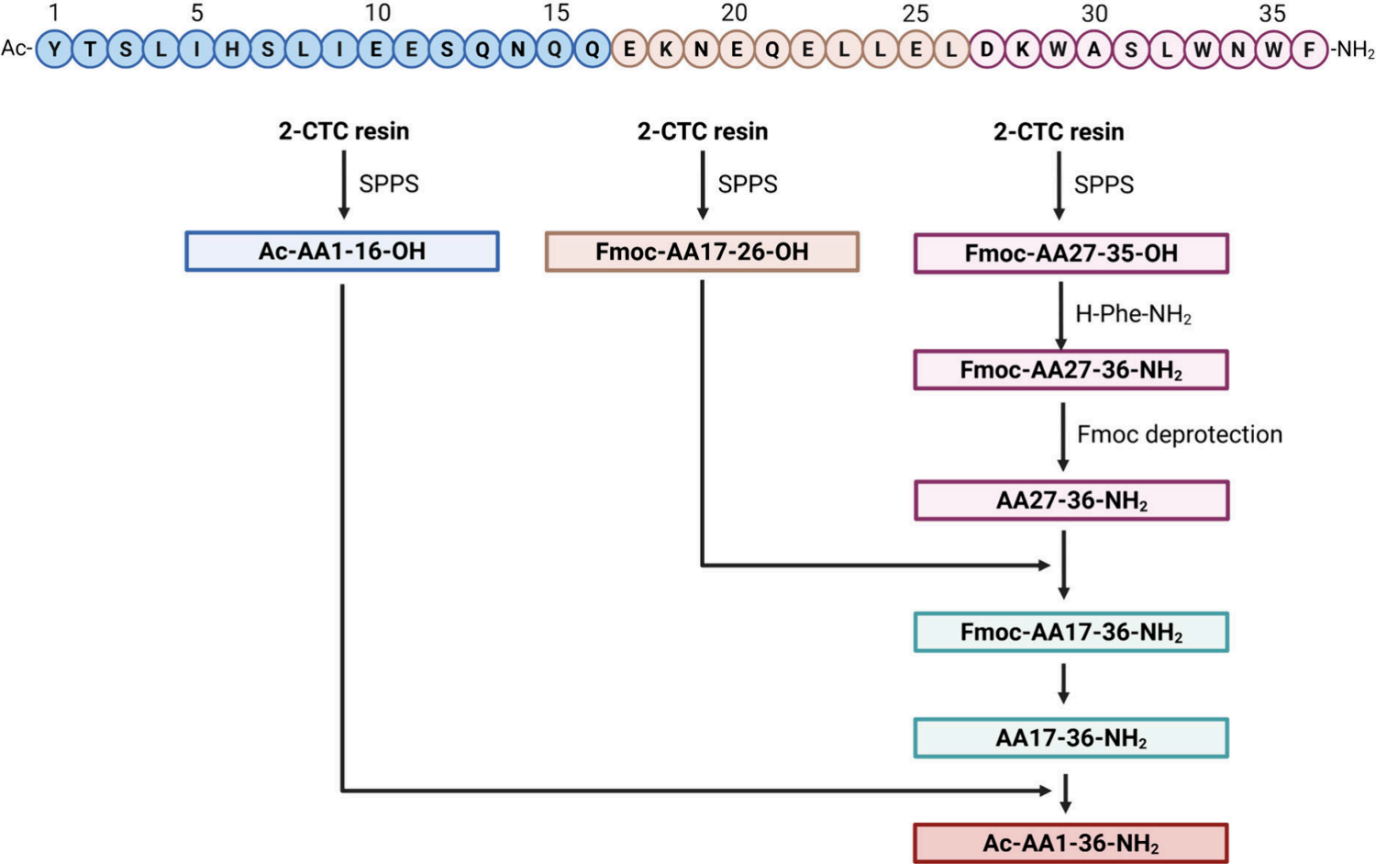
Enfuvirtide is a 36-amino acid peptide synthesized in three separate fragments that are subsequently assembled. As illustrated in the figure, the three fragments—represented in different colors—were synthesized on 2-chlorotrityl chloride (2-CTC) resin using HBTU as the coupling reagent and HOBt as a racemization suppressant. The fragments were designed to remain protected and soluble in DMF or NMP and to minimize epimerization during solution-phase condensation, with levels kept below 1%. Created in BioRender.

Sebsequently, Liskamp group has developed peptides that mimic the CD4-binding site, presenting three noncontiguous segments of the gp120 sequence. These peptides are arranged on a molecular framework as cyclic loops.^425^ This framework consists of triazacyclophane (TAC) scaffold. Specifically, the gp120-derived peptides were synthesized with cysteine residues at both the N- and C-termini and cyclized via a benzyl dibromide derivative. In this approach, the scaffold was also functionalized with azide groups, which enabled conjugation of the peptides to the TAC scaffold via CuAAC reaction (Scheme 17b).

**Scheme 17:**
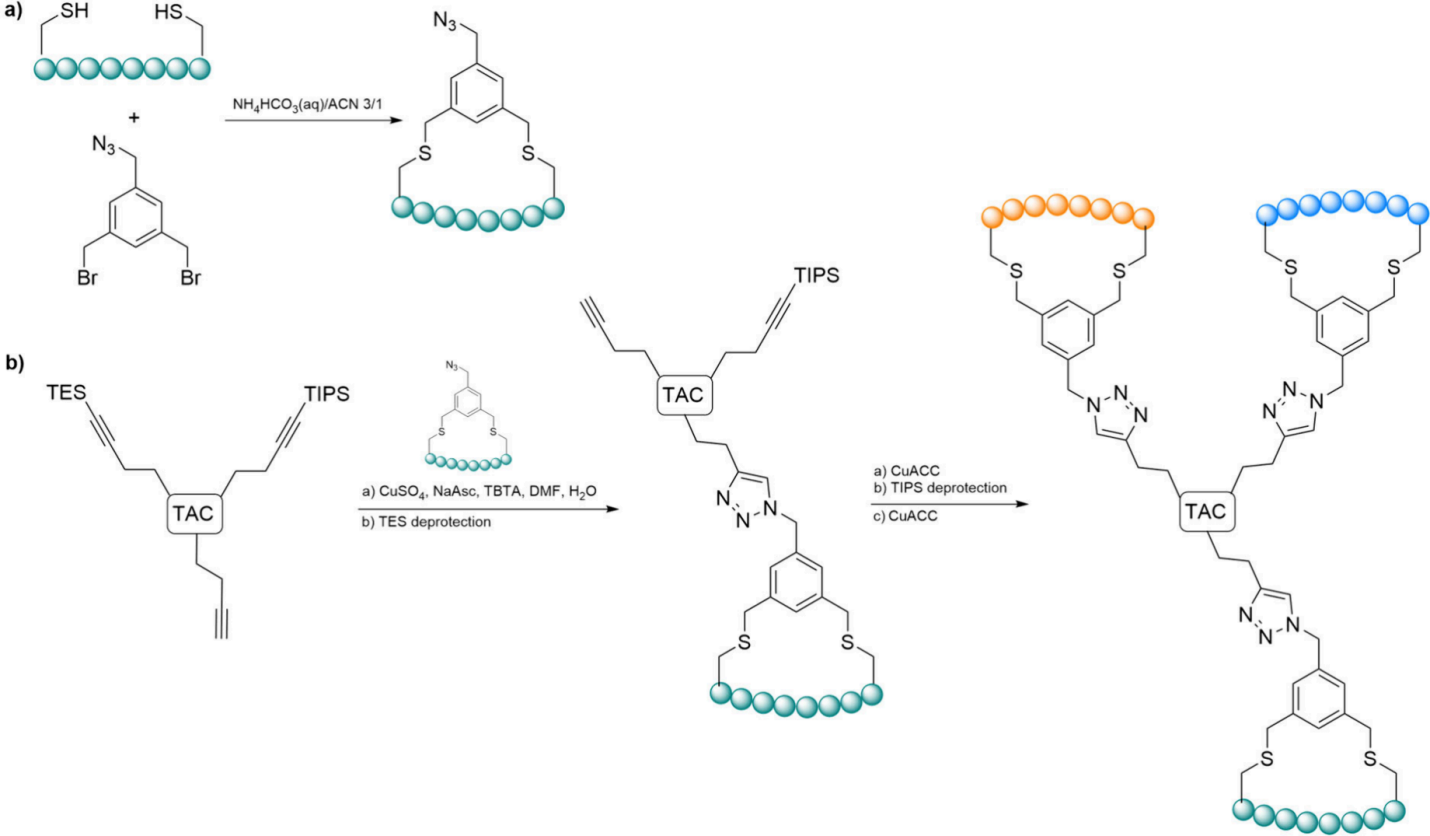
(a) General Synthetic Scheme for the Preparation of Azide-Bearing Cyclic Peptides. (b) Stepwise Conjugation of Azide-Functionalized Peptides onto an Orthogonally Protected Tri-alkyne Scaffold

However, this specific peptide configuration on the triazacyclophane scaffold did not exhibit a significant inhibitory effect on HIV-1 infection, indicating that alternative strategies are required to more effectively mimic the CD4-binding site. In addition, the Liskamp group demonstrated that the TAC scaffold could not prevent infection or neutralize HCV pseudoparticles. However, their data suggested the efficacy of discontinuous epitope mimics as potential synthetic vaccines.^426^

SARS-CoV-2 and SARS-CoV entry into cells relies on a crucial PPI between the spike glycoprotein (S-protein) receptor binding domain (RBD) of SARS-CoV and the protease domain (PD) of the human cell surface receptor angiotensin-converting enzyme 2 (ACE2). Earlier studies explored the use of medium-length linear peptides derived from ACE2, demonstrating micromolar affinity binding to the SARS-CoV-2 S-protein RBD, degradation of RBD, and inhibition of ACE2-receptor mediated host cell entry of SARS-CoV pseudovirus in vitro.^427^ However, linear peptides often exhibit suboptimal drug-like properties, including poor blood plasma stability, making them unsuitable lead compounds for drug discovery. In a recent investigation, efforts were made to create stable, conformationally constrained stapled analogues of the ACE2 PD helix α1 peptide. These analogues were designed to bind to the receptor-binding domain (RBD) of the SARS-CoV-2 S-protein, preventing interaction with native ACE2 receptors. The study suggests that larger ligands with enhanced binding interactions are necessary for effective binding to the SARS-CoV-2 S-protein RBD. This is crucial to outcompete membrane-bound ACE2 and efficiently inhibit viral infection.^428^

### 6.4 Addressing Antibiotic Resistance

Peptides have also been employed as therapeutic agents against infections, and several naturally derived peptides have received FDA approval. However, to date, only a limited number of peptide-based antibiotics have been approved. These include gramicidin (approved in 1952) and bacitracin (1962), along with glycopeptides like vancomycin (1958), and several cyclic lipopeptides, such as polymyxin B (1964), polymyxin E or colistin (2016), daptomycin (2003), dalbavancin (2014), and oritavancin (2014) (Table 4).^429–431^ Despite these successes, the development of new antibiotics remains a complex task, as both small molecules and peptides are susceptible to bacterial resistance mechanisms. The growing problem of antimicrobial resistance represents a major challenge in biomedical research, emphasizing the urgent need for novel antibacterial agents. For instance, resistance to vancomycin emerged around 1990, and to lipopeptides by 2005.^432,433^ Polymyxins, however, have retained their efficacy against highly resistant bacteria, although their use is limited by toxicity, especially to the kidneys, due to the lipid tail, and is therefore reserved for severe infections in hospital settings.^434^

Polymyxins share a similar structural framework but differ at position 6 by the presence of a D-amino acid—specifically, polymyxin B contains d-Phe, whereas polymyxin E (colistin) contains d-Leu. Several synthetic strategies have been developed to obtain polymyxins. Initial synthesis was carried out by Volger who reported a method involving the preparation of peptide fragments, which were assembled in solution and then cyclized using DCC.^435^ In contrast, Sharma later synthesized the peptide on a solid support and performed cyclization using diphenyl phosphoryl azide (DPPA) in the presence of DIEA.^436^ Other strategies involve the use of orthogonal protecting groups and their selective removal and replacement to enable controlled cyclization. For example, ivDde was used as a temporary protecting group, which was removed and replaced with Mmt; the latter was subsequently removed to allow for macrocyclization.^437^ In another approach, Cbz protection was employed for five amine functionalities prior to cyclization; however, the resulting peptide exhibited poor solubility in volatile solvents, complicating their removal by rotary evaporation after the cyclization step.^438^

Xu et al. reported a fully solid-phase synthetic approach for the preparation of colistin using a branched-chain strategy in which the side chain of diaminobutyric acid (Dab) was anchored to the resin. However, this method presents several limitations, including the time-consuming and costly preparation of the starting material Fmoc-Dab-OAllyl from Fmoc-Dab­(Boc)–OH, as well as the generation of a higher number of impurities.^439^ In contrast, Ramesh et al. demonstrated a more straightforward synthesis of a colistin analogue in which 6-methylheptanoic acid was substituted with decanoic acid.^440^ Their synthesis was performed on 2-CTC resin using the Fmoc/tBu strategy. An orthogonal protecting group approach was employed, where all Dab side chain amines were protected as Boc, threonine as tBu, and the amino group of Dab involved in the cyclization was protected as Alloc. This strategy proved advantageous, as the use of penta-Boc protection significantly improved the solubility of the peptide in common and volatile organic solvents such as DCM, compared to penta-Cbz-protected analogues. Details are reported in Scheme 18. Peptide cleavage from the resin was performed using a minimal amount of TFA/DCM (2%), and the filtrate was collected over a limited quantity of DMF. The inclusion of DMF facilitated the safe removal of TFA; in contrast, using only DCM could result in TFA accumulation during evaporation, potentially leading to premature deprotection of acid-labile side chain protecting groups. The presence of free amines at this stage would compromise the subsequent cyclization step, ultimately reducing the overall yield. Cyclization was conducted in solution, resulting in a high-yielding and convergent synthetic approach.

**Scheme 18:**
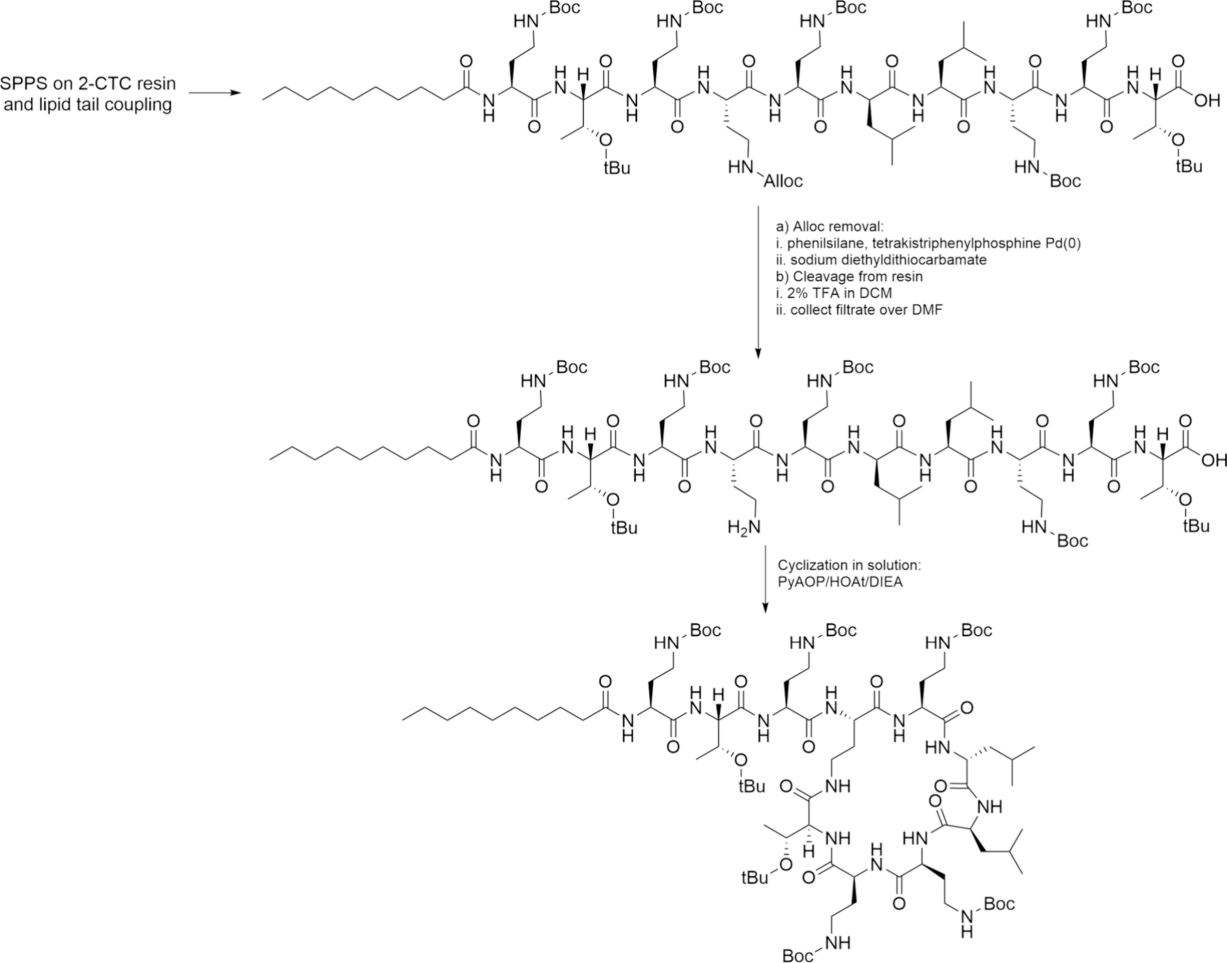
Synthesis Began with the Attachment of Fmoc-Thr­(tBu)–OH to 2-CTC Resin in the Presence of DIEA, Followed by Capping with Methanol^a^

a Subsequent amino acids were coupled using HBTU/DIEA in DMF, while the lipid tail was introduced using DIC/HOBt in DMF and allowed to react overnight. The choice of DIC was based on its demonstrated stability for prolonged coupling reactions in SPPS. The Alloc protecting group was removed while the peptide remained on the resin. Peptide cleavage was performed using 2% TFA in DCM, which enabled efficient release from the 2-CTC resin without removing the side-chain protecting groups. The partially protected linear peptide was then cyclized in solution using a DMF:DCM mixture in a 1:50 ratio.

Due to their associated toxicity, polymyxins are typically reserved as a last-resort treatment in hospital settings. Consequently, there is a growing demand for safer alternatives to combat antibiotic resistance. Both Gram-positive and Gram-negative bacteria are capable of developing resistance; however, Gram-negative pathogens are particularly difficult to treat due to their impermeable outer membrane and rapid acquisition of resistance mechanisms. For example, *Acinetobacter baumannii*, a major cause of hospital-acquired pneumonia and bloodstream infections, has developed resistance to multiple antibiotics, such as carbapenems, highlighting the urgent need for new therapies. In fact, for over 50 years, no new antibiotics specifically targeting *A. baumannii* have been successfully developed.

In an effort to address this gap, a new class of macrocyclic peptides (MCPs) has recently been introduced. Zampaloni et al. reported a series of tethered macrocyclic peptides whose mechanism of action involves blocking the transport of lipopolysaccharide (LPS) from the inner membrane to the outer membrane in Gram-negative bacteria by inhibiting the LptB_2_FGC complex.^441^ This class of peptides was identified through whole-cell phenotypic screening of 44,985 MCPs from Tranzyme Pharma against a panel of Gram-positive and Gram-negative human pathogens. A cluster of active compounds shared a common structural motif consisting of a tripeptide subunit and a diphenylsulfide tether that closed the macrocyclic ring. One compound, RO7036668—containing an Orn-Orn-N-Me-Trp subunit—demonstrated a minimum inhibitory concentration (MIC) of 4 mg/L against *A. baumannii* ATCC 19606. Subsequent optimization, including the substitution of the central l-Orn with l-Lys, dichloro modifications on the benzene ring, and the replacement of the southwestern phenyl ring with pyridine, led to the identification of RO7075573. This analog exhibited up to a 64-fold increase in potency over the initial lead. Further development yielded zosurabalpin, a compound with improved pharmacokinetic properties and in vivo efficacy. In mouse models of MDR and carbapenem-resistant *A. baumannii* infections, subcutaneous administration of RO7075573 provided complete protection from lethal sepsis and significantly reduced bacterial burden in a thigh infection model. However, intravenous administration in rats led to toxicity, likely due to lipid precipitation in plasma. To overcome this limitation, the compound was modified with a zwitterionic tether, resulting in zosurabalpin. This analog retained potent antibacterial activity while demonstrating improved plasma stability and tolerability. Zosurabalpin exhibited favorable physicochemical properties for clinical development and showed strong in vitro activity against various MDR *A. baumannii* strains. In mouse models of pneumonia, thigh infection, and sepsis, zosurabalpin effectively reduced bacterial loads and improved survival outcomes.

While antibiotic R&D has seen a slowdown, two recent industry-academic collaborations have identified novel antimicrobial peptide classes targeting Gram-positive bacteria. Ten years after the discovery of a complex of eight related acyldepsipeptides (ADEPs) active against *Staphylococcus* and *Streptococcus* species, Labischinski and colleagues reported in *Nature Medicine* the structure of the main peptide component, ADEP1, and described optimized synthetic variants with enhanced antibiotic properties.^442^ Two of these optimized peptides, ADEP2 and ADEP4, exhibited superior in vitro potency against Gram-positive bacteria compared to ADEP1. In rodent models with lethal *Enterococcus faecalis* infections, ADEP2 and ADEP4 matched the effectiveness of linezolid, a clinically used antibiotic. ADEP4 achieved an 80% cure rate in a sepsis model and outperformed linezolid against *Streptococcus pneumoniae* infections in rodents. The researchers identified the bacterial caseinolytic protease (ClpP) as the ADEP target, demonstrating that ADEPs bind to ClpP, activate the otherwise inactive Clp-protease complex, and disrupt essential bacterial protein regulation, potentially accounting for their potent antibacterial effects.

In this context, given the urgent need to combat antibiotic resistance and the promising properties of peptides, numerous academic research groups are actively investigating antimicrobial peptides as potential therapeutic agents. Antimicrobial peptides (AMPs) offer a promising alternative foundation to fight bacteria, especially Gram-negative bacteria.^443,444^ AMPs are short, positively charged, amphipathic molecules that are evolutionarily conserved across diverse organisms and function as natural immune effectors against pathogens. AMPs are among the oldest evolutionary defenses against microbial threats found across the plant and animal kingdoms. Their structural diversity, shaped by the unique environments of different species, provides effective, rapid, and adaptive responses to pathogens. This diversity has allowed AMPs to avoid becoming obsolete in the face of bacterial evolution, positioning them as critical agents in developing new therapeutics. Several factors make resistance development against AMPs particularly challenging. First, AMPs typically disrupt bacterial cell membranes through nonspecific binding, leading to cell lysis—a mode of action that hinders resistance (Figure 27).^445,446^ Additionally, AMPs interfere with bacterial cell wall and protein synthesis, providing a dual-action mechanism that further complicates bacterial adaptation.

**Figure 27:**
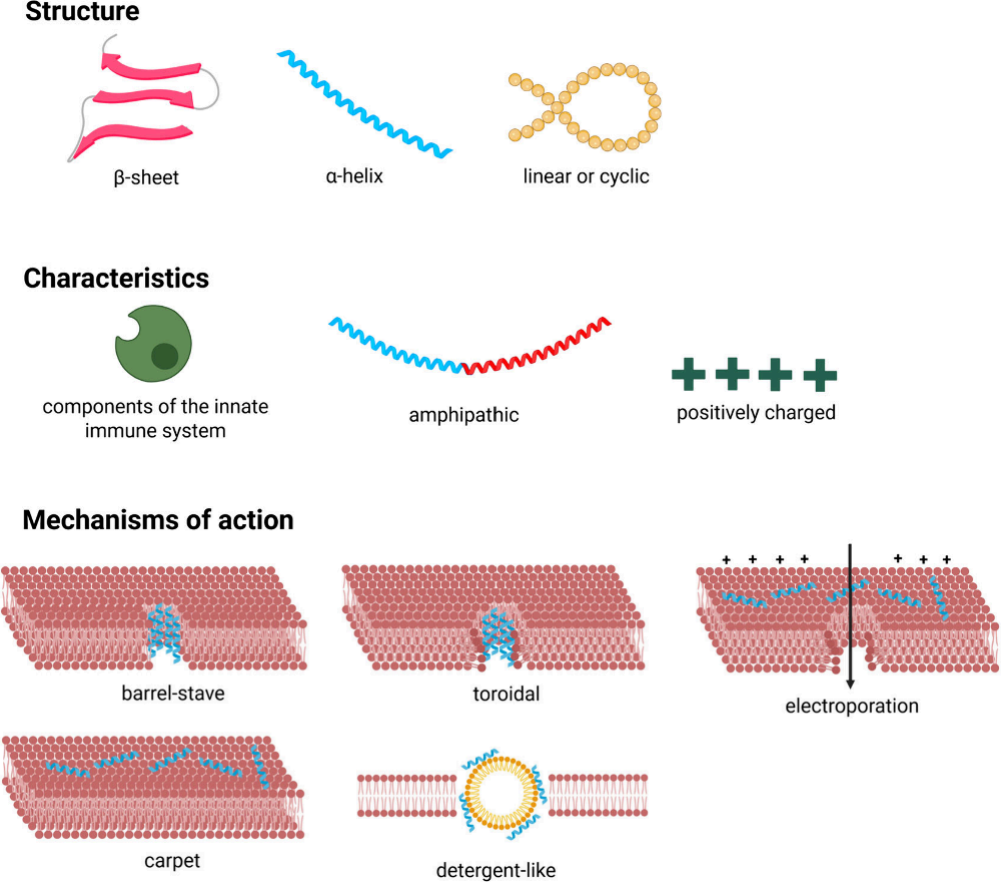
Antimicrobial peptides (AMPs) are abundant in nature and can be found in animals, plants, and microorganisms. They exhibit a variety of structural forms, including helical peptides (e.g., melittin), β-sheet peptides (e.g., defensins), and cyclic peptides (e.g., polymyxins). These peptides are typically rich in positively charged residues and adopt amphipathic conformations upon folding. Due to their unique physicochemical properties, AMPs interact directly with bacterial lipid membranes, leading to membrane disruption. Their mechanisms of action include barrel-stave model (peptides insert into the membrane, forming transmembrane pores), toroidal pore formation (peptides induce curvature in the lipid bilayer, creating a pore lined by both peptides and lipid head groups), micelle-like aggregation (peptides behave like detergents, disrupting the membrane and forming micelle-like structures), carpet model (peptides cover the membrane surface like a carpet, disrupting the bilayer through a collective destabilization), and electrostatic potential alteration (the presence of positively charged residues can alter the membrane potential, potentially causing pore formation or membrane depolarization. These mechanisms disrupt bacterial integrity, leading to cell death, making AMPs potent agents against a broad spectrum of pathogens. Created in BioRender.

AMPs exhibit broad-spectrum efficacy, targeting a wide range of pathogens, including Gram-positive and Gram-negative bacteria, fungi, and some viruses. Their rapid action—often within minutes—can overwhelm bacterial defenses before resistance mechanisms can be upregulated. Attempts by bacteria to alter cell membranes to evade AMP binding would likely impair their viability, underscoring the potential of AMPs as effective therapeutic agents. Moreover, AMPs synergise well with other antimicrobials, enhancing overall effectiveness and further reducing the likelihood of resistance. AMPs also play a role in shaping the microbiome, fostering beneficial bacterial populations and discouraging pathogenic overgrowth, which helps sustain a balanced microbial environment with fewer opportunities for resistance. Given these advantages, AMPs represent a compelling starting point for the development of next-generation antibiotics capable of addressing both existing and emerging antibiotic-resistant infections.^447^

Defensins and cathelicidins (e.g., LL-37), two mammalian AMPs, are particularly interesting templates for drug design due to their effectiveness against microbial cell membranes. Defensins, classified into α-, β-, and θ-defensins, are distinguished by unique disulfide bridge arrangements.^448^ They can be found in vertebrate and invertebrate animals, plants and fungi. Cathelicidins, found in various vertebrates, are primarily produced in epithelial cells, neutrophils, and macrophages, with LL-37 being the only human member of this group.^449^ Meanwhile, other AMPs have been identified in insects, like cecropins and melittin, and bacteria, such as nisin and lysostaphin, though none have received FDA approval to date due to the need for further studies to address toxicity concerns.^450–453^

Another subset of cationic antimicrobial peptides featuring β-hairpin structures stabilized by disulfide bridges includes protegrins, polyphemusins, and tachyplesin. Employing a β-hairpin mimetic strategy, Robinson and colleagues demonstrated heightened antimicrobial efficacy and prolonged plasma half-life using peptide loops similar to protegrin-1 were attached to the d-Pro-l-Pro template.^454^ The disulfide bridges were substituted with various residues, leading to the discovery of a family of template-bound protegrin mimetics. Screening these mimetics identified analogues with potent broad-spectrum antimicrobial activity and significantly reduced hemolytic effects. They exhibited direct interaction with the bacterial β-barrel protein LptD in *Pseudomonas spp*. This interaction involves the lipopolysaccharide transport during outer membrane biogenesis, distinguishing them from other antimicrobial peptides primarily acting through membranolytic activity. The optimal quantitative retention-activity relationship (QRAR) model suggested that antimicrobial potency correlates with peptide charge and amphipathicity, while hemolytic effects correlate with the lipophilicity of residues forming the nonpolar face of the β-hairpin.

Furthermore, AMPs can prevent biofilm formation and dissolve established biofilms, a common cause of persistent infections.^455^ Their versatility and ease of modification enable the design of innovative biomaterials, such as peptide-based hydrogels with antimicrobial properties that could effectively target bacterial resistance in both acute and chronic infections. For example, the antimicrobial peptide WMR, selected for its strong antibacterial activity, is tested for enhanced antibiofilm effects against *Pseudomonas aeruginosa*, a Gram-negative bacterium, and *Candida albicans*, a pathogenic fungus. The study demonstrates how the multivalent modifications of WMR peptide with short, charged sequences (GDDS and WKRS) on self-assembled nanostructures significantly boost antibiofilm properties, providing an effective approach to tackling biofilm-associated infections (Figure 28).^456^ This nanosystem offers a promising strategy for designing responsive materials with heightened antibacterial effectiveness and potential for controlled drug release.

**Figure 28:**
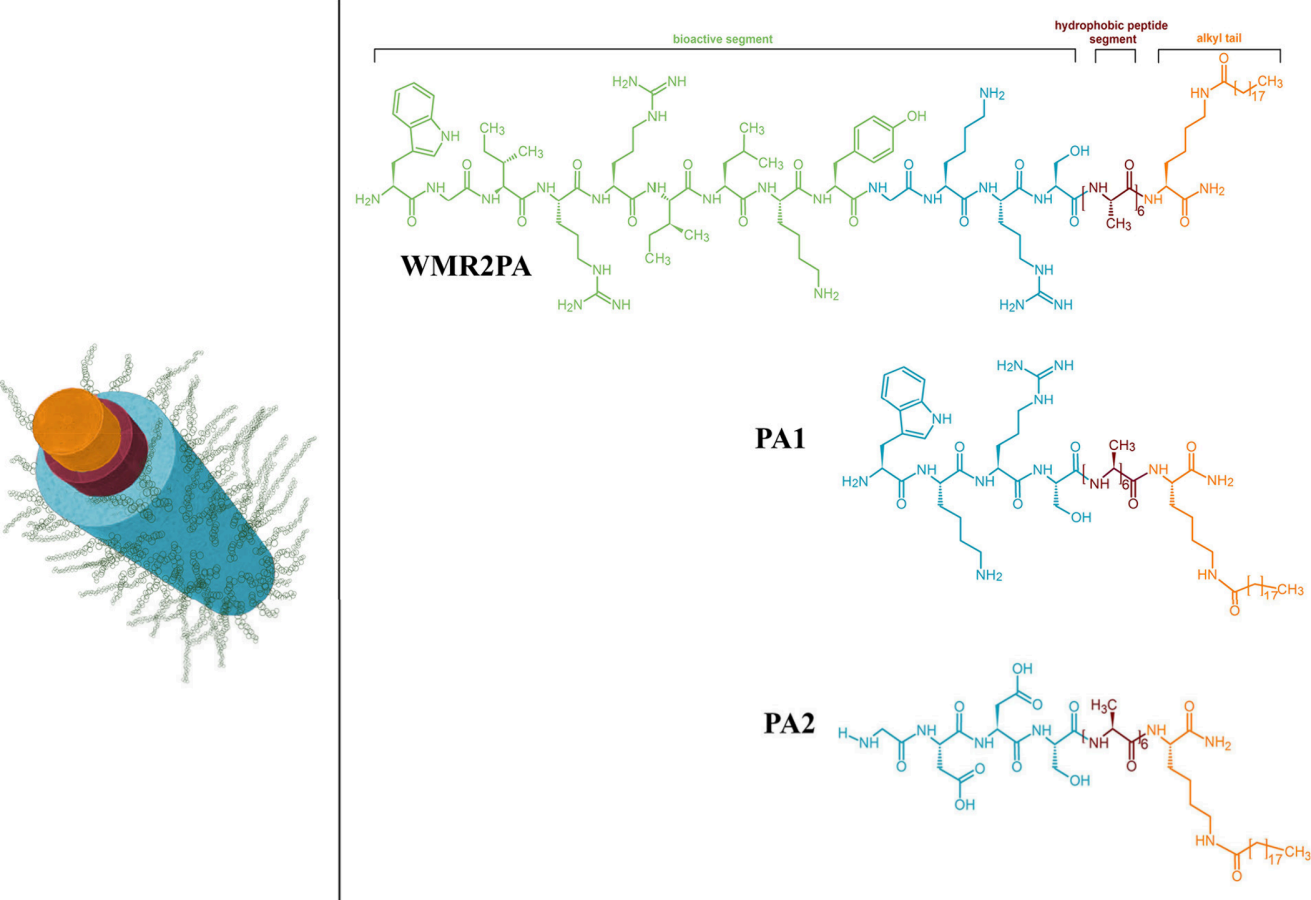
Molecular structures of WMR2PA, PA1, and PA2 (right) and their proposed self-assembled nanostructures (left) are shown. In these assemblies, the bioactive segment is exposed on the surface, while the hydrophobic alkyl tail is buried in the core, providing the driving force for self-assembly. Reproduced with permission from ref 456. Copyright 2019, American Chemical Society.

### 6.5 Development of Anti-inflammatory Compounds and Painkillers

Peptidomimetics targeting voltage-gated sodium channels (VGSCs) have drawn significant attention as promising analgesics. Research on pain-targeting peptides began in the 1980s, spurred by the discovery of conotoxins in the venom of cone snails.^457^ These peptides have shown potential as selective chemical tools for targeting ion channels and receptors. Conotoxins are typically composed of 10–40 amino acids, rich in disulfide bonds, which give them the structural stability to selectively and powerfully interact with ion channels, GPCRs, and transporters. To date, five distinct classes of conotoxins have been identified (α-, δ-, κ-, μ-, ω-type), each characterized by a unique molecular target. Among these, ω-conotoxins specifically inhibit Ca_v_2.2 channels, also referred to as N-type voltage-gated calcium channels. These channels are predominantly expressed at nerve terminals, dendrites, and in neuroendocrine cells, where they play a pivotal role in neurotransmitter release and are involved in pain transmission.^458^

Ziconotide, a synthetic analogue of the conotoxin peptide ω-MVIIA, is an FDA-approved drug for severe and chronic pain management. This conotoxin contains 25 amino acids and three disulfide bridges, stabilizing a small β-sheet that selectively inhibits the Ca_v_2.2 channel.^459^ Approved in 2004, it gained attention for being 1,000 times more potent than morphine without the risk of addiction. However, its drawback lies in its delivery method, requiring infusion via a pump directly into the cerebrospinal fluid to reach Ca_v_2.2 channels located in spinal cord neurons.^460^ Nevertheless, the challenging physicochemical properties of native peptides have led to active research efforts in developing conotoxin peptidomimetics within both academia and the pharmaceutical industry.

Ziconotide is a 25-residue peptide containing six cysteine residues that form three disulfide bridges, which are essential for its structural integrity and bioactivity. Key residues involved in the selective interaction with N-type voltage-gated calcium channels include lysine at position 2, arginines at positions 10 and 21, leucine at position 11, and the N-terminal amine.^459^ A major challenge in the industrial-scale synthesis of ziconotide lies in (a) achieving a high-yield synthesis of the linear 25-mer precursor, and (b) promoting efficient, native disulfide bond formation. Due to the presence of multiple cysteines and the need for precise disulfide bridge formation, orthogonal protection strategies are typically required. However, these methods are costly and often unsuitable for large-scale production.

Attempts to promote disulfide bond formation using redox buffers such as oxidized and reduced glutathione (GSSG/GSH) to mimic the cellular folding environment have resulted in significant formation of scrambled and misfolded isomers that are difficult to separate via HPLC.^461^ As such, despite their cost, orthogonal synthesis approaches remain preferable for obtaining the correctly folded product. Zhang et al. recently reported an efficient method for synthesizing conotoxins with three disulfide bonds using Mob, Trt, and Acm protecting groups to enable regioselective disulfide formation. Their strategy allowed for the successful synthesis of five conotoxins with correct disulfide connectivities, yielding 20–30%.^462^

In efforts to develop an orally bioavailable ziconotide analogue, cyclization has been explored as a strategy to enhance peptide stability. However, synthesizing a cyclic form of ω-conotoxin MVIIA has proven challenging. Backbone-cyclized analogues with fewer disulfide bonds have been reported, but these often lack structural integrity and are likely to show compromised activity.^463^ Cyclization via native chemical ligation using a GGPG linker has also been attempted, but the oxidized product was neither structurally characterized nor functionally evaluated.^464^

A study came from the Craik group, which employed an asparaginyl endopeptidase (AEP)-mediated cyclization strategy to generate backbone-cyclized analogues of MVIIA.^465^ Several AEP isoforms derived from plants demonstrated the endopeptidase and transpeptidase activity necessary for the head-to-tail cyclization reaction, a key step in the biosynthesis of cyclotides.^466^ Linear ziconotide analogues incorporating a linker sequence were synthesized via Fmoc-based SPPS, followed by cleavage, deprotection, and purification. Disulfide bond formation was carried out in NH_4_OAc/GnHCl buffer at pH 6.5, yielding a dominant correctly folded isomer at about 25% purity (Figure 29). Postfolding, the peptides were incubated with AEP to generate their cyclic counterparts. Cyclic MVIIA analogues, incorporating six- to nine-residue linkers composed primarily of glycine and alanine for minimal steric hindrance, retained structural fidelity. Methionine at position 12 was substituted with norleucine to avoid oxidation-related instability. These cyclic analogues inhibited voltage-gated calcium channels and exhibited significantly enhanced stability in human serum and simulated intestinal fluid. This study highlights the potential of AEP-mediated enzymatic cyclization as a powerful tool for generating structurally complex, cyclic peptide therapeutics—offering a viable route to improving the pharmacological properties and therapeutic value of conotoxins beyond the reach of conventional chemical synthesis.

**Figure 29:**
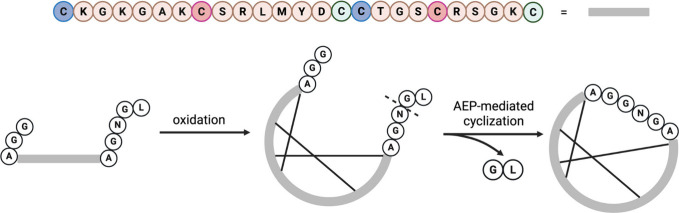
Strategy for the synthesis of cyclic MVIIA analogues. Top: Sequence of ziconotide, with identical colors indicating cysteine residues involved in the same disulfide bond. Bottom: AEP-mediated cyclization following oxidative folding. Created in BioRender.

The group led by Jamieson has pioneered a novel series of conformationally constrained peptidomimetic analogues inspired by the μ-conotoxin KIIIA, extracted from the venom of the marine cone snail Conus kinoshitai.^467^ They evaluated the activity of these mimetics against human VGSCs and identified two compounds that effectively blocked currents in hNav1.4 and hNav1.6 channels. The primary objective of their investigation was to explore whether synthetic conformational constraints could replace the intricate disulfide bond bridging network in the μ-KIIIA conotoxin peptide, resulting in more stable analogues that retained bioactivity against human VGSCs. The group devised simplified structures based on μ-KIIIA by substituting the complex disulfide-bonding network with chemical staple conformational constraints. They synthesized seven i, i+4, and i, i+7 stapled mimetics using various chemistries, including hydrocarbon, triazole and lactam stapling, and compared them to native μ-KIIIA isomers and three nonstapled control compounds. Notably, only compounds featuring the i, i+7 staples demonstrated low micromolar inhibition of the tested human sodium channels, Nav1.4 from skeletal muscle and NaV1.6 from the CNS.

Other analgesic peptides can be engineered by targeting components of the innate immune system, such as complement factor C5a—a potent pro-inflammatory mediator that recruits leukocytes and activates phagocytic responses. CHIPS (Chemotaxis Inhibitory Protein of *Staphylococcus aureus*) is well-known for its ability to antagonize the C5a receptor (C5aR), thereby blocking the interaction between C5a and its receptor—a critical axis in complement-mediated immune activation. Building on the structural framework of CHIPS, a novel anti-inflammatory peptide named CHOPS has been synthetically engineered. Given the high immunogenicity associated with the full-length CHIPS protein, CHOPS was designed as a minimized analogue that retains the essential receptor-binding residues identified from structural studies of CHIPS in complex with C5aR.^468^ CHOPS was constructed by linking two critical CHIPS-derived fragments—residues T36–L65 (N-terminal) and K95–G112 (C-terminal), both of which contribute to C5aR binding. These segments were joined using a d-Pro-Gly dipeptide linker, which promotes the formation of helical and β-sheet elements characteristic of the native CHIPS structure (Figure 30). The resulting peptide is specifically tailored to engage C5aR while minimizing the risk of immune activation. This rationally designed analogue presents a promising therapeutic strategy for the treatment of inflammatory and autoimmune disorders.

**Figure 30:**
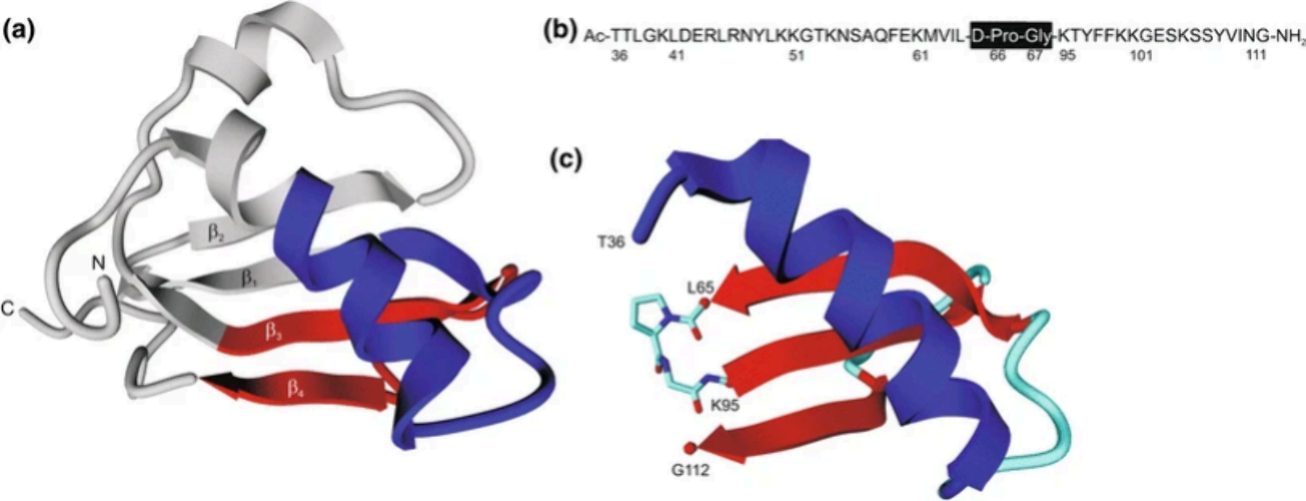
a) Cartoon representation of the NMR structures of CHIPS31–121 (PDB ID: 1XEE). The two regions interacting with the C5a receptor (C5aR) are the segments 43–61 (α-helix and β1 strand) and 95–111 (β3 and β4 strands). (b) Amino acid sequence of CHOPS. The d-Pro-Gly linker is indicated in black. c) Cartoon representation of CHOPS modeled based on the structure of CHIPS31–121. The d-Pro-Gly linker is depicted in stick representation. Reproduced with permission from ref 468. Copyright 2010, Springer Nature under the Creative Commons Attribution Noncommercial License (https://creativecommons.org/licenses/by-nc/2.0).

Due to its inherent conformational flexibility, the leu-enkephalin peptide demonstrates the capability to bind to various opioid receptors. While effective in pain relief, this peptide carries potential side effects such as miosis and the risk of physical dependency. Unfortunately, leu-enkephalin encounters challenges related to poor bioavailability and susceptibility to proteolytic degradation, limiting its suitability as a therapeutic agent. To address these issues, researchers have explored macrocyclic mimetics as an alternative approach. Blomberg and collaborators have detailed the incorporation of a β-turn mimetic, encompassing both 10- and 7-membered rings, to replace the initial four residues of leu-enkephalin.^469^ The 7-membered ring analogue lacks one glycine in the sequence, while the 10-atom cycle adopts a β-turn conformation. In both mimetics, the intramolecular hydrogen bond has been replaced with an ethylene bridge, and the amide bond between Tyr1 and Gly2 has been substituted with an isostere composed of methylene ether (Figure 31). Characterization of these analogues was compared to their respective linear counterparts. This study has unveiled that all analogues, with the exception of the β-turn mimetic, can effectively interact with opioid receptors, providing insights into the mechanism of action of this peptide.

**Figure 31:**
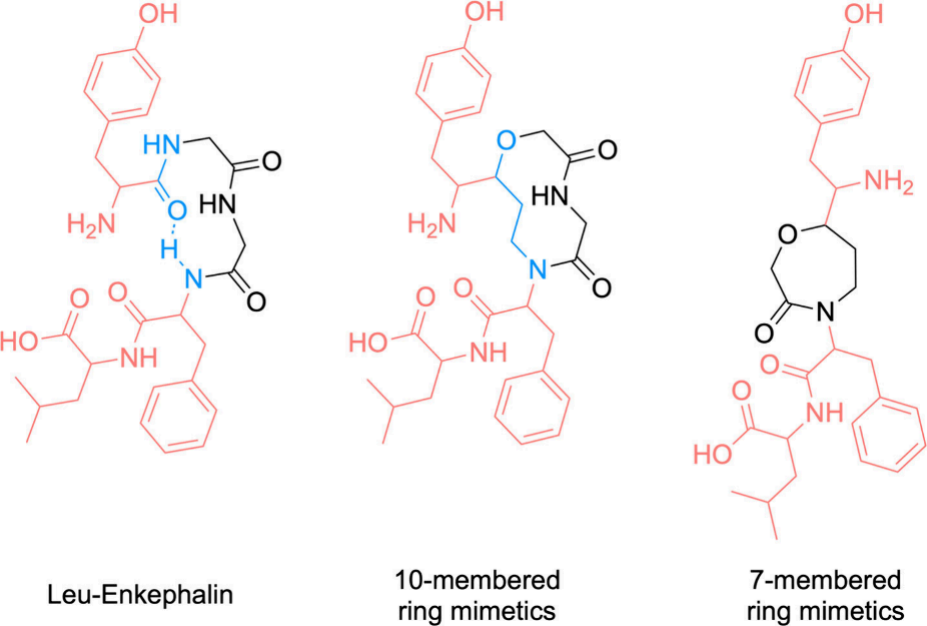
A peptidomimetic (center) designed with a covalently bonded 10-membered ring that mimics the β-turn observed in crystalline leu-enkephalin (left). A second peptidomimetic (right) features a 7-membered ring, inducing a different turn conformation compared to leu-enkephalin. Atoms highlighted in red are conserved across all structures, while atoms in blue represent the original segment from leu-enkephalin and its corresponding portion in the second peptidomimetic.

Other FDA-approved peptides that act on the nervous system include difelikefalin and trofinetide.^470,471^ Unlike conotoxins, these peptides have simpler, shorter, and linear structures, lacking cyclic components. They are used for the treatment of itch and Rett syndrome, respectively (Table 5).

**Table 5:** Peptide-Based Painkillers Approved by FDA

Generic name	Brand name	Drug class	FDA first approval year	Company	Therapeutic indication	Route^a^
ziconotide	Prialt	calcium channel blocker	2004	Elan Corporation	severe chronic pain in patients for whom intrathecal therapy is warranted	IT
difelikefalin	Korsuva	k opioid receptor agonist	2021	Cara Therapeutics	pruritus (itching) associated with chronic kidney disease	IV
trofinetide	Daybue	glycine-proline-glutamate (GPE) analog	2023	Acadia Pharmaceuticals	Rett syndrome in pediatric patients	O

a IV: intravenous, IN: intramuscular, O: orally.

## 7 Oral Administration of Peptide Drugs

Oral administration remains the most patient-friendly route of drug delivery. However, achieving effective bioavailability for large, hydrophilic peptides remains a formidable challenge. Along their journey from ingestion to absorption, peptides encounter multiple physiological barriers that significantly hinder their therapeutic potential (Figure 32). One of the primary obstacles is the harsh gastric environment. Peptides are generally unstable in the acidic pH of the stomach and are susceptible to enzymatic degradation by pepsins, rendering them unsuitable for oral delivery. Further down the gastrointestinal tract, peptides face proteolysis by intestinal enzymes, including endopeptidases such as trypsin, chymotrypsin, and elastase, as well as exopeptidases like aminopeptidase N, dipeptidases, and carboxypeptidases A and B. In addition, lysosomal enzymes within intestinal epithelial cells (enterocytes) further contribute to peptide degradation.^472^

**Figure 32:**
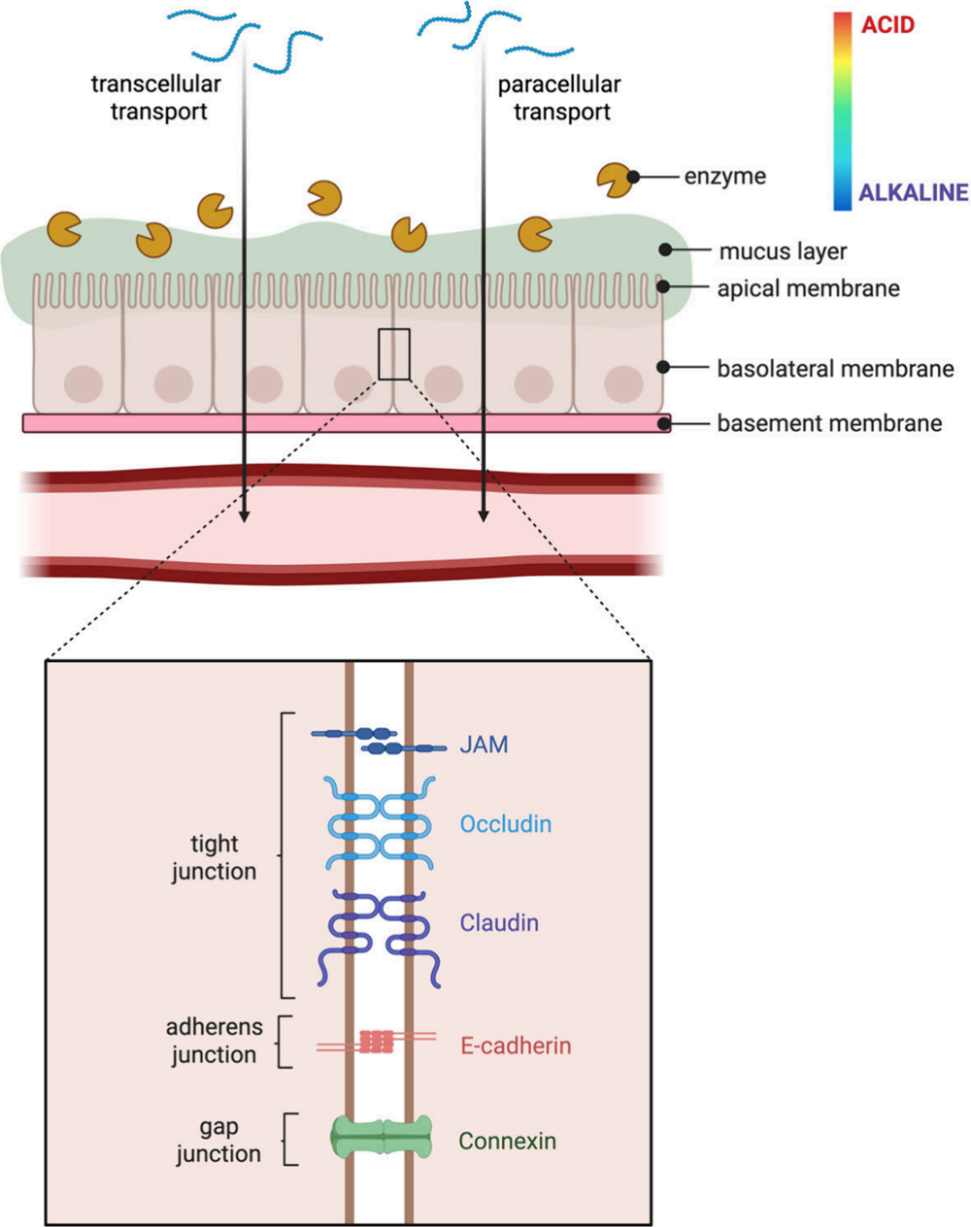
Illustration of the main barriers faced by peptides during oral absorption. After oral administration, peptides first encounter the acidic environment of the stomach, where the pH is low, leading to potential degradation. As they progress toward the intestinal epithelium, the pH becomes more basic. Peptides must then penetrate the mucus layer that protects the underlying intestinal epithelial cells (enterocytes) and avoid degradation by various digestive enzymes. Upon reaching the enterocytes, peptides can cross the epithelial barrier either via transcellular transport (passing through the cells) or via paracellular transport (passing between the cells through transient openings in the tight junctions). Created in BioRender.

Beyond enzymatic breakdown, the intestinal mucus layer poses another significant barrier. While its mesh-like structure features pores large enough to permit peptide diffusion, interactions between peptides and mucus components—particularly hydrophobic interactions and hydrogen bonding—can severely impede their diffusion.^473^ Furthermore, peptides containing thiol or disulfide groups are especially vulnerable to thiol–disulfide exchange reactions, which can inactivate the drug. These exchanges may occur with glutathione, cysteine-rich mucus glycoproteins, or dietary proteins containing free cysteine residues.^474^ Following these barriers, peptides must still traverse the intestinal epithelium to reach systemic circulation. This can occur via two primary routes: the transcellular pathway, which involves crossing the apical membrane, migrating through the cytoplasm, and exiting via the basolateral membrane; or the paracellular route, which requires passage through tight junctions between adjacent epithelial cells. The latter is highly restrictive, permitting only small and transient openings that limit paracellular transport of peptide drugs.^475^

Various approaches can enhance peptide transport and intestinal absorption. Modifying peptide sequences to make them less susceptible to protease degradation is effective; common strategies include using nonproteinogenic amino acids or cyclizing the peptide (Section 3). However, these modifications are often insufficient and require additional protective systems. Lipid- or polymer-based carriers are among the most effective systems, serving as protective shields and transport vehicles for peptides.^476,477^ Additionally, an enteric coating can help improve peptide absorption through the gastrointestinal (GI) tract. Enteric coating is a polymeric barrier, such as methacrylic acid copolymers and hydroxypropyl methylcellulose phthalate, applied onto the surface of the oral drug with the aim of protecting it from the acidity of the stomach and release the drug in the upper tract of the intestine.^478,479^

One of the most widely studied strategies is the use of permeation enhancers in peptide administration. These enhancers are typically nonionic surfactants chosen for their low toxicity and minimal reactivity. When included in drug formulations, surfactants can enhance peptide permeation by integrating into the cell membrane, disrupting the structural integrity of the lipid bilayer. This disruption compromises the membrane’s barrier function, increasing permeability and fluidity.^480^ Several surfactants, including sodium dodecyl sulfate, sodium taurodihydrofusidate, polyoxyethylene ethers, and medium- to long-chain fatty acids, such as capric acid (decanoic acid) and caprylic acid (octanoic acid), have been used in oral drug formulations, often in combination with other carriers.

Despite their therapeutic potential, there are currently few examples of peptides successfully administered via the oral route; the majority are still delivered through injections. However, extensive research is ongoing to overcome the challenges associated with oral peptide delivery, and several candidates are currently undergoing clinical evaluation. For instance, novel oral formulations of peptides such as insulin (ORMD-0801), calcitonin (SMC021), and difelikefalin—administered in combination with permeation enhancers—have progressed to clinical trials, with some reaching Phase 3.^481–483^ Additionally, oral formulations of leuprolide have advanced to Phase 2 trials.^484^

Novo Nordisk developed an oral formulation of semaglutide, marketed as Rybelsus, which is administered once daily. This formulation utilizes the Eligen technology created by Emisphere Technologies, where semaglutide is coformulated with sodium N-[8-(2-hydroxybenzoyl) amino] caprylate (SNAC). SNAC acts as an absorption enhancer, facilitating gastrointestinal uptake (Figure 32).^485^ SNAC functions by forming a noncovalent complex with semaglutide, thereby increasing its lipophilicity and enabling transcellular transport across the gastrointestinal epithelium. The SNAC-based formulation used for Rybelsus also includes additional absorption enhancers such as N-(5-chlorosalicyloyl)-8-aminocaprylic acid (5-CNAC), 4-([4-chloro-2-hydroxybenzoyl]-amino) butanoic acid (4-CNAB), and N-(10-[2-hydroxybenzoyl]-amino) decanoic acid (SNAD). These components form a complex with the peptide that remains insoluble at low pH, thereby protecting it from degradation by gastric peptidases. Upon reaching the small intestine, where the pH exceeds 7, the complex dissociates, allowing the peptide to be absorbed efficiently.^486^

Moreover, the absorption specificity depends on the properties of the therapeutics used. For instance, liraglutide is not absorbed when coformulated with SNAC, likely due to its higher hydrophobicity. Similarly, using a closely related analogue of SNAC does not facilitate the absorption of semaglutide, underscoring the importance of the specific interaction between semaglutide and SNAC for effective oral.^486^ These findings underscore the challenges of translating absorption-enhancing technologies from one drug candidate to another, even within the same therapeutic class.

AstraZeneca has also developed an alternative formulation for oral administration of a peptide-based antdiabetic therapeutic, which has been directly compared to oral semaglutide in terms of pharmacokinetics, bioavailability, and clinical efficacy. They modified the native GLP-1 peptide by incorporating multiple α-methyl amino acids at specific positions vulnerable to proteolytic attack, resulting in the analogue J211. To extend its circulating half-life, lipidation was performed at position 26, similar to the modification strategy used for semaglutide, where lysine at position 26 was conjugated with a linker and a C_18_ dicarboxylic lipid. For enhanced potency, J211 underwent a lipidation scan to identify optimal sites for lipid attachment.^487^ As a result, positions 19 and 31 were substituted with lysine residues, which were further functionalized with dodecanoic acid, producing MEDI7219, the first bis-lipidated GLP-1 analogue. MEDI7219 was formulated as enteric-coated oral tablets containing 100 mg of sodium chenodeoxycholate (NaCDC) and 200 mg of propyl gallate (PG) as permeation enhancers. The enteric coating was designed to protect the formulation from the acidic environment of the stomach and ensure drug release in the neutral pH of the intestine. For comparison, semaglutide tablets were formulated without enteric coating, incorporating 20 mg of the peptide and 300 mg of SNAC as the permeation enhancer. In pharmacokinetic studies, the oral bioavailability of MEDI7219 in dogs was significantly higher than that of semaglutide (5.92% vs 0.08%). However, MEDI7219 exhibited a shorter plasma half-life compared to semaglutide (9.8 h vs 60.5 h), consistent with the lower plasma protein binding observed in vitro. These pharmacokinetic parameters suggest that MEDI7219 is suitable for once-daily oral dosing in its current tablet formulation.

Several studies have investigated the use of protease inhibitors to improve the bioavailability of orally administered proteins. For example, the coadministration of calcitonin with aprotinin reduced calcitonin degradation in the colon but did not increase its plasma concentration.^488^ Small-molecule inhibitors such as camostat mesylate, bacitracin, soybean trypsin inhibitor, and aprotinin have also been tested for their effects on insulin metabolism. While camostat mesylate and bacitracin improved insulin bioavailability in the large intestine, they did not affect absorption in the small intestine.^489^ Rapid dilution, low potency, and digestion-related issues can limit the effectiveness of these inhibitors. Higher doses could overcome these limitations but raise safety concerns, including pancreatic hypertrophy, hyperplasia, and nephrotoxicity. Additionally, the pancreas may counteract inhibitor effects by increasing protease secretion, and these inhibitors may also disrupt the absorption of other proteins, affecting overall gastrointestinal metabolism.

Additionally, alternative delivery routes for GLP-1 receptor agonists have been explored. MannKind Corporation developed an inhalable GLP-1 powder, MKC253, using the Technosphere platform. In this method, GLP-1 is adsorbed onto fumaryl diketopiperazine (FDKP) microparticles with a size range of 2–5 μm. Upon reaching the lungs, FDKP dissolves, allowing GLP-1 to be absorbed into the systemic circulation.^490^ Although early Phase 1 trials showed potential benefits, such as improved systemic delivery and reduced gastrointestinal side effects, further development was discontinued due to strategic challenges and inconsistent therapeutic effects.

In addition to all the strategies mentioned above, macroscopic materials, often classified as medical devices, have been widely utilized in oral drug delivery systems. Some of these systems incorporate combinations of these materials, including osmotic capsules and microneedles. For instance, the osmotic-controlled release oral delivery system (OROS), an FDA-approved technology, is designed to provide controlled, extended release of drugs over time. It consists of a rigid capsule containing a core with the active pharmaceutical ingredient and a semipermeable membrane that governs the drug release rate.^491^ Upon contact with the gastrointestinal tract, the OROS capsule absorbs water, causing the core to expand. The osmotic pressure generated gradually releases the drug through a small hole in the membrane. This technology is particularly advantageous for drugs that require stable plasma concentrations over prolonged periods, reducing the frequency of dosing and minimizing fluctuations in drug levels. It is unaffected by variables such as pH, food intake, and intestinal environment. Drugs delivered through OROS include extended-release formulations like Concerta (methylphenidate for ADHD) and Cardura XL (doxazosin for hypertension). However, OROS has limitations, such as the complexity of manufacturing and potential gastrointestinal irritation or even blockage of the gastrointestinal tract due to the prolonged release of certain drugs.

More recently, innovative drug delivery technologies, such as coated or integrated microneedles, have been developed for peptide delivery. One notable example is the RaniPill, developed by Rani Therapeutics.^492,493^ This capsule sheds its cellulose coating upon reaching the intestine, triggering the inflation of a balloon inside the capsule. This inflation creates enough pressure to push the microneedles out of the capsule, allowing them to penetrate the intestinal wall and deliver the drug directly into the bloodstream. This technology has demonstrated over 50% oral bioavailability in preclinical studies for insulin and adalimumab.^494^ However, its utility is limited by the relatively small drug payload (3–5 mg per pill). Although the first-in-human safety study reported no adverse events, the small payload restricts the broader application of this technology.

Other emerging microneedle technologies, including self-orienting millimeter-scale applicators (SOMA), are in development (Figure 33). SOMA device is designed to deliver its drug payload to the stomach lining via a fluid-triggered dissolution process that deploys a spring mechanism to inject the drug.^495^ Although initial studies have shown promise, with SOMA capable of delivering up to 0.5 mg of insulin per device, advancements have led to a new version capable of delivering doses up to 4 mg. This updated SOMA device demonstrates up to 80% bioavailability within hours of administration, making it a promising candidate for the delivery of both small molecules and monoclonal antibodies.^496^ Additional optimization of the device is required to reduce the capsule size, increase drug loading capacity, and further minimize the risk of gastrointestinal obstruction.

**Figure 33:**
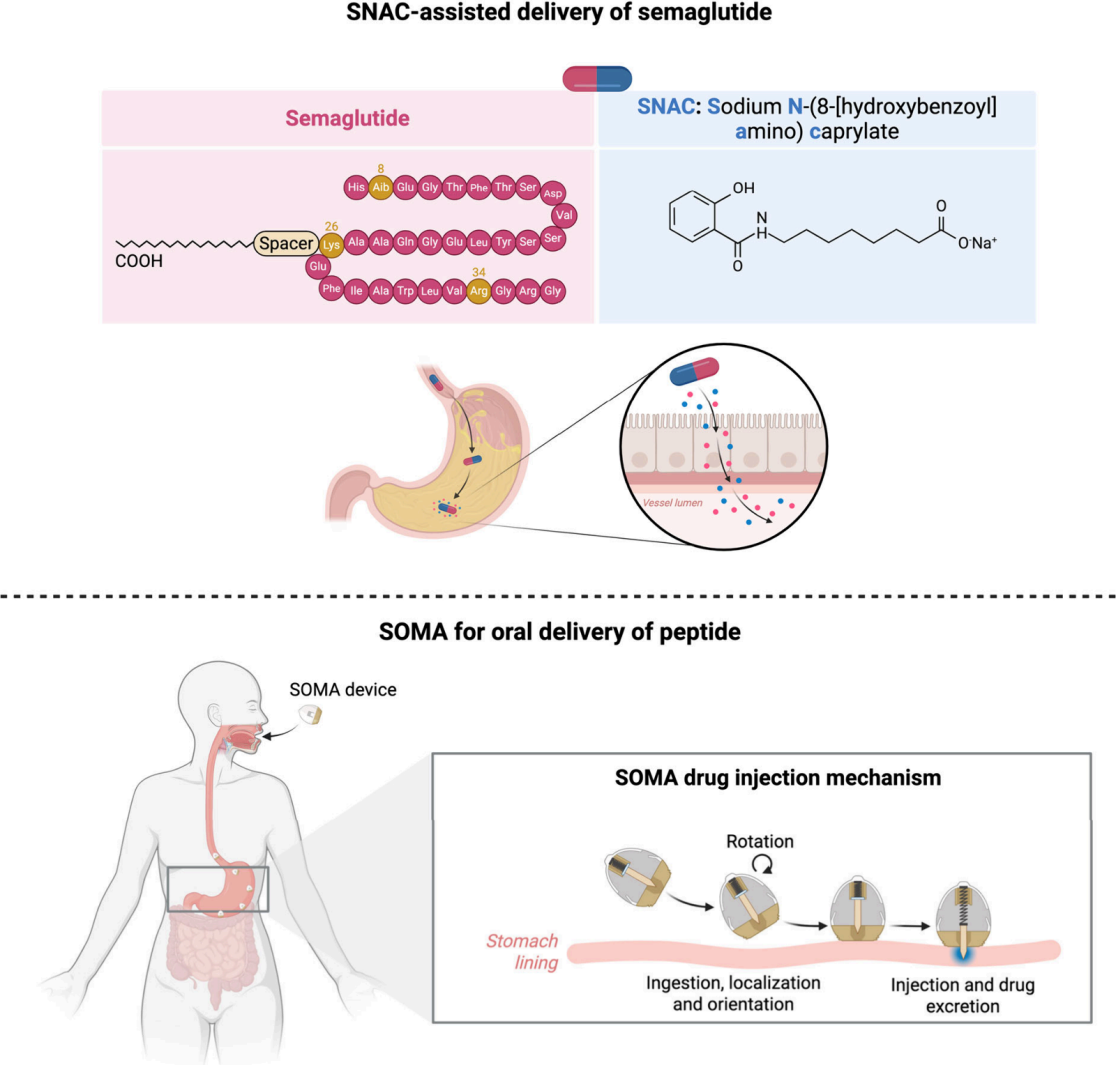
Top: Structure of SNAC, an absorption enhancer by locally increasing pH and promoting transcellular transport across the gastric epithelium. Bottom: Schematic representation of emerging microneedle technologies SOMA. The SOMA device is engineered to deliver drug payloads directly to the stomach lining through a fluid-triggered dissolution process that activates a spring-loaded injection mechanism. Created in BioRender.

A widely adopted strategy to enhance peptide permeability involves structural modification, most notably cyclization. Cyclization confers increased proteolytic stability, protecting peptides from enzymatic degradation and thereby facilitating improved intestinal absorption. Pye and colleagues conducted a study to investigate the effects of molecular size and lipophilicity on membrane permeability, utilizing libraries of cyclic peptides ranging from octapeptides to decapeptides, with molecular weights (MWs) between 800 and 1200 Da.^497^ To minimize the influence of intramolecular hydrogen bonding on conformational preferences and membrane permeability, the researchers fully *N*-methylated the backbone amide bonds. Each peptide was designed to include one tyrosine (Tyr) and one proline (Pro) residue, with the remaining residues limited to amino acids featuring either natural or non-natural aliphatic side chains. This design constraint reduced the impact of polar and charged groups, enabling a focused examination of how molecular size affects permeability. For assessing membrane permeability, the team employed the parallel artificial membrane permeability assay (PAMPA) and utilized an MDCK cell clone that expressed low levels of P-glycoprotein to minimize transporter-mediated efflux effects. This study found a significant decrease in passive permeability for peptides exceeding a molecular size threshold of approximately 1000 Da. This finding suggests a fundamental limitation in cellular permeability for larger molecules, challenging traditional solubility-diffusion theories and proposing a potential mechanism involving diffusion through polymer networks.

Additionally, the research underscored the delicate interplay between lipophilicity and size in achieving optimal cell permeability and aqueous solubility, particularly within the challenging MW range of 700–1000 Da. These observations correlate with previous data indicating that few orally administered drugs and clinical candidates exceed MWs of 1000 Da. Overall, the findings extend beyond cyclic peptides to include various classes of cell-permeable and orally administered drugs. They highlight the importance of molecular flexibility in adapting to physiological conditions, thereby integrating aqueous solubility, cell permeability, and efficient target binding. Incorporating this adaptable behavior into drug design may facilitate the discovery of larger drugs that expand the boundaries of cell-permeable drug space.

In the early 2000s, a surge of companies began exploring the potential of constrained peptides, motivated by the belief that these molecules could target previously inaccessible intracellular sites. Technologies surrounding constrained peptides, developed by various biotech firms, are increasingly attracting interest from larger pharmaceutical companies. For instance, PeptiDream has transferred its technology platform to major pharmaceutical companies, such as Merck, Lilly, Bristol-Myers Squibb, Novartis, Genentech and Astellas, to enhance their drug discovery initiatives.^498^ This platform combines advanced methods to generate macrocyclic peptides and screen them for potential drug candidates. Developed from RaPID, the groundbreaking work of the Suga team in Japan, the platform enables the rapid and efficient identification of novel compounds targeting specific proteins. In addition to utilizing the 20 standard amino acids, the technology incorporates more than 3,000 nonstandard amino acids into macrocyclic peptides. This allows for the creation of libraries containing trillions of structurally diverse peptides, providing exceptional flexibility for various applications.^499^

Innovative startups continue to push the boundaries in this field. For example, FogPharma is developing next-generation stapled peptides as miniproteins. Their efforts focus on advancing simple macrocycles, stapled peptides, and peptides with multiple loops, all designed to mimic critical binding epitopes of proteins, including β-hairpins and α-helices. Such capabilities allow these constrained peptides to disrupt targets that are often difficult for existing small molecules or biological therapies to affect.^498^

Developing strategies for efficient cellular peptide delivery and creating orally bioavailable peptide therapeutics proved to be complex. Challenges related to pharmacokinetics, manufacturing, and immunogenicity—particularly when peptide lengths exceed approximately 15 amino acids—have introduced further barriers, impeding progress in the field.^498,500^

## 8 Conclusions and Future Outlook

Although the first peptide-based drug, insulin, was discovered in 1921, it took several decades for the industrial development of peptide drugs to gain real momentum. The past three decades have witnessed peptides taking center stage, particularly in the treatment of diabetes and cancer. Numerous advancements have been made in developing synthetic methodologies capable of producing peptides with high purity and efficacy.

Peptide therapeutics occupy an intermediate position between small molecules and biologics, requiring specialized expertise and tailored approaches for their synthesis and purification. While peptides share certain attributes with proteins, their production demands distinctly different methodologies, fostering the growth of a specialized branch of medicinal chemistry focused on peptide discovery and optimization.

This field provides tools for refining peptide structures and pharmacological properties. Nevertheless, achieving an ideal peptide drug with simplified administration remains an ongoing challenge, and innovations in both delivery and stabilization are critical to broadening peptide therapeutic applications.

Today, the field has moved well beyond merely reproducing natural peptides; peptide engineering allows the creation of novel, improved, and more effective peptides, thanks to continuous innovations in both chemical synthesis and molecular design. This shift marks a pivotal evolution from natural mimicry to true molecular innovation. These include the development of new protecting groups, coupling reagents and hybridization of SPPS and LPPS, enabling the synthesis of increasingly complex peptides incorporating non-natural amino acids.

While new synthetic methodologies were being developed, modern biotechnological techniques based on genetic engineering were also introduced. These approaches now complement and enhance chemical methods, enabling more efficient and versatile peptide production. In particular, genetic engineering has yielded excellent results. Among its advantages are scalability, allowing continuous production of peptidomimetics without the need for costly chemical reagents or labor-intensive synthesis steps; the use of expanded genetic codes, allowing the incorporation of non-natural amino acids; fewer synthesis steps, enabling the production of complex peptidomimetics with greater automation and fewer manual interventions; high reproducibility once a genetic construct is optimized; and the ability to introduce post-translational modifications (e.g., phosphorylation, glycosylation) that add complexity and functionality sometimes unattainable through synthetic methods.

However, despite these advances, genetic engineering is not without limitations. While it offers the advantage of scalable peptide production at a relatively low cost for less complex peptides, challenges remain when it comes to optimizing expression systems for highly complex or hydrophobic peptides. These peptides tend to aggregate, becoming insoluble, which makes them difficult to express and purify effectively, especially at industrial scales. Purification processes can still be costly and labor-intensive, and the need for specialized reagents can drive up expenses. On the other hand, classic synthetic chemistry excels in providing precise control over peptide design. It allows the incorporation of non-natural amino acids, giving researchers complete freedom to tailor the sequence, composition, and stereochemistry without being constrained by the limitations of the genetic code. This flexibility is a key strength of synthetic chemistry, but it does come with its own set of challenges. Synthetic peptide production can be labor-intensive, time-consuming, and expensive, particularly when scaling up for industrial production of large peptides.

Both genetic engineering and synthetic chemistry have their distinct advantages, and neither approach is universally superior. Genetic engineering shines in scalable, cost-effective peptide production, while synthetic chemistry remains unparalleled in precision and the incorporation of non-natural elements. The choice of method often depends on specific project requirements and the balance between cost, scalability, and customization.

An additional advantage of biotechnological approaches lies not only in peptide production but also in drug screening. Systems like RaPID exemplify this capability, allowing for the rapid discovery of effective sequences against specific diseases. Biotechnologies contribute not only to production and drug discovery, but offer methods that are inherently more sustainable and “green” compared to purely chemical synthesis.

Determining whether synthetic or biotechnological approaches are “better” is complex. Each method complements the other, and hybrid approaches combining chemical synthesis with genetic engineering may offer the best of both worlds, leveraging the strengths of each technique. For instance, synthetic modifications could be introduced after expression, merging the biological efficiency of genetic engineering with the chemical versatility of synthesis. Alternatively, peptides could be synthesized in fragments and then assembled using enzymatic methods, similar to the process used in CEPS.

The past decade has witnessed significant successes in peptide therapeutics, notably glucagon-like peptide-1 (GLP-1) analogues, which have revolutionized diabetes and obesity management. Among these, semaglutide (marketed as Ozempic) emerged as the top-selling GLP-1 agonist in 2023, with 2024 sales projections exceeding $16 billion. Combined forecasts for semaglutide-based therapies, including Rybelsus (oral semaglutide) and Wegovy (for obesity), are expected to surpass $28 billion in 2024. Beyond diabetes, peptides have shown promise across diverse therapeutic areas, including pain management, infectious diseases, oncology, and diagnostics. Despite these advances, peptide therapeutics still face significant barriers to entry in some fields, notably neurology.

Currently, no peptide-based therapeutics have been approved for neurological diseases, largely due to the challenge of crossing the blood-brain barrier (BBB). Antibodies have demonstrated greater success in this domain, owing to engineered transport mechanisms. No broadly reliable method yet exists for consistent peptide delivery across the BBB. Emerging strategies, such as peptides derived from viral proteins, offer promising solutions by leveraging natural mechanisms of BBB penetration. Innovations in receptor-binding neuropeptides could provide cost-effective alternatives to antibody-based therapies. Another promising avenue involves targeting the gut-brain axis, though this introduces the additional challenge of overcoming gastrointestinal barriers. While significant research effort is directed toward these solutions, the clinical translation of such strategies remains in its infancy.

Antibodies have established a dominant position across therapeutic areas such as oncology, autoimmune diseases, and infectious diseases, due to their ability to precisely target specific proteins or cells. Notable examples include pembrolizumab (Keytruda) and adalimumab (Humira), both among the top-selling antibody therapeutics in 2023. Although peptides are gradually expanding their clinical footprint, they are unlikely to replace antibodies in areas where long systemic half-life and structural robustness are critical. Instead, peptides will find niche applications by targeting intracellular pathways inaccessible to antibodies, offering a complementary rather than competitive therapeutic approach.

Delivery remains a pivotal challenge in peptide drug development. Most peptide therapeutics are administered via injection—a route that, while effective, is less preferred by patients. Oral delivery presents significant hurdles, as peptides must withstand the harsh gastrointestinal environment. Achieving oral bioavailability often requires much higher doses, increasing production costs substantially. While cyclic peptides offer greater stability for oral delivery, their bioavailability remains limited. Despite these challenges, the success of injectable peptides in improving outcomes for diabetes and cancer has redefined the concept of the “ideal” drug: oral bioavailability is desirable, but no longer an absolute requirement. Membrane permeability and structural stability continue to be major obstacles. Strategies such as conjugation with cell-penetrating peptides (CPPs) and prodrug development have shown promise, but no broadly applicable solution has yet emerged.

Despite remarkable progress, peptide-based drugs continue to face significant challenges limiting their widespread application. Advances in chemical modifications, formulation technologies, and delivery systems have substantially improved peptide stability, bioavailability, and pharmacokinetics. Nevertheless, peptides remain susceptible to enzymatic degradation, short systemic half-lives, immunogenicity, off-target effects, and manufacturing complexity.

Future research directions will likely emphasize hybrid approaches combining the precision of synthetic chemistry with the scalability of biotechnology. Novel delivery platforms, such as nanoparticle-based carriers, viral vector systems, and next-generation oral formulations, will be critical to expanding the clinical applications of peptides. Moreover, deeper exploration of intracellular targets, modulation of the gut-brain axis, and improvements in BBB-penetrating technologies could open entirely new therapeutic landscapes.

In the long term, peptide therapeutics are poised to complement, rather than replace, antibodies and small molecules, carving out their own essential role within the increasingly sophisticated toolbox of modern medicine. Their success will depend on striking the right balance between biological complexity, therapeutic efficacy, patient convenience, and manufacturing feasibility—a formidable but exciting scientific frontier.

## Supplementary Material



## Notes

Peptides approved for bone health and management of calcium level, approved peptides mimicking oxytocin and vasopressin hormones, α-MSH, or GHRH, approved peptides for the treatment of cardiovascular diseases, and other peptides approved by the FDA (PDF)
