# Supplementary material for: Advances in Peptidomimetics for Next-Generation Therapeutics: Strategies, Modifications, and Applications
Source: Chem Rev. 2025 Jul 23;125(15):7099–166. doi: 10.1021/acs.chemrev.4c00989 (PMC12355721; doi:10.1021/acs.chemrev.4c00989)
Supplement: Supplementary file 1 [file cr4c00989_si_001.pdf]

Supporting information

Advances in Peptidomimetics for Next-Generation Therapeutics: Strategies, Modifications, and Applications

Lucia Lombardi,[1]* Valentina Del Genio,[2] Fernando Albericio,[3] Daryl R. Williams[1]

*E-mail: l.lombardi@imperial.ac.uk

1 Department of Chemical Engineering, Imperial College London, South Kensington, London SW7 2AZ, UK
2 Department of Pharmacy, University of Naples Federico II, Via Domenico Montesano 49, 80138 Naples, Italy
3 School of Chemistry and Physics, University of KwaZulu-Natal, Durban 4001, South Africa; CIBER-BBN, Networking Centre on Bioengineering, Biomaterials; Nanomedicine and Department of Organic Chemistry, University of Barcelona, 08028 Barcelona, Spain

1. Bone and calcium

Recombinant technology has also produced other peptide hormones, such as calcitonin and parathyroid hormone (PTH) (Table S1).[1]

Calcitonin (Figure S1), an amidated peptide consisting of 32 amino acids, was first discovered in dogs in 1961. Cysteines at positions 1 and 7 form a disulfide bridge. Calcitonin primarily functions to lower calcium levels in cases of hypercalcemia and inhibits bone resorption by osteoclasts, making it useful in the treatment of osteoporosis. Salmon calcitonin, which shares 50% amino acid identity with human calcitonin, was found to be more potent. It received FDA approval in 1978 for parenteral administration and in 1995 as a nasal spray, with a recombinant form following in 2005.[2]

Several oral formulations have reached phase III clinical trials. One formulation uses an acid-resistant enteric coating and includes citric acid to protect against intestinal proteases and facilitate paracellular transport. Another formulation employs a permeability enhancer, 5-CNAC, known as SMC021. However, neither formulation has demonstrated significant improvement in efficacy, which has diminished enthusiasm for developing an oral calcitonin.

PTH, by contrast, is a longer peptide of 84 residues and has the opposite effect of calcitonin, promoting calcium release into the bloodstream by mobilising bone stores. In 2002, a shorter version of PTH (Figure S1) with only 34 amino acids was approved for osteoporosis treatment under teriparatide. This formulation includes only the bioactive region of PTH and was the first approved peptide therapy that promotes bone formation rather than inhibiting bone resorption. In 2015, a full-length form of PTH was approved for the treatment of hypoparathyroidism.

Achieving sufficient bioavailability for oral PTH formulations remains a major challenge.

Synthetic analogues of PTH-related protein (PTHrP), such as abaloparatide (approved in 2017) and palopegteriparatide (approved in 2024), have also been developed.[3]

Table S1. Peptides approved for bone health and management of calcium level

Generic name	Brand name	Drug class	FDA first approval year	Company	Therapeutic indication	Route
calcitonin	Calcimar, Miacalcin, Calsyn, Caltine, Salco	salmon calcitonin	1978	Sanofi-Aventis, Par Pharm, Apotex, Mylan Ireland, Ferring, Sandoz-Novartis, Genmedix	osteoporosis, hypercalcaemia	SC
calcitonin	Miacalcin, Fortical	salmon calcitonin	1995	Novartis, Upsher-Smith	osteoporosis, hypercalcaemia	IN
calcitonin	Cibalcin	human recombinant PTH	2005	Novartis	osteoporosis, hypercalcaemia	IV
teriparatide	Bonsity, Forteo	PTH analogue	2002	Pfenex	osteoporosis	IV

PTH	Natpara	recombinant PTH	2015	NPS Pharmaceuticals	hypocalcaemia, hypoparathyroidism	SC
abaloparatide	Tymlos	PTHrP analogue	2017	Radius Health	osteoporosis	IV
palopegteriparatide	Yorvipath	PTHrP analogue	2024	Ascendis Pharma	osteoporosis	SC
etelcalcetide	Parsabiv	calcimimetic	2016	Amgen	secondary hyperparathyroidism	IV

IV: intravenous, IN: intranasal, SC: subcutaneous.

2. Oxytocin and Vasopressin Analogues

Natural oxytocin (Figure S1), introduced in 1980 for labour induction, remains the preferred agent for this purpose, administered intravenously to initiate and enhance uterine contractions. In this setting, oxytocin's short half-life poses no issue due to the high density of oxytocin receptors in the uterus. It is advantageous given the potential off-target effects on closely related vasopressin receptors (e.g., those in the cardiac vasculature), which, if activated, could lead to complications during childbirth.

Carbetocin, an oxytocin analogue in which a thioether bond replaces the disulfide bond, demonstrates enhanced proteolytic stability and is used to prevent uterine atony and manage postpartum bleeding (Table S2).

Vasopressin (Figure S1), along with its early analogue lypressin, was initially used to treat central diabetes insipidus but was later replaced by desmopressin. Desmopressin offers significantly improved proteolytic stability due to the substitution of L-Arg with D-Arg at position 8, preventing trypsin-like cleavage of the Pro-Arg peptide bond. Desmopressin is used to treat diabetes inspidus and nighttime bed-wetting. Notably, desmopressin was one of the first peptides suitable for both oral and intranasal administration, with an extended half-life of 1.5 to 2.5 hours. Despite the synthesis of thousands of oxytocin and vasopressin analogues, issues with receptor selectivity and interspecies receptor variation (notably between rodents and humans) have limited the development of superior analogues. Notable exceptions include phenypressin and terlipressin, the latter modified with a tri-glycine tail that further increases its half-life.[4]

Table S2. Approved peptides mimicking oxytocin and vasopressin hormones

Generic name	Brand name	Drug class	FDA first approval year	Company	Therapeutic indication	Route
oxytocin	Pitocin, Syntocinon	peptide hormone	1980	Endo	initiation of uterine contractions, prevent postpartum hemorrhage	IV
carbetocin	Pabal, Duratocin, Lonactene	oxytocin analogue	2019	Ferring	prevent postpartum hemorrhage	IV, IM, IN
desmopressin	DDAVP, Defirin, Desmopressin Acetate, Minirin, Minirinmelt, Octim, Stimate, Concentraid	vasopressin analogue	1978	Apotex, Bausch & Lomb, Barr Labs, Behring, Ferring, Hospira, Pharmaceutique Noroit, Sanofi-Aventis, Teva, Watson	nighttime bed-wetting, central diabetes insipidus	IN
terlipressin	Terlivaz, Glypressin	vasopressin analogue	2022	Mallinckrodt	hepatorenal syndrome, esophageal variceal bleeding	IV
atosiban	Tractocile, Antocin	oxytocin and vasopressin antagonist	2000	Ferring	delaying imminent pre-term birth in pregnant adult women	IV

IV: intravenous, IN: intranasal, SC: subcutaneous.

3. α-MSH analogues

α-Melanocyte-stimulating hormone (α-MSH) (Figure S1) is an endogenous hormone and neuropeptide in the melanocortin family. This 13-amino-acid acetylated peptide amide is critical for melanin synthesis via melanogenesis and maintaining energy balance and regulating sexual activity. Several FDA-approved analogues of α-MSH, including afamelanotide, bremelanotide, and setmelanotide, have shown therapeutic efficacy across various conditions (Table S3, Figure S1).

Table S3. Approved peptides mimicking α-MSH

Generic name	Brand name	Drug class	FDA first approval year	Company	Therapeutic indication	Route
afamelanotide	Scenesse	α-MSH analogues	2019	Clinuvel	erythropoietic protoporphyria to increase melanin production	SC
bremelanotide	Vyleesi	α-MSH analogues	2019	Cosette	hypoactive sexual desire disorder in premenopausal women	SC
setmelanotide	Imcivree	α-MSH analogues	2020	Rhythm	obesity in patients with rare genetic disorders of obesity	SC

SC: subcutaneous.

Afamelanotide, a 13-amino-acid synthetic analogue of α-MSH, incorporates modifications such as norleucine (Nle) and D-phenylalanine at positions 4 and 7 (substituting methionine and L-phenylalanine) and features N-terminal acetylation. These modifications substantially enhance proteolytic stability and boost potency approximately 1,000-fold by extending the peptide's half-life. A shorter cyclic analogue, melanotan-II, was also studied for skin pigmentation at lower doses but was withdrawn from trials due to side effects. In 2019, afamelanotide received FDA approval to alleviate painful photosensitivity in patients with erythropoietic protoporphyria, a rare disorder.[5]

The FDA also approved bremelanotide in 2019 to treat hypoactive sexual desire disorder in premenopausal women. Bremelanotide's structure includes a cyclic amide bond between the β-carboxylic acid of aspartic acid (Asp) and the ε-amino group of lysine (Lys), which serves as the C-terminal residue. The cyclic conformation and D-phenylalanine incorporation enhance stability, while acetylation modulates protein functions, including enzymatic activity.[2, 6]

More recently, setmelanotide, another synthetic cyclic analogue of α-MSH, was approved to treat obesity associated with rare genetic disorders, including pro-opiomelanocortin deficiency, proprotein subtilisin/kexin type 1 deficiency, and leptin receptor deficiency. Setmelanotide’s design includes a disulfide bridge between Cys2 and Cys8 and two D-amino acid residues, further supporting its structural stability and therapeutic efficacy.[6]

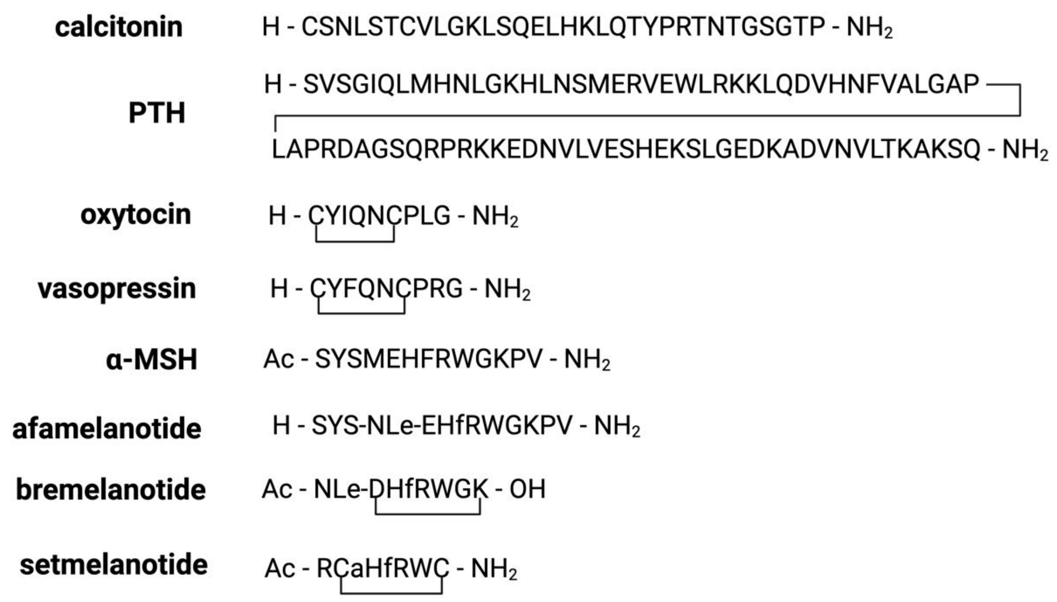

Figure S1. Sequences of some natural peptides and α-MSH analogues approved by FDA.

4. GHRH analogues

Ghrelin is a 28-amino acid peptide that stimulates the secretion of growth hormones by interacting with a specific receptor known as the growth hormone secretagogue receptor 1a (GHS-R1a). Activation of this receptor by agonists has various pharmacological applications, including the treatment of growth retardation, gastrointestinal dysfunction, and impaired body composition.[7] There are four FDA-approved analogues of ghrelin: sermorelin (29 amino acids), mecasermin (70 amino acids), tesamorelin (a peptide with a C6 chain at position 3), and macimorelin (a shorter peptide incorporating D-amino acids) (Table S4, Figure S2).[8]

Table S4. Approved peptides mimicking GHRH

Generic name	Brand name	Drug class	FDA first approval year	Company	Therapeutic indication	Route
sermorelin	Geref	GHRH Analogues	1990	EMD Serono	lipodystrophy in HIV-infected adults	SC
mecasermin	Increlex	GHRH Analogues	2005	Ipsen	primary IGF-1 deficiency in children	SC
tesamorelin	Egrifta	GHRH Analogues	2010	Kendle International	lipodystrophy in HIV-infected adults	SC
macimorelin	Macrilen	GHRH Analogues	2017	Novo	diagnosis of adult growth hormone deficiency	O

SC: subcutaneous, O: orally.

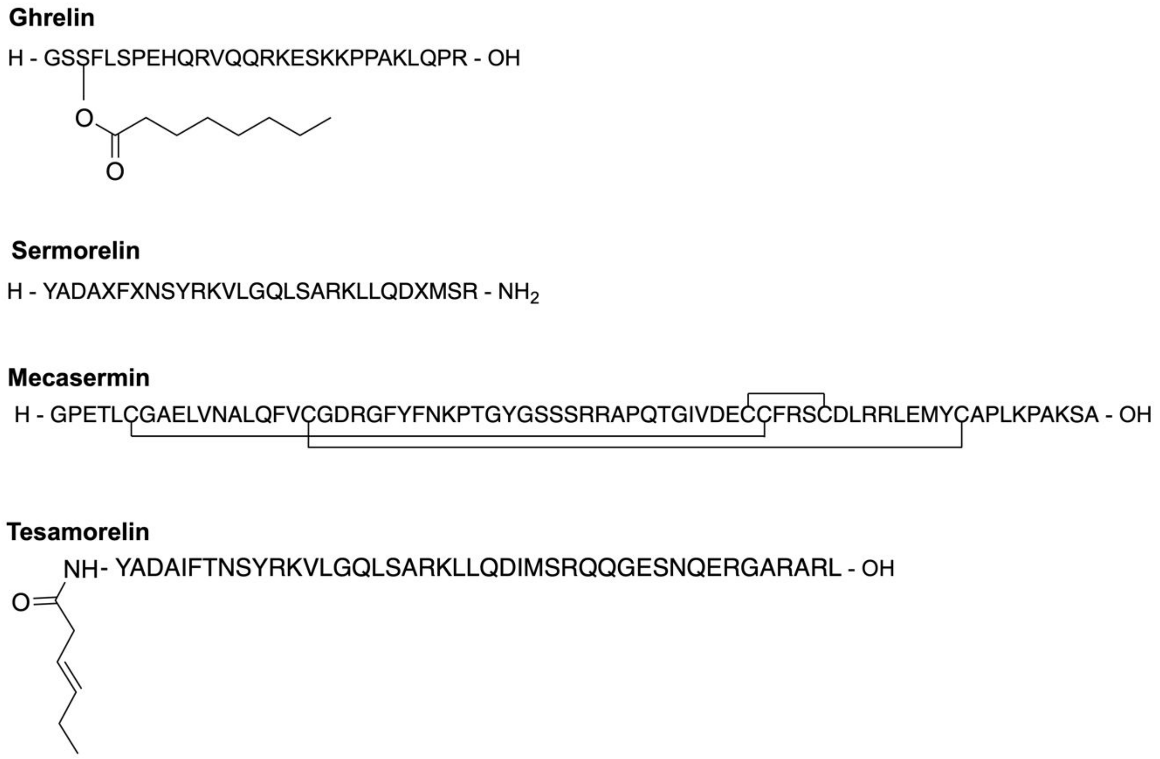

Figure S2. Sequences of ghrelin and its analogues.

5. Immunosuppressants

A significant array of peptides has been derived from diverse sources, including bacteria, fungi, plants, and animals, demonstrating superior therapeutic efficacy compared to their human-derived counterparts. These peptides exhibit enhanced selectivity, potency, and stability in vivo. For instance, cyclosporine, obtained from fungi, and the thrombin inhibitor bivalirudin, derived from the saliva of medicinal leeches, exemplify these characteristics.

Cyclosporine (Figure S3) was first isolated from fungi in 1970 during a project at Sandoz aimed at discovering novel antiviral agents. Subsequently, its immunosuppressive properties were identified, leading to its approval for clinical use in 1983. This 11-residue peptide is neutral and hydrophobic in nature. Its cyclic structure, combined with N-methylations at positions 7 and 11, the presence of unusual amino acids at positions 1 and 2, and a D-amino acid at position 8, confer remarkable stability against proteolytic degradation, rendering it highly suitable for oral administration.[9]

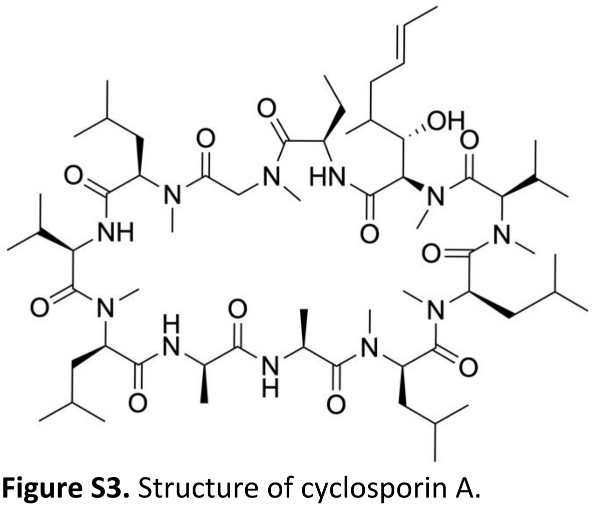

Figure S3. Structure of cyclosporin A.

Other peptides employed as immunosuppressants include pegcetacoplan and zilucoplan (Table 5).

Table S5. Peptides approved for the treatment of cardiovascular diseases

Generic name	Brand name	Drug class	FDA first approval year	Company	Therapeutic indication	Route
cyclosporine	Veye, Gengraf, Neoral, Sandimmune, Verkazia	immunosuppressant	1983	Sandoz and others	transplant rejection prevention and autoimmune disorders	O
voclosporin	Lupkynis	immunosuppressant	2021	Aurinia Pharmaceuticals	lupus nephritis	O
pegcetacoplan	Empaveli	immunosuppressant	2021	Apellis Pharmaceuticals	paroxysmal nocturnal hemoglobinuria	SC
zilucoplan	Zilbrysq	immunosuppressant	2023	UCB	generalized myasthenia gravis	SC

SC: subcutaneous, O: orally.

Pegcetacoplan is a pegylated, C3-targeted cyclic peptide that modulates complement signalling. It is utilised in the treatment of paroxysmal nocturnal hemoglobinuria (PNH), a rare and serious blood disorder characterised by the destruction of red blood cells. This compound consists of two 13-mer peptides covalently linked through a linear PEG molecule, utilising the ε-amino group of lysine.[10]
Zilucoplan is developed using an mRNA display platform by Ra Pharmaceuticals. It is a 15-amino acid macrocyclic peptide that inhibits C5 and has received FDA approval for the treatment of generalised myasthenia gravis in adults who are positive for anti-acetylcholine receptor antibodies. Zilucoplan is characterised as a cyclic peptide complement inhibitor with a lactam bond formed between Lys1 and Asp6, and features a pendant Nε-palmitoyl-γ-L-glutamyl moiety extending from its C-terminal lysine.[11]

6. Heart and vascular system

6.1 Icatibant

Icatibant (Table 6) is a 10-amino acid peptide that is a competitive and selective antagonist of the bradykinin B2 receptor. It received FDA approval in 2011 for the symptomatic treatment of acute attacks in hereditary angioedema. Bradykinin, an endogenous nonapeptide hormone, is produced in response to various injuries and inflammatory stimuli. It is a potent pain inducer and mediates blood vessel dilation and smooth muscle contraction. However, the therapeutic application of native bradykinin is limited due to its short half-life. Several enzymes rapidly degrade bradykinin, including angiotensin I-converting enzyme (ACE), which nearly eliminates bradykinin during a single passage through the pulmonary circulation. Icatibant acts by competitively inhibiting bradykinin from binding to the B2 receptor. This action is critical in treating hereditary angioedema, a condition characterised by intermittent swelling of the skin, upper airways, genitourinary tract, and gastrointestinal mucosa due to elevated bradykinin levels arising from a deficiency or dysfunction of C1 esterase inhibitor. A key discovery in antagonist design was that replacing Pro7 with D-Phe in bradykinin resulted in a critical shift from agonist to antagonist activity, inspiring further development. Early antagonists showed limited enzymatic stability, but subsequent modifications improved stability without sacrificing selectivity or potency. Key structural optimizations included adding D-Arg at the N-terminus to guard against endopeptidase degradation, substituting Pro with Hyp at position 3, and replacing Phe at positions 5 and 8 with isosteric β-(2-thienyl)-alanine (Thi) residues to enhance resistance against ACE cleavage.[12]

6.2 Eptifibatide

Eptifibatide (Table 6) is a synthetic cyclic heptapeptide developed for the treatment of acute coronary syndrome, including heart attacks and unstable angina, by inhibiting platelet aggregation. This peptide was inspired by the structure of barbourin, a 73-amino acid peptide with six disulfide bonds that specifically inhibits GPIIb/IIIa, a receptor involved in platelet aggregation, found in the venom of the southeastern pygmy rattlesnake *Sistrurus miliarius barbouri*. Through identification and preservation of the KGD recognition motif—a variation of the RGD motif—in one of the disulfide loops of barbourin, the peptide was successfully truncated to a cyclic heptapeptide

without losing its selectivity or inhibitory activity. Subsequent structure-activity relationship studies further optimised the design. Key modifications included N-terminal deamination and the substitution of Lys with homo-Arg through guanylation, leading to further improvements in activity. Despite a relatively short in vivo half-life of 2.5 hours due to renal clearance, this characteristic is advantageous, allowing rapid recovery of clotting function after discontinuation of eptifibatide infusion therapy.[13]

6.3 Romiplostim

Romiplostim (Table 6) is a peptide-Fc fusion protein mimicking thrombopoietin (TPO), the natural hormone regulating platelet production. Approved in 2008, romiplostim increases platelet counts in patients with immune thrombocytopenic purpura (ITP) who do not respond to other treatments. The peptide component of romiplostim was identified through phage display, which enabled the selection of recombinant peptides that bind specifically to the TPO receptor. Notably, romiplostim has no sequence similarity to endogenous TPO, avoiding the issue of cross-reactive antibody production seen with earlier recombinant TPO products.[14]

6.4 Ecallantide

Ecallantide (Table 6) was developed by optimising a Kunitz domain, a protease inhibitor domain, through phage display to effectively inhibit kallikrein, an enzyme that activates bradykinin by cleaving its precursor kininogen. Like icatibant, ecallantide is approved for treating acute attacks of hereditary angioedema, receiving FDA approval in 2009.[15]

6.5 Hirudin and its analogues: Lepirudin and Bivalirudin

Hirudin, a 65-residue peptide isolated from the saliva of medicinal leeches, is a powerful anticoagulant and specific thrombin inhibitor. Its structure is rigid at the N-terminus due to four disulfide bonds, while the C-terminus is more flexible (Table 6). Lepirudin, an analogue of hirudin, includes some structural modifications, such as a leucine substitution at position 1 (L1I) and the removal of tyrosine sulfonation at residue 63. This analogue was approved for clinical use in 1998. In contrast, bivalirudin is a much shorter derivative, approved later, consisting of only 20 amino acids. It retains the active thrombin-inhibitory sequence D-Phe-Pro-Arg, linked by a Pro-$(Gly)_4$ linker to a dodecapeptide analogue of the C-terminal region of hirudin. Importantly, thrombin can slowly cleave the inhibitor sequence at the Arg-Pro site within this linker, partially restoring thrombin activity over time. This feature allows for regulated reactivation of thrombin, leading to controlled hemostasis following the therapeutic effect. Bivalirudin has a relatively short half-life of 20–30 minutes, which supports its use in clinical settings where rapid recovery of clotting ability is advantageous.[16]

Table S6. Peptides approved for the treatment of cardiovascular diseases

Generic name	Brand name	Drug class	FDA first approval year	Company	Therapeutic indication	Route
lepirudin	Refludan	thrombin inhibitor, hirudin analogue	1998	Bayer HealthCare	drug induced thrombocytopenia	IV
eptifibatide	Integrillin	glycoprotein platelet inhibitors	1998	Teva, Mulan Labs, Sagent, Eugia	to prevent blood clots or heart attack in people with severe chest pain	IV
bivalirudin	Angiomax	thrombin inhibitor, hirudin fragment	2000	The Medicines Company	drug induced thrombocytopenia	IV
icatibant	Firazyr, Sajazir	hereditary angioedema agents (bradykinin antagonist)	2011	Jerini	hereditary angioedema	SC
romiplostim	Nplate	thrombopoietin receptor agonist	2008	Amgen	immune thrombocytopenia to stimulate platelet production	SC

nesiritide	Natrecor	vasodilator	2001	Scios	severe heart failure	IV
vasopressin	Vasostrict	vasoconstrictor	2014	Endo	hypotension	IV
angiotensin II	Giapreza	vasoconstrictor	2017	La Jolla Pharma	hypotension	IV
lepirudin	Refludan	thrombin inhibitor, hirudin analogue	1998	Bayer HealthCare	drug induced thrombocytopenia	IV

IV: intravenous, SC: subcutaneous.

7. Miscellaneous

7.1 Guanylate cyclase-C agonists

Linaclotide is a potent, first-in-class agonist of guanylate cyclase 2C (GC-C) approved in 2012 for the treatment of irritable bowel syndrome with constipation (IBS-C) and chronic idiopathic constipation (Table 7).[17] This 14-amino acid hybrid peptide is structurally inspired by Escherichia coli heat-stable enterotoxins that cause diarrhoea and share similarities with the human hormones guanylin and uroguanylin. Linaclotide features three disulfide bonds, enhancing its GC-C agonist potency by stabilising it in an active conformation, which increases its binding affinity and stability within the gastrointestinal tract. Since GC-C is located on the surface of gastrointestinal epithelial cells, linaclotide can be taken orally. Due to its size, linaclotide does not cross the gut epithelium, thus minimising systemic exposure and off-target effects. Another GC-C agonist, plecanatide—a 16-amino acid peptide with two disulfide bonds and structurally similar to uroguanylin—was approved in 2017 for similar indications. Dolcanatide, similar to plecanatide with D-amino acid substitutions at both termini for enhanced stability, has completed Phase II trials for constipation treatment.

7.2 Lucinactant

Lucinactant (Table 7) is a non-pyrogenic synthetic pulmonary surfactant that combines two phospholipids, a fatty acid, and sinapultide, a 21-amino acid hydrophobic peptide (KL_4-KL_4-KL_4-KL_4-K, acetate) designed to mimic the activity of surfactant protein B.[18] It received FDA approval in 2012 for the treatment of respiratory distress syndrome (RDS) in infants. Delivered intratracheally, lucinactant compensates for the lack of natural surfactant in RDS patients, enhancing surface activity by reducing surface tension at the alveolar air-liquid interface and stabilising alveoli to prevent collapse at low transpulmonary pressures.

7.3 Etelcalcetide

Approved by the FDA in 2017 (Table 7), etelcalcetide is an octapeptide with seven D-amino acids and a unique disulfide linkage between a D-Cys and L-Cys residue.[19] This peptide acts by binding to calcium-sensing receptors on the parathyroid gland, reducing parathyroid hormone (PTH) secretion in patients with secondary hyperparathyroidism undergoing dialysis for chronic kidney disease. Incorporating D-amino acids enhances etelcalcetide's resistance to proteolytic degradation and lowers immunogenicity risk.

7.4 Teduglutide

Teduglutide, a 36-amino acid analogue of glucagon-like peptide 2 (GLP-2), was approved in 2012 for treating short bowel syndrome (Table 7). Teduglutide promotes mucosal growth and may aid in gastric emptying and secretion. It is produced recombinantly using *E. coli*, and an alanine-to-glycine substitution at position 2 renders it resistant to degradation by DPP-4, extending its half-life to 2–3 hours (compared to the native GLP-2 half-life of 7 minutes).[20] Teduglutide's bioactivity is significantly enhanced, with an 87% bioavailability upon subcutaneous administration. It binds to GLP-2 receptors primarily in the small and large intestine and is administered once daily by injection.

Table S7. Miscellaneous of other peptides approved by FDA

Generic name	Brand name	Drug class	FDA first approval year	Company	Therapeutic indication	Route
corticotropin	Acthar	extraction	1950	Mallinckrodt	acite exacerbations of multiple sclerosis	IV

cosyntropin	Cosyntropin, Cortosyn, Syncthen	adrenocorticotropin-releasing hormone (ACRH) analogues	1970	Amphastar, Sandoz-Novartis, Bioniche	diagnosis of adrenocortical insufficiency	IV
corticorelin	Acthrel	adrenocorticotropin-releasing hormone (ACRH) analogues	1996	Ferring	diagnosis of adrenocortical insufficiency	IV, IN
glatiramer	Copaxone	immunomodulator	1996	Teva	Multiple sclerosis	SC
linaclotide	Constella, Linzess	guanylate cyclase-C agonist	2012	Allergan	chronic idiopathic constipation, irritable bowel syndrome with constipation	O
plecanatide	Trulance	guanylate cyclase-C agonist	2017	Bausch Health	chronic idiopathic constipation, irritable bowel syndrome with constipation	O
ecallantide	Kalbitor	kallikren inhibitor	2009	Dyax Corporation	hereditary angioederma	SC
peginesatide	Omontys	erythropoiesis-stimulating agent	2012	Affymax	anemia chronic kidney diseases	IV, SC
lucinactant	Surfaxin	surfactant	2012	Discovery Labs	prevention of respiratory distress syndrome	IT
vosoritide	Voxzogo	C-type natriuretic peptide analogue	2021	BioMarin Pharmaceutical	achondroplasia	IV
sincalide	Kinevac	uncategorized agents	1976	Bracco	diagnosis of disorders of the gallbladder or pancreas	SC
teduglutide	Gattex, Revestive	miscellaneous GI agents (GLP-2 analogue)	2012	Tadeka	short bowel syndrome	SC
etelcalcetide	Parsabiv		2017	Amgen	Secondary hyperparathyroidism in patients with chronic kidney disease on hemodialysis	IV
motixafortide	Aphexda	CXCR4 inhibitor	2023	BioLineRx	hematopoietic stem cell mobilization for transplantation in patients with multiple myeloma	SC
glucagon	GlucaGen, Glucagon	peptide hormone	1998	Lilly, Novo Nordisk, ZymoGenetics	hypoglycemia, gastrointestinal motility inhibitor	IM, IV, SC
glucagon	Baqsimi	peptide hormone	2019	Lilly	hypoglycemia	IN, IM, SC
dasiglucagon	Zegalogue	glucagon analog	2021	Zealand	severe hypoglycemia	SC

IV: intravenous, IM: intramuscular, IN: intranasal, SC: subcutaneous, O: orally, IT: intrathecal.

References

(1) Inzerillo, A. M.; Zaidi, M.; Huang, C. L. Calcitonin: physiological actions and clinical applications. *J. Pediatr. Endocrinol. Metab.* **2004**, *17* (7), 931-940.
(2) Muttenthaler, M.; King, G. F.; Adams, D. J.; Alewood, P. F. Trends in peptide drug discovery. *Nat. Rev. Drug. Discov.* **2021**, *20* (4), 309-325.

(3) Haas, A. V.; LeBoff, M. S. Osteoanabolic Agents for Osteoporosis. *J. Endocr. Soc.* **2018**, *2* (8), 922-932.
(4) Manning, M.; Misicka, A.; Olma, A.; Bankowski, K.; Stoev, S.; Chini, B.; Durroux, T.; Mouillac, B.; Corbani, M.; Guillon, G. Oxytocin and Vasopressin Agonists and Antagonists as Research Tools and Potential Therapeutics. *J. Neuroendocrinol.* **2012**, *24* (4), 609-628.
(5) Minder, E. I.; Barman-Aksoezen, J.; Schneider-Yin, X. Pharmacokinetics and Pharmacodynamics of Afamelanotide and its Clinical Use in Treating Dermatologic Disorders. *Clin. Pharmacokinet.* **2017**, *56* (8), 815-823.
(6) Molinoff, P. B.; Shadiack, A. M.; Earle, D.; Diamond, L. E.; Quon, C. Y. PT-141: a melanocortin agonist for the treatment of sexual dysfunction. *Ann. N. Y. Acad. Sci.* **2003**, *994* (1), 96-102.
(7) Ishida, J.; Saitoh, M.; Ebner, N.; Springer, J.; Anker, S. D.; von Haehling, S. Growth hormone secretagogues: history, mechanism of action, and clinical development. *JCSM Rap. Commun.* **2020**, *3* (1), 25-37.
(8) Dhillon, S. Tesamorelin: a review of its use in the management of HIV-associated lipodystrophy. *Drugs* **2011**, *71* (8), 1071-1091. Guerlavais, V.; Boeglin, D.; Mousseaux, D.; Oiry, C.; Heitz, A.; Deghenghi, R.; Locatelli, V.; Torsello, A.; Ghe, C.; Catapano, F.; et al. New active series of growth hormone secretagogues. *J. Med. Chem.* **2003**, *46* (7), 1191-1203.
(9) Lipinski, C. A.; Lombardo, F.; Dominy, B. W.; Feeney, P. J. Experimental and computational approaches to estimate solubility and permeability in drug discovery and development settings. *Adv. Drug Del. Rev.* **1997**, *23* (1-3), 3-25.
(10) de Castro, C.; Grossi, F.; Weitz, I. C.; Maciejewski, J.; Sharma, V.; Roman, E.; Brodsky, R. A.; Tan, L.; Di Casoli, C.; El Mehdi, D.; et al. C3 inhibition with pegcetacoplan in subjects with paroxysmal nocturnal hemoglobinuria treated with eculizumab. *Am. J. Hematol.* **2020**, *95* (11), 1334-1343.
(11) Tang, G.-Q.; Tang, Y.; Dhamnaskar, K.; Hoarty, M. D.; Vyasamneni, R.; Vadysirisack, D. D.; Ma, Z.; Zhu, N.; Wang, J.-G.; Bu, C. Zilucoplan, a macrocyclic peptide inhibitor of human complement component 5, uses a dual mode of action to prevent terminal complement pathway activation. *Front. Immunol.* **2023**, *14*, 1213920.
(12) Cugno, M.; Tedeschi, A. Coagulation factor autoantibodies. In *Autoantibodies*, Elsevier, 2014; pp 499-509.
(13) Phillips, D. R.; Scarborough, R. M. Clinical pharmacology of eptifibatide. *Am. J. Cardiol.* **1997**, *80* (4A), 11B-20B.
(14) Bussel, J. B.; Soff, G.; Balduzzi, A.; Cooper, N.; Lawrence, T.; Semple, J. W. A Review of Romiplostim Mechanism of Action and Clinical Applicability. *Drug Des. Devel. Ther.* **2021**, *15*, 2243-2268.
(15) Duffey, H.; Firszt, R. Management of acute attacks of hereditary angioedema: role of ecallantide. *J. Blood Med.* **2015**, *6*, 115-123.
(16) Warkentin, T. E.; Koster, A. Bivalirudin: a review. *Expert Opin. Pharmacother.* **2005**, *6* (8), 1349-1371. Shammas, N. W. Bivalirudin: pharmacology and clinical applications. *Cardiovasc. Drug Rev.* **2005**, *23* (4), 345-360.
(17) Busby, R. W.; Kessler, M. M.; Bartolini, W. P.; Bryant, A. P.; Hannig, G.; Higgins, C. S.; Solinga, R. M.; Tobin, J. V.; Wakefield, J. D.; Kurtz, C. B.; et al. Pharmacologic properties, metabolism, and disposition of linaclotide, a novel therapeutic peptide approved for the treatment of irritable bowel syndrome with constipation and chronic idiopathic constipation. *J. Pharmacol. Exp. Ther.* **2013**, *344* (1), 196-206.
(18) Moen, M. D.; Perry, C. M.; Wellington, K. Lucinactant: in neonatal respiratory distress syndrome. *Treat. Respir. Med.* **2005**, *4* (2), 139-145; discussion 146-137.
(19) Cozzolino, M.; Galassi, A.; Conte, F.; Mangano, M.; Di Lullo, L.; Bellasi, A. Treatment of secondary hyperparathyroidism: the clinical utility of etelcalcetide. *Ther. Clin. Risk Manag.* **2017**, *13*, 679-689.

(20) Vipperla, K.; O'Keefe, S. J. Targeted therapy of short-bowel syndrome with teduglutide: the new kid on the block. *Clin. Exp. Gastroenterol.* **2014**, *7*, 489-495.
